# KLF4-SQSTM1/p62-associated prosurvival autophagy contributes to carfilzomib resistance in multiple myeloma models

**DOI:** 10.18632/oncotarget.4530

**Published:** 2015-06-19

**Authors:** Irene Riz, Teresa S. Hawley, Robert G. Hawley

**Affiliations:** ^1^ Department of Anatomy and Regenerative Biology, The George Washington University, Washington, DC, USA; ^2^ Flow Cytometry Core Facility, The George Washington University, Washington, DC, USA

**Keywords:** multiple myeloma, proteasome inhibitor, carfilzomib, autophagy, KLF4

## Abstract

Multiple myeloma (MM) is an incurable clonal plasma cell malignancy. Because of a high rate of immunoglobulin synthesis, the endoplasmic reticulum of MM cells is subjected to elevated basal levels of stress. Consequently, proteasome inhibitors, which exacerbate this stress by inhibiting ubiquitin-proteasome-mediated protein degradation, are an important new class of chemotherapeutic agents being used to combat this disease. However, MM cells still develop resistance to proteasome inhibitors such as carfilzomib. Toward this end, we have established carfilzomib-resistant derivatives of MM cell lines. We found that resistance to carfilzomib was associated with elevated levels of prosurvival autophagy, and Kruppel-like factor 4 (*KLF4*) was identified as a contributing factor. Expression levels as well as nuclear localization of KLF4 protein were elevated in MM cells with acquired carfilzomib resistance. Chromatin immunoprecipitations indicated that endogenous KLF4 bound to the promoter regions of the *SQSTM1* gene encoding the ubiquitin-binding adaptor protein sequestosome/p62 that links the proteasomal and autophagic protein degradation pathways. Ectopic expression of *KLF4* induced upregulation of *SQSTM1*. On the other hand, inhibitors of autophagy sensitized MM cells to carfilzomib, even in carfilzomib-resistant derivatives having increased expression of the multidrug resistance protein P-glycoprotein. Thus, we report here a novel function for KLF4, one of the Yamanaka reprogramming factors, as being a contributor to autophagy gene expression which moderates preclinical proteasome inhibitor efficacy in MM.

## INTRODUCTION

Multiple myeloma (MM), the second most common hematologic malignancy in the United States, is characterized by the accumulation of clonal plasma cells in the bone marrow [1]. Although MM patients initially respond to therapy, they inevitably relapse due to the development of treatment resistance [2]. The introduction of the proteasome inhibitor bortezomib has improved clinical outcome of MM patients [3]. However, MM cells acquire resistance to bortezomib by diverse mechanisms [4-8]. Carfilzomib is a second generation proteasome inhibitor that was approved in 2012 by the U.S. Food and Drug Administration for the treatment of relapsed/refractory MM patients who have received at least two prior therapies including bortezomib and an immunomodulatory drug [9]. While encouraging, the overall response rates to carfilzomib monotherapy in pivotal phase II studies were <25% (17.1% in the PX-171-004 study and 23.7% in the PX-171-003-A1 study, respectively) [10, 11], indicating that the majority of the MM cells that became resistant to bortezomib also exhibited resistance to carfilzomib. Thus, it is important to elucidate the underlying mechanisms of proteasome inhibitor resistance in MM and identify novel agents that will enhance therapeutic efficacy of this class of anti-MM drugs.

MM exhibits considerable genetic heterogeneity, with particular cytogenetic abnormalities such as the t(4;14) chromosomal translocation consistently associated with poor outcome [12]. In previous work, we identified upregulated expression of the *ABCB1*-encoded P-glycoprotein multidrug resistance efflux pump in t(4;14)-positive KMS-34 MM cells following short-term exposure to carfilzomib [13]. To gain further insight into the various mechanisms of carfilzomib resistance, we subjected another t(4;14)-positive MM cell line, KMS-11, together with KMS-34 to long-term selection in increasingly higher concentrations of the drug, deriving carfilzomib-resistant KMS-11/Cfz and KMS-34/Cfz cells, respectively. Of note, overexpression of *ABCB1* was not observed in KMS-11/Cfz cells. Gene set enrichment analysis (GSEA) [14] of microarray gene expression profiling data implicated increased expression of the pluripotency reprogramming factor Kruppel-like factor 4 (*KLF4*) [15] as contributing to carfilzomib resistance in both cases.

Depending on the type of cancer and genetic context, *KLF4* can act as either a tumor suppressor or an oncogene [16]. Notably, high levels of *KLF4* expression often occur in MM patients carrying the t(4;14) translocation [17, 18]. Moreover, it was previously reported that exogenous expression of *KLF4* partially protected some MM cell lines from cytotoxicity induced by the alkylating agent melphalan, and the partial protection was attributed to a proliferation block [19]. In the current study, we found that acquisition of carfilzomib resistance in both t(4;14)-positive MM cell line models was associated with reduced cell proliferation, decreased plasma cell maturation, and activation of prosurvival autophagy. Specifically, we show that KLF4 plays a role in prosurvival autophagy by binding to the promoter regions and increasing the expression of *SQSTM1* encoding the ubiquitin-binding adaptor protein sequestosome (SQSTM1/p62) that links the proteasomal and selective autophagic protein degradation pathways [20, 21]. Furthermore, resensitization of KMS-11/Cfz and KMS-34/Cfz cells to carfilzomib could be achieved by cotreatment with the autophagy inhibitor chloroquine [22].

## RESULTS

### KLF4 contributes to molecular phenotype of carfilzomib-resistant MM cells

KMS-11 and KMS-34 cells were exposed to stepwise increasing concentrations of carfilzomib over a period of 18 weeks: cells adapted to growth in 4 nM carfilzomib by 4 weeks, in 6 nM in another 6 weeks and in 12 nM after a further 8 weeks, albeit proliferating slower than parental cells not exposed to the drug. The resulting MM cell cultures, denoted KMS-11/Cfz and KMS-34/Cfz, respectively, retained resistance to carfilzomib even when tested after removal of selective pressure for approximately 8 weeks. In the current study, KMS-11/Cfz and KMS-34/Cfz cells were profiled for gene expression after 1 week of growth in the absence of carfilzomib together with parental KMS-11 and KMS-34 cells which had not been selected in the drug.

We employed GSEA to query gene sets in the Molecular Signature Database (MSigDB) to uncover processes or pathways shared between KMS-11/Cfz and KMS-34/Cfz cells that potentially contributed to carfilzomib resistance [14]. We first applied GSEA to examine gene sets from the canonical pathways (C2:CP) collection of MSigDB (1,330 gene sets). The most significantly enriched set of upregulated genes in the carfilzomib-resistant derivatives was the proteasome pathway (Kegg: hsa03050), with *PSMB5* encoding the β5 proteasome subunit targeted by carfilzomib as the top-ranked gene (normalized enrichment score, NES = 2.62, false discovery rate, FDR < 0.001; Figure S1A) [23]. The strength of the GSEA method is its utility in identifying modest changes in expression of groups of genes distributed across entire networks or pathways [14]. Real-time reverse transcription polymerase chain reaction (qRT-PCR) analysis validated the microarray expression data that *PSMB5* mRNA levels were only slightly increased (Table 1). Likewise, no marked increase was observed in mRNA for the immunoproteasome β5i/LMP7 subunit (encoded by *PSMB8*) that is also specifically targeted by carfilzomib [23]. Based on prior findings that bortezomib-resistant cell lines with 2- to 4-fold increased *PSMB5* mRNA levels retained sensitivity to carfilzomib [24], these results suggested that additional mechanisms may contribute to carfilzomib resistance in KMS-11/Cfz and KMS-34/Cfz cells.

**Table 1:** Gene expression changes associated with acquisition of carfilzomib resistance (KMS-11/Cfz and KMS-34/Cfz) and KLF4 overexpression (KMS-11/KLF4) in MM cells

Gene	Fold Change		
	KMS-11/Cfz	KMS-34/Cfz	KMS-11/KLF4
CCND1	0.06	0.17	1.12
CYP1A1	3.02	2.77	2.17
GLIPR1	1.19	1.22	0.70
HGF	0.76	0.45	0.65
HOXB7	1.33	1.69	1.38
ID1	3.61	2.29	1.63
IFIT3	1.25	1.87	0.73
IGF1	0.69	0.93	1.07
MAPT	1.21	0.94	1.13
NQO1	1.85	1.25	1.21
NQO2	1.09	1.05	0.75
P4HA2	0.70	0.67	0.68
PSMB5	1.09	1.11	0.83
SLAMF7	0.12	0.53	0.59
TLR4	1.43	3.56	1.13

Shown are fold changes relative to corresponding parental cells (mean values of three qRT-PCR experiments).

**Figure 1:**
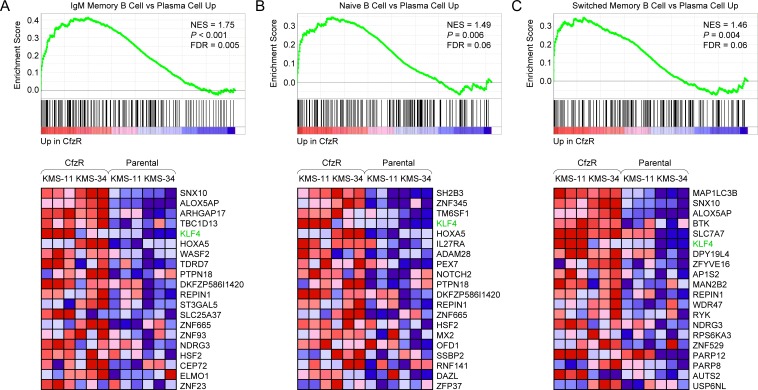
GSEA enrichment plots and heat maps of differentially expressed B lineage-related genes associated with acquisition of carfilzomib resistance in KMS-11 and KMS-34 cells **A.** Gene set: GSE13411_IGM_MEMORY_BCELL_VS_PLASMA_CELL_UP (M3249). **B.** Gene set: GSE13411_NAIVE_BCELL_VS_PLASMA_CELL_UP (M3243). **C.** Gene set: GSE13411_SWITCHED_MEMORY_BCELL_VS_PLASMA_CELL_UP (M3251). FDR, false discovery rate; NES, normalized enrichment score; CfzR, carfilzomib-resistant derivatives; KLF4 is indicated.

It was recently demonstrated that MM cells can acquire resistance to bortezomib via de-commitment to plasma cell differentiation [7]. Notably, among 1,910 gene sets in the immunologic signatures (C7) collection, three of those that were highly scored reflected a partial reversal of plasma cell maturation in the carfilzomib-resistant MM derivatives. The most significantly enriched gene set in KMS-11/Cfz and KMS-34/Cfz cells corresponded to genes with increased expression in IgM-memory B cells versus plasma cells (NES = 1.75, FDR = 0.005; Figure 1A). A set containing genes more highly expressed in naive B cells than in plasma cells (NES = 1.49, FDR = 0.06; Figure 1B) and one containing genes with higher levels of expression in Ig isotype-switched memory B cells relative to plasma cells (NES = 1.46, FDR = 0.06; Figure 1C) were also significantly enriched. We observed that *KLF4* was included within the leading edge subset of upregulated genes in all three gene sets, in line with its higher expression in naive and memory B cells than in plasma cells [25-27]. Furthermore, using GeneSpring analysis software, we found overrepresentation of KLF4 target genes previously characterized by genome-wide chromatin immunoprecipitation (ChIP) in embryonic stem cells by Orkin and colleagues [28] among the differentially expressed genes in KMS-11/Cfz (89 out of 887 genes, fold change, FC ≥ 1.4; *P* = 2.02 × 10^−3^) and KMS-34/Cfz (92 out of 888 genes, FC ≥ 1.5; *P* = 6.47 × 10^−4^) (Table S1), suggesting that upregulation of *KLF4* may contribute to carfilzomib resistance.

**Figure 2:**
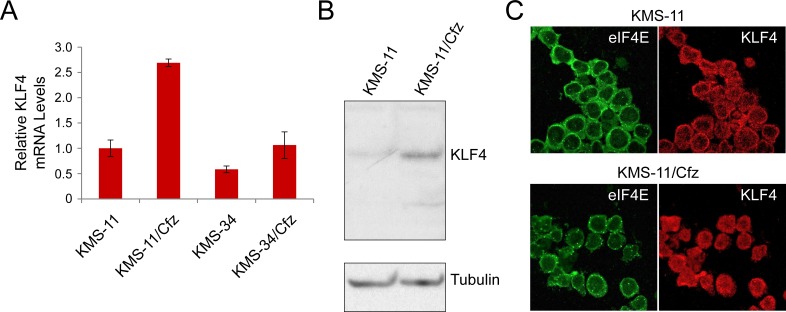
KLF4 expression in KMS-11 and carfilzomib-resistant KMS-11/Cfz cells **A.** Relative *KLF4* mRNA levels as determined by qRT-PCR. Also shown are *KLF4* mRNA levels in KMS-34 and KMS-34/Cfz cells relative to *KLF4* mRNA levels in KMS-11 cells. **B.** KLF4 protein levels were detected by western blotting with rabbit anti-KLF4 monoclonal antibodies against the carboxyl terminus (D1F2; Cell Signaling). Representative of four experiments. KLF4 signal in KMS-11 parental cells is less than in KMS-11/Cfz (*P* < 0.009). **C.** Cells were labeled with anti-KLF4 (Alexa Fluor 568, red) or anti-eIF4E (Alexa Fluor 488, green) antibodies, and immunofluorescence staining was analyzed by confocal laser scanning microscopy.

We confirmed increased expression of *KLF4* mRNA in carfilzomib-resistant MM cells by qRT-PCR analysis (Figure 2A), which was paralleled by a corresponding increase in KLF4 protein levels (~3.0 ± 0.7-fold, *n* = 4, *P* < 0.009 by paired Student's *t* test) detected by western blotting (Figure 2B). Consistent with its function as a transcriptional regulator and potential role in the carfilzomib-resistant phenotype [29, 30], immunofluorescence confocal microscopy revealed more prominent nuclear localization of KLF4 in the carfilzomib-resistant MM derivatives (Figure 2C).

We tested the hypothesis that KLF4 might contribute to the molecular phenotype of carfilzomib-resistant MM cells by short-term expression of a KLF4 cDNA in KMS-11 cells (denoted KMS-11/KLF4). Fourteen genes showing differential expression in KMS-11/Cfz (FC ≥ 1.4) and/or KMS-34/Cfz (FC ≥ 1.5) versus parental MM cells were selected, and their expression levels compared to those in KMS-11/KLF4 cells by qRT-PCR analysis (Table 1). A substantial degree of correlation was found between the expression changes associated with the introduction of exogenous *KLF4* and acquisition of carfilzomib resistance in KMS-11/Cfz (*r* = 0.77) and KMS-34/Cfz cells (*r* = 0.59) for the selected set of genes (Table 1). Of note, several of the genes that exhibited KLF4-induced changes in expression — *CYP1A1*, *NQO1*, *HOXB7* and *ID1* — were previously identified as direct KLF4 targets by genome-wide ChIP analysis [28]. Another of the genes, *SLAMF7* encodes CD319, a cell surface marker specifically upregulated at the plasma cell stage during B cell differentiation [27, 31]. We verified that *SLAMF7* mRNA levels were downregulated in the carfilzomib-resistant MM derivatives in accord with the less differentiated plasma cell phenotype revealed by GSEA analysis above. Moreover, *SLAMF7* mRNA levels were also reduced following ectopic expression of *KLF4* in KMS-11 cells. Together, the accumulated data supported the notion that carfilzomib-resistant MM cells had increased KLF4 transcriptional activity which was associated with the partial reversal of plasma cell maturation during acquisition of drug resistance.

### GSEA identifies altered expression of KLF4 target genes associated with autophagy and metabolic pathways in carfilzomib-resistant MM cells

**Figure 3:**
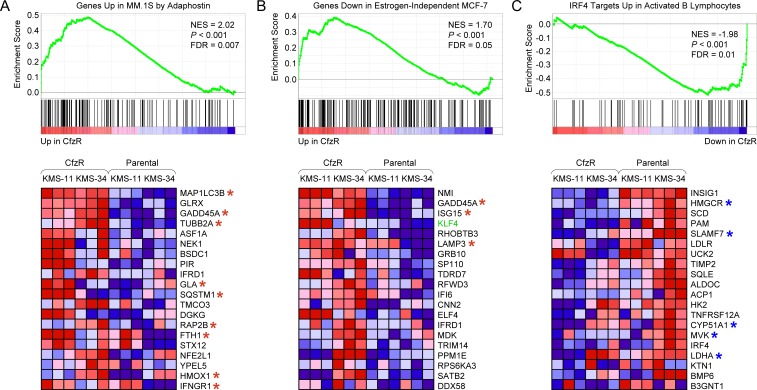
GSEA enrichment plots and heat maps of gene expression changes in sets of genes coregulated in response to chemical and genetic perturbations associated with acquisition of carfilzomib resistance in KMS-11 and KMS-34 cells **A.** Gene set: SHAFFER_IRF4_TARGETS_IN_ACTIVATED_B_LYMPHOCYTE (M11189). **B.** Gene set: PODAR_RESPONSE_TO_ADAPHOSTIN_UP (M16336). **C.** Gene set: LTE2_UP.V1_DN (M2721). Abbreviations are as in Figure 1. Selected autophagy-related genes are indicated with a red asterisk. KLF4-repressed genes are indicated with a blue asterisk.

We next used GSEA to examine gene sets from the C2:CGP chemical and genetic perturbations (3,395 gene sets) and C6 oncogenic signatures (189 gene sets) collections of MSigDB. A highly enriched C2:CGP gene set in KMS-11/Cfz and KMS-34/Cfz cells (NES = 2.02, FDR < 0.007; Figure 3A) represented molecular targets that were upregulated during inhibition of MM cell growth following treatment with adaphostin [32]. Bortezomib reportedly triggered similar effects, and a recent study identifying c-Abl as a regulator of proteasome homeostasis provides some insight into the cross-talk between the pathways involved [33]. Interestingly, included in the leading edge subset were several genes associated with autophagy (highlighted in Figure 3). Among them were *MAP1LC3B* encoding the autophagic effector protein microtubule-associated protein 1 light chain Cβ, and *SQSTM1* encoding the selective autophagy receptor sequestosome 1/p62 [34, 35]. During autophagy, a soluble form of LC3B (LC3B-I) is converted to a form (LC3B-II) that specifically associates with autophagosomes and is recognized by SQSTM1/p62 [36]. Also included was *GADD45A*, a growth arrest and DNA repair gene previously shown to be a KLF4-inducible gene in Hodgkin lymphoma cells and a stimulator of autophagy during skeletal muscle atrophy [37, 38]. Moreover, the most highly enriched gene set in the C6 collection (NES = 1.70, FDR = 0.05) corresponded to genes in MCF-7 breast cancer cells adapted for estrogen-independent growth in culture that were upregulated in the carfilzomib-resistant MM cells; *KLF4* and *GADD45A* were included in the leading edge subset (Figure 3B). A further association with autophagy-related processes was suggested by a recent study reporting that one of the genes in the leading edge subset, *LAMP3* encoding lysosome-associated membrane protein 3, is involved in resistance to the anti-estrogen tamoxifen in breast cancer cells by promoting prosurvival autophagy [39]. Another gene, *ISG15*, encoding an ubiquitin-like protein, was recently shown to interact with SQSTM1/p62, augmenting association with LC3B-II and facilitating autophagic degradation of aggresomes, in response to various types of cellular stress including proteasome inhibition [40]. Considered together, the genes represented in the leading edge subsets of the gene sets presented in Figure 3A, 3B suggested potential upregulation of autophagy pathways in the carfilzomib-resistant MM derivatives.

Conversely, among the downregulated sets of genes that were enriched in KMS-11/Cfz and KMS-34/Cfz cells, one of the most highly scored in the C2:CGP collection corresponded to genes belonging to an IRF4 regulatory network that are expressed at higher levels during B cell differentiation (NES = −1.98, FDR = 0.01; Figure 3C) [41]. *SLAMF7* was included in the leading edge subset of downregulated genes. We noted that many of the other downregulated genes in the leading edge subset are involved in lipid metabolism (e.g., *INSIG1*, *HMGCR*, *SCD*, *PAM*, *LDLR*, *SQLE*, *CYP51A1*, *MVK*), and complementary analysis of the C2:CP canonical pathways collection (1,330 gene sets) by GSEA indicated downregulation of genes involved in cholesterol biosynthesis (Reactome: REACT_9405.3) in the carfilzomib-resistant MM cells as being statistically significant (NES = −2.41, FDR = 0.001; Figure S1B). Consistent with the inverse correlation observed between KLF4 levels and expression levels of these genes, several were previously demonstrated to be negatively regulated by ectopic *KLF4* expression [42]. Examples included three genes involved in cholesterol biosynthesis: *HMGCR* encoding HMG-CoA reductase and *MVK* encoding mevalonate kinase, early enzymes in the mevalonate pathway; and *CYP51A1* encoding a cytochrome P450 family member involved in the conversion of lanosterol to cholesterol. Additionally, although no significant enrichment of glycolysis-related gene sets was revealed by GSEA, *LDHA*, which encodes a subunit of lactate dehydrogenase, a key enzyme in the glycolytic phenotype of cancer cells (known as the Warburg effect) [43] previously identified as a KLF4-repressed gene [44] was downregulated in the carfilzomib-resistant MM derivatives (Figure 3C). In view of increasing evidence that enhanced lipid catabolism and low glycolytic activity in slowly proliferating tumor cells is associated with autophagy induction and treatment resistance [45], the above observations raised the possibility that activation of prosurvival autophagy could be a mechanism adopted by the carfilzomib-resistant MM cells to counteract the deficiency of proteasome function and metabolic stress induced by carfilzomib treatment.

### Carfilzomib resistance is associated with prosurvival autophagy in MM cells

**Figure 4:**
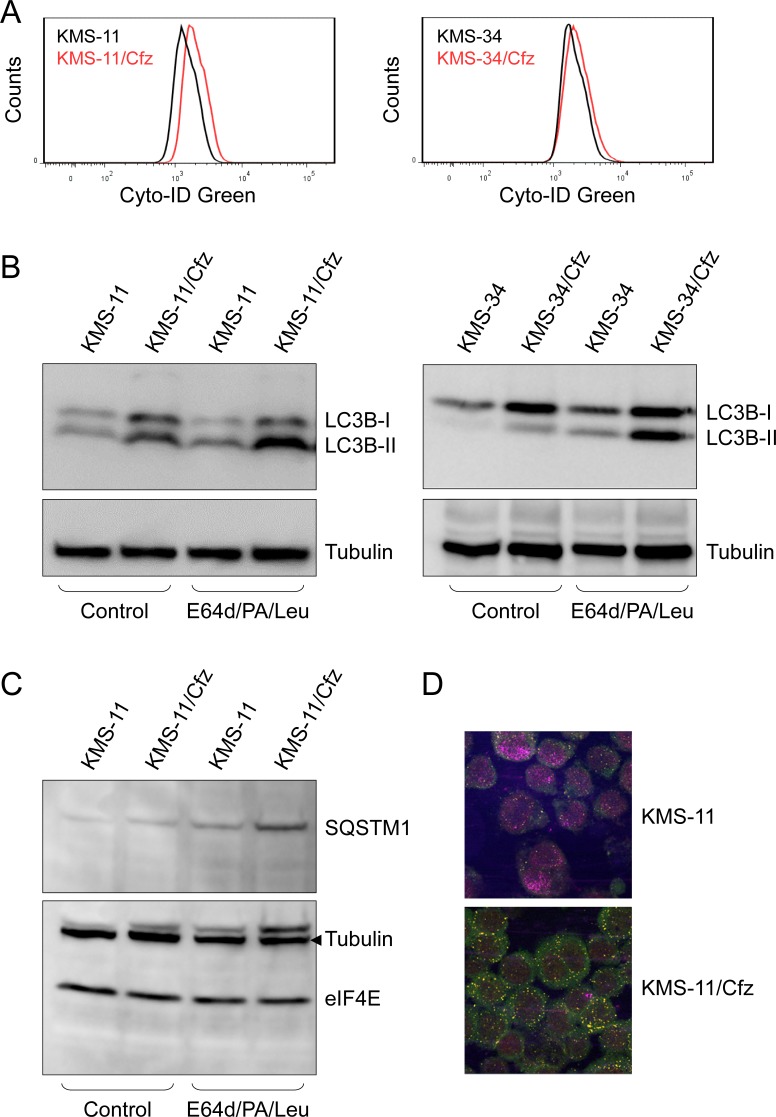
Autophagy is induced in carfilzomib-resistant KMS-11/Cfz and KMS-34/Cfz cells **A.** Fluorescence histograms of cells stained with the Cyto-ID Green autophagy detection reagent. Representative examples of three independent experiments are shown (*P* < 0.05, paired Student's *t* test). **B.**, **C.** To assess complete autophagic flux, cells were treated overnight with or without the lysosomal protease inhibitors E64d (10 μg/ml), pepstatin A (PA; 10 μg/ml), and leupeptin (Leu; 1 μg/ml) [52]. Cell lysates were probed with anti-LC3B **B.** or anti-SQSTM1 **C.**. **D.** Cells were labeled with anti-LC3B (Alexa Fluor 568, red) or anti-SQSTM1 (Alexa Fluor 488, green) antibodies, and immunofluorescence staining was analyzed by confocal laser scanning microscopy. Increased colocalization of the two factors in KMS-11/Cfz versus parental KMS-11 cells (yellow signals) correlated with acquisition of carfilzomib resistance (*P* < 0.003).

We first examined whether autophagic activity was increased in carfilzomib-resistant KMS-11/Cfz and KMS-34/Cfz cells by flow cytometry using the fluorescent dye Cyto-ID Green [46, 47]. Fluorescence signals reflect steady state levels of autophagosomes as a result of two offsetting processes: formation of autophagosomes and dissolution of autophagosomes upon lysosomal fusion. We found higher steady state levels of autophagosomes in resistant cells versus their parental counterparts in both MM cell line models (relative increase in autophagic activity based on Cyto-ID mean fluorescence intensity values = 16.6 ± 1.3, *P* < 0.05, paired Student's *t* test; see Materials and Methods for details) (Figure 4A).

Second, we investigated autophagic flux by measuring the processing of LC3B, an ubiquitin-like protein that is converted during autophagy to a lipidated form (LC3B-II) associated with autophagosome membranes [34]. To distinguish between synthesis, modification and degradation rates of LC3B, the ratio of the LC3B-II faster migrating form to the nonlipidated precursor (LC3B-I) is determined in the absence or presence of inhibitors of lysosome-mediated proteolysis [48]. Both carfilzomib-resistant MM derivatives exhibited higher levels of synthesis and modification of LC3B (as measured by LC3B-II/LC3B-I ratio) in comparison to parental cells in the presence of a mixture of the lysosomal protease inhibitors E64d, pepstatin A and leupeptin (Figure 4B). Increased levels of the ubiquitin cargo receptor and autophagy substrate SQSTM1/p62 in KMS-11/Cfz cells in the presence of the lysosomal protease inhibitors was also consistent with more active autophagy (Figure 4C).

**Figure 5:**
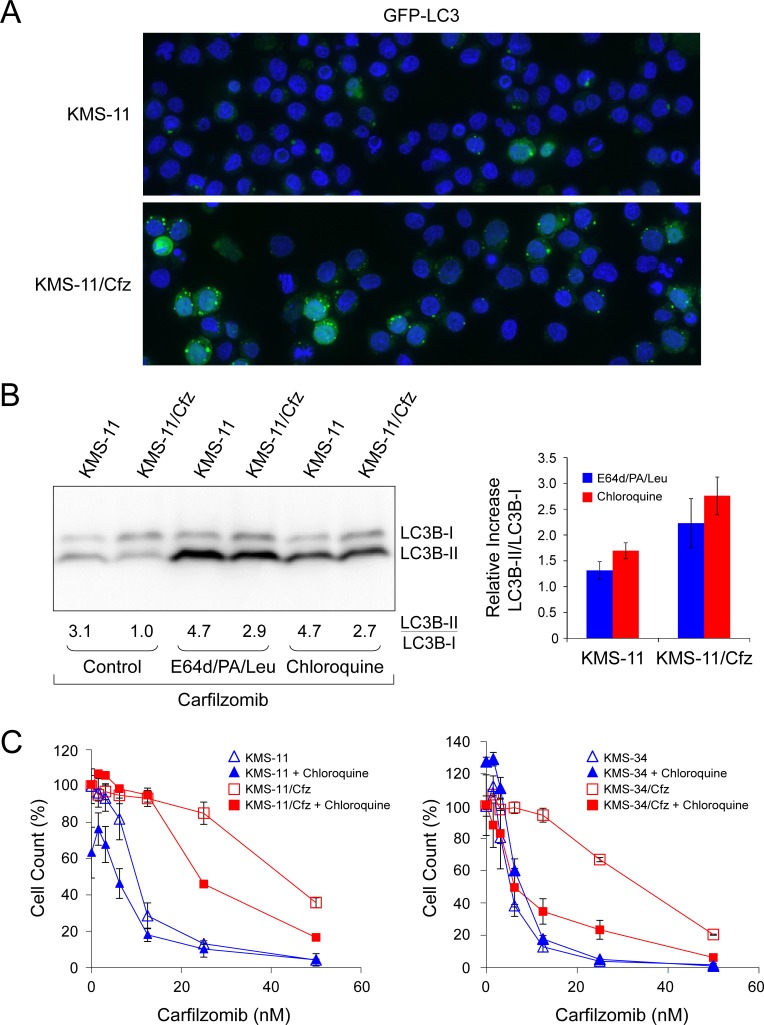
Prosurvival autophagy induction in carfilzomib-resistant MM cells is antagonized by lysosomal inhibitors and chloroquine **A.** Increased GFP-LC3 puncta formation in KMS-11/Cfz versus parental KMS-11 cells treated overnight with carfilzomib (50 nM). **B.** Increased stabilization of LC3B-II in KMS-11/Cfz versus parental KMS-11 cells upon autophagy inhibition. Representative western blot of LC3B levels of four independent experiments is shown (*P* < 0.0001). Cells were treated overnight with carfilzomib (50 nM) in the presence or absence of the lysosomal protease inhibitors E64d (10 μg/ml), pepstatin A (PA; 10 μg/ml), and leupeptin (Leu; 1 μg/ml) or chloroquine (10 μM). Densitometry was used to calculate the LC3B-II/LC3B-I ratios and the relative increases in the ratios upon autophagy inhibition with respect to carfilzomib only-treated controls are indicated in the graph on the right. **C.** Cells were treated with the indicated concentrations of carfilzomib for 72 hours in the presence or absence of chloroquine (10 μM). Cell viability was determined by alamarBlue assay. The data are presented as the mean ± S.D of three experiments. IC_50_ values were calculated by linear regression of the plots in the absence or presence of chloroquine: KMS-11/Cfz, 35 nM versus 22 nM; KMS-11, 12 nM versus 12 nM; KMS-34/Cfz, 35 nM versus 6 nM; KMS-34, 6 nM versus 6 nM.

Next, since SQSTM1/p62 is recruited into autophagosomes by lipidated LC3B-II [49], we assessed the subcellular localization of LC3B and SQSTM1/p62 proteins by immunofluorescence confocal microscopy. In both KMS-11 and KMS-34 cell line models increased colocalization of the two factors correlated with acquisition of carfilzomib resistance (*P* < 0.003; Figure 4D). To further characterize autophagic activity, we used the fluorescent autophagosome-specific reporter construct encoding a GFP-LC3 fusion protein [50], and examined GFP-LC3 signals by fluorescence confocal microscopy in KMS-11 and KMS-11/Cfz cells treated with or without carfilzomib. An increased number of GFP-LC3-II puncta was observed in KMS-11/Cfz cells (Figure 5A). To determine whether this was associated with increased autophagic flux, cells were treated with carfilzomib in the presence of lysosomal protease inhibitors and the levels of endogenous LC3B-I and LC3B-II measured by western blotting. As shown in Figure 5B, the degree of LC3B-II stabilization as reflected by the relative increase in the LC3B-II/LC3B-I ratio was greater in carfilzomib-resistant versus parental MM cells (*P* < 0.0001). Similar results were obtained when the cells were cotreated with chloroquine which, by increasing lysosomal pH, inhibits autophagic protein degradation and blocks autophagosome-lysosome fusion (Figure 5B) [51].

The above results are consistent with the view that increased autophagic flux was occurring in the carfilzomib-resistant MM cells. Inhibition of autophagy by chloroquine treatment was previously reported to enhance carfilzomib-induced cell death of head and neck squamous cell carcinoma cell lines [52]. To test whether concomitant inhibition of autophagy would sensitize KMS-11/Cfz and KMS-34/Cfz to carfilzomib, the cells were treated with carfilzomib in the presence or absence of chloroquine, and cell growth was measured by alamarBlue assay. Importantly, cotreatment with chloroquine diminished carfilzomib resistance in both KMS-11/Cfz and KMS-34/Cfz cell lines, indicating that prosurvival autophagy contributes to acquired drug resistance in these MM cell line models (Figure 5C).

### KLF4 regulates the autophagy receptor gene SQSTM1

**Figure 6:**
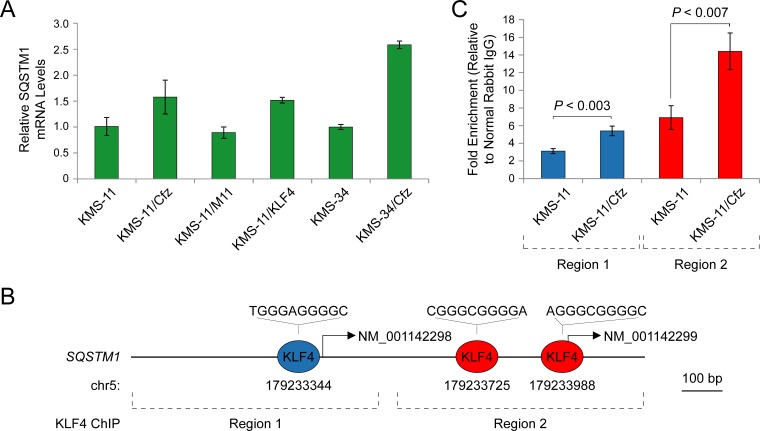
SQSTM1 is a direct target of KLF4 upregulated in carfilzomib-resistant KMS-11/Cfz and KMS-34/Cfz cells **A.** Expression of *SQSTM1* determined by qRT-PCR. KMS-11/KLF4, KMS-11 cells expressing a KLF4 cDNA. KMS-11/M11, KMS-11 cells transfected with a control vector. **B.** Evolutionarily conserved KLF4-binding motifs in the *SQSTM1* promoter regions upstream of the NM_001142298 and NM_001142299 transcription start sites (Figure S2; see also Table S3 of Zaret and colleagues [54]). **C.** Increased binding of KLF4 to the *SQSTM1* promoter regions indicated in **B.** in KMS-11/Cfz versus parental KMS-11 cells as determined by ChIP-qPCR.

The SQSTM1/p62 protein is of particular interest given its role as an adaptor for both proteasomal and autophagic degradation of ubiquitinated proteins [20, 21]. As shown in Figure 6A, we confirmed that *SQSTM1* mRNA levels were higher in the carfilzomib-resistant MM derivatives and we demonstrated that the levels increased upon ectopic expression of *KLF4* in KMS-11 cells by qRT-PCR analysis. A review of the literature revealed that *SQSTM1* was included in a list of direct KLF4 binding targets detected by genome-wide ChIP analysis [53]. We therefore analyzed the genomic regions upstream of the *SQSTM1* transcription start sites, and identified evolutionally conserved KLF4-binding motifs (Figure 6B; Figure S2). We performed qPCR-based ChIP analysis in KMS-11 and KMS-11/Cfz cells and confirmed binding to these regions by endogenous KLF4 (Figure 6C). In these experiments, the *KLF4* promoter region was included as a positive control [55], whereas *GATA6* genomic regions were used as negative controls [28, 53]. These observations support the proposal that KLF4 contributes to carfilzomib resistance by upregulating *SQSTM1* expression in these MM cell line models.

To further investigate the potential relevance of a KLF4-associated autophagy component in MM, we identified *KLF4* profile neighbors across 304 MM patient samples in the Multiple Myeloma Research Consortium reference collection dataset (GEO accession number GSE26760). In agreement with earlier work [17, 18], the two genes dysregulated by the t(4;14) translocation, *WHSC1* and *FGFR3* [56], were among the top KLF4 neighbors (1,470 out of 54,675 total on the array). Moreover, the set was enriched for genes associated with the annotation term “autophagy” in the NCBI Gene database (*P* = 2.34 × 10^−4^). Importantly, the list included *SQSTM1* (Table S2). These results, demonstrating similarity in expression pattern and shared function [57], suggest that contribution to prosurvival autophagy is a clinically relevant aspect of *KLF4* expression as regards MM pathobiology.

### Elevated expression of KLF4 and SQSTM1 is prognostic of poor survival in a subgroup of WHSC1-positive MM patients

**Figure 7:**
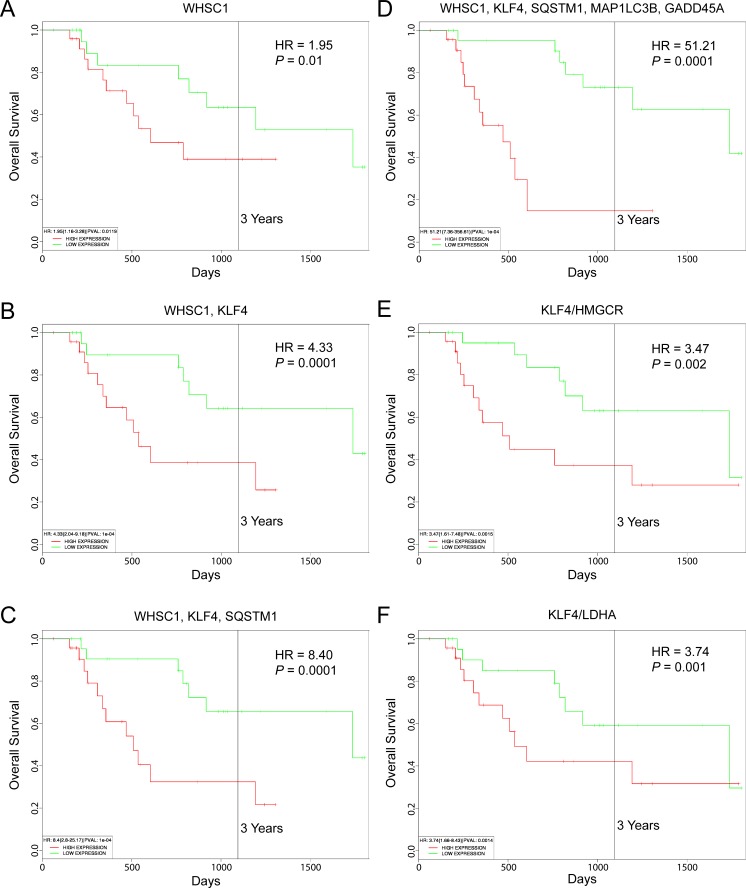
Prognostic value of WHSC1, KLF4 and SQSTM1 expression in MM patient survival outcomes Kaplan-Meier survival plots of 47 MM patients in a high-risk subgroup associated with refractory/relapsed disease (GEO accession number GSE2658) created using PROGgeneV2. **A.** *WHSC1* expression. **B.** Coexpression of *WHSC1* and *KLF4*. **C.** Coexpression of *WHSC1*, *KLF4* and *SQSTM1*. **D.** Coexpression of *WHSC1*, *KLF4*, *SQSTM1*, *MAP1LC3B*, and *SQSTM1*. **E.** Ratio of *KLF4* to *HMGCR* expression. **F.** Ratio of *KLF4* to *LDHA* expression. Median gene expression values were used as bifurcation points. HR, hazard ratio determined by Cox proportional hazards model.

Shaughnessy and colleagues defined a high-risk subgroup of MM patients by gene expression profiling (designated PR) based on expression of certain cell cycle and proliferation-associated genes [58]. The PR subgroup signature was present in approximately 18% of newly diagnosed MM and increased during disease progression to 45% of relapsed cases; strikingly, many of the *WHSC1*-defined t(4;14)-positive samples were found to cluster in this subgroup [58]. We used the recently published PROGgeneV2 prognostic biomarker identification tool [59] to study the implications of *WHSC1*, *KLF4* and *SQSTM1* gene expression on overall survival of 47 MM patients in the PR subgroup (GEO accession number GSE2658). Using median gene expression values as bifurcation points, Cox proportional hazards analyses showed that elevated expression of these genes was associated with inferior 3-year survival outcomes (Figure 7). Kaplan-Meier plots indicated significant segregation in survival outcomes for patients with high versus low *WHSC1* expression (hazard ratio, HR = 1.95; 95% confidence interval/CI, 1.16-3.28; *P* = 0.01), with increasingly worse prognosis when high level coexpression of *KLF4* (HR = 4.33; 95% CI, 2.04-9.18; *P* = 1.0 − 10^−4^) (Figure 7B), and *KLF4* plus *SQSTM1* (HR = 8.40; 95% CI, 2.80-25.17; *P* = 1.0 × 10^−4^) (Figure 7C) were also considered. When high levels of coexpression of the autophagy-associated genes *MAP1LC3B* and *GADD45A* were also taken into account, significantly inferior overall survival outcomes were observed (HR = 51.21; 95% CI, 7.36-356.61; *P* = 1.0 × 10^−4^) (Figure 7D). Moreover, reduced tumor cell metabolism (e.g., as exemplified by low *HMGCR* or *LDHA* expression) was also associated with adverse outcomes, with high expression ratios of *KLF4* to *HMGCR* (HR = 3.47; 95% CI, 1.61-7.48; *P* = 0.02) (Figure 7E) and *KLF4* to *LDHA* (HR = 3.74; 95% CI, 1.66-8.43; *P* = 0.001) (Figure 7F) prognostic of poor 3-year survival rates.

## DISCUSSION

The molecular mechanisms associated with the development of clinical resistance to proteasome inhibitors remain to be fully elucidated [4-8]. Toward this end, we have endeavored to establish clinically-relevant carfilzomib-resistant MM cell lines [60]. In a previously published study, we found that upregulation of the *ABCB1*-encoded multidrug resistance efflux transporter P-glycoprotein contributed to increased carfilzomib resistance in KMS-34 MM cells cultured in low concentrations (6 nM) of the drug [13]. Notably, our analysis of microarray data acquired for a MM patient treated with carfilzomib revealed increased *ABCB1* expression during disease progression [61], suggesting that the mechanism could be relevant to carfilzomib resistance observed in the clinic [62]. To further elaborate mechanisms of carfilzomib resistance in MM, we adapted KMS-34/Cfz to growth in 12 nM carfilzomib and likewise established KMS-11/Cfz cells resistant to 12 nM carfilzomib by exposure to stepwise increasing concentrations of the drug. Unlike KMS-34/Cfz, KMS-11/Cfz did not exhibit increased expression of *ABCB1*. Instead, we found that a common mechanism of carfilzomib resistance was elevated levels of prosurvival autophagy, and that the pluripotency-associated transcription factor KLF4 [15] contributed to the carfilzomib-resistant phenotype. Our findings are in accord with those of two publications that appeared during revision of this manuscript reporting that KLF4 participates in autophagic pathways activated during stress responses in other settings [63, 64].

Substrates destined for selective autophagic destruction are recognized by receptor proteins which anchor the material to the expanding phagophore membrane via co-binding to lipidated LC3B-II [35, 36]. In recent years, the ubiquitin-proteasome and autophagy-lysosome systems, once considered independent processes for protein degradation, are now regarded as being interconnected. Impairment of either pathway has been reported to impact the other. Ubiquitination of proteins targeted for destruction serves as the mechanism of crosstalk and ubiquitin-binding cargo protein SQSTM1/p62 is a critical link [20, 21]. By upregulating SQSTM1/p62 levels in carfilzomib-resistant MM cells, KLF4 is postulated to contribute to prosurvival autophagy by facilitating delivery of aggregated substrates to autophagosomes via LC3B-II for subsequent destruction [65]. The relevance of this process in the MM therapeutic response to proteasome inhibition is supported by the recent finding that short hairpin RNA knockdown of *SQSTM1* mRNA resulted in failure of autophagosomes to trap ubiquitinated cargo in MM cells, converting prosurvival autophagy to apoptosis [66].

*KLF4* expression is consistently associated with MM patients carrying the t(4;14) translocation [17, 18]. Two genes are aberrantly expressed as a consequence of the translocation: *WHSC1*, encoding a histone methyltransferase (also referred to as *MMSET* or *NSD2*), and *FGFR3* encoding a transmembrane tyrosine kinase [56]. Gain- and loss-of function studies in MM cell lines have demonstrated that WHSC1 histone methyltransferase activity is involved in the epigenetic upregulation of *KLF4* [67, 68]. Notably, there is highly significant overlap (*P* < 1 × 10^−33^) between the WHSC1 target genes identified and the differentially expressed genes in KMS-11/Cfz (86 out of 887; FC ≥ 1.4) and KMS-34/Cfz (69 out of 888 genes, FC ≥ 1.5). Therefore, inhibition of WHSC1 enzymatic function or its ability to interact with chromatin may represent a promising future combination therapy for this subgroup of MM patients [67, 68].

Although the upstream pathways remain undefined, it is worth noting that induction of *KLF4* transcription was previously observed in endothelial cells treated with bortezomib or epoxomicin (the natural epoxyketone from which carfilzomib was derived) [69]. Increased levels of KLF4 have also been observed following inhibition of translation by depletion of the translation initiation factor eIF4GI [70]. As eIF4GI depletion was reported to phenocopy mTOR inhibition and promote autophagy [70], and inhibition of mTOR activity by rapamycin induced *KLF4* expression in other cells [71], mechanisms linking protein turnover and protein synthesis are implicated in *KLF4* upregulation in the context of acquired proteasome inhibitor resistance [72].

Overexpression of *KLF4* in MM cells was previously reported to result in cell cycle arrest [19]. We also observed that very high levels of exogenous *KLF4* expression reduced proliferation rates in KMS-11 and KMS-34 cells, with diminished transgene expression in cell populations that exhibited a growth advantage after 1 month of culture (Figure S3). In this regard, KLF4 has been reported to exert growth suppressive effects in B-cell non-Hodgkin lymphoma [37], yet a recent study found that high nuclear expression of KLF4 in Burkitt pediatric lymphoma was indicative of inferior overall survival [73]. Thus, as in other systems [16], KLF4 regulation of B cell proliferation is complex and likely dependent on multiple factors. Along these lines, *KLF4* was originally characterized as an epithelial oncogene by Rupert's group imparting a slow growth phenotype to the transformed cells [74]. In follow-up studies of breast cancer cases, these investigators found that small primary tumors having preferential nuclear localization of KLF4 correlated with an increased risk of death [30]. The relative proliferative activity of *WHSC1*-positive *KLF4*-expressing MM cells with respect to other MM samples in the PR subgroup defined by Shaughnessy and colleagues [58] is not known. However, the fact that the hazard ratio increased significantly for these MM patients upon consideration of autophagy-associated gene expression levels (Figure 7D), implicates activation of autophagy as a contributing factor to relapsed/refractory disease.

Beyond its function as a direct cell cycle regulator, KLF4 exerts inhibitory effects on metabolic pathways and macromolecular synthesis [75], which include repression of genes encoding key enzymes involved in glycolysis and cholesterol biosynthesis (i.e., *HMGCR*, *HMGCS1*, *MVK*, *LDHA*) [42, 44]. Recent studies have shown that autophagy plays important roles in glucose homeostasis and lipid metabolism [76, 77], and the inverse relationship between autophagy and cell growth is becoming increasingly appreciated [78]. Although the KLF4 metabolic target genes in Figure 3C, including *HMGCR* and *LDHA* were only modestly downregulated upon acquisition of carfilzomib resistance, inhibition of HMGCR or LDHA enzymatic activity has resulted in autophagy induction in cancer cells [79, 80]. Furthermore, high gene expression ratios of *KLF4* to *HMGCR* and *KLF4* to *LDHA* were associated with adverse outcomes of certain MM patients (Figure 7E, 7F). Collectively, these results may raise a cautionary note regarding potential anti-MM therapeutic strategies that target these metabolic enzymes [81, 82].

*KLF4* levels decrease during B cell activation and differentiation into plasma cells [25-27]. Increased expression of *KLF4* in the carfilzomib-resistant KMS-11/Cfz and KMS-34/Cfz cells was indicative of “dedifferentiation” to an earlier maturation stage (Figure 1). Negative regulation of *SLAMF7* encoding plasma cell-specific CD319 [27, 31] by ectopic *KLF4* expression in KMS-11 cells (Table 1) suggests a potential role in the process. That this MM cell line phenomenon is biologically relevant is supported by work from Yaccoby who first described the ability of primary MM cells to dedifferentiate into an immature plasmablastic phenotype upon long-term co-culture on osteoclasts [83]. More recently, Karadimitris and colleagues reported epigenetic plasticity in MM patient samples and xenograft assays, where clinical drug resistance was attributed to bidirectional transitions between MM plasma cells and more quiescent pre-plasma cells [84]. Our results also complement the recent findings of Tiedemann and colleagues implicating less mature pre-plasma cells as contributing to therapeutic proteasome inhibitor resistance in MM [7]. Considered from this perspective, it is notable in view of its activity as a reprogramming factor that KLF4 target genes in embryonic stem cells were enriched in the differentially expressed genes in KMS-11/Cfz and KMS-34/Cfz cells [28]. These results suggest potential parallels with putative cancer stem-like cells [13, 85]. In line with this notion, it has been reported that overexpression of *KLF4* in breast cancer cells led to an increase of the cancer stem cell-like population [86]. In that work, *KLF4*-mediated maintenance of stem cell-like characteristics was accompanied by increased cell migration and invasion of the malignant cells. Our future studies will further examine the mechanistic ramifications of *KLF4* expression as regards relapse and treatment resistance in MM, and the utility of the KMS-11/Cfz and KMS-34/Cfz MM models for the development of novel autophagy-targeting combination therapies.

## MATERIALS AND METHODS

### Cell lines, plasmids and transfections

KMS-11 and KMS-34 MM cells were a kind gift from Dr. P. Leif Bergsagel (Mayo Clinic, Scottsdale, AZ) [87]. Cells were cultured in RPMI 1640 with GlutaMAX (Life Technologies) supplemented with 10% fetal bovine serum (Cambrex BioScience), 100 U/ml penicillin and 100 μg/ml streptomycin. The KLF4 expression vector containing a KLF4 cDNA (Accession No. BC030811.1) under the control of a cytomegalovirus promoter in the pReceiver-M11 backbone was from GeneCopoeia (Cat. No. EX-Z0482-M11). The pBABEpuro GFP-LC3 autophagy reporter plasmid was from Addgene (Plasmid No. 22405) [86]. Transfections were performed using the Amaxa nucleofector system with solution V and program X-001 settings (Lonza). Transfected cell lines were selected in 0.5 mg/ml G418 (pReceiver-M11 KLF4) or 0.5 μg/ml puromycin (pBABEpuro GFP-LC3). GFP-LC3-expressing cells were sorted on a FACSAria instrument equipped with FACSDiva software (BD Biosciences).

### Antibodies and reagents

The following antibodies were used: anti-KLF4 (D1F2) rabbit mAb (Cell Signaling Technology, Cat. No. 12173); anti-α-tubulin mouse mAb (DM1A) (EMD Millipore Corporation, Cat. No. CP06); anti-p62/SQSTM1 (Clone 3) mouse mAb (BD Transduction Laboratories, Cat. No. 610832); anti-LC3B affinity isolated rabbit polyclonal antibody (Sigma-Aldrich, Cat. No. L7543); and anti-eIF4E (P-2) mouse mAb (Santa Cruz Biotechnology, Cat. No. sc-9976). Carfilzomib was obtained from Active Biochem (Cat. No. A-1098), chloroquine was purchased from Selleck Chemicals (Cat. No. S4157), and the lysosomal inhibitors E-64d (Cat. No. E8640), leupeptin (Cat. No. 103476-89-7) and pepstatin A (Cat. No. 77170) were from Sigma-Aldrich.

### Microarray gene expression analysis and quantitative real-time qRT-PCR validation

Total RNA was isolated with the miRNeasy mini kit (Qiagen, Cat. No. 217004). Microarray gene expression analysis of triplicate samples was carried out by Expression Analysis, Inc. (Durham, NC) using Affymetrix GeneChip Human Genome U133 Plus 2.0 arrays. The data have been deposited in GEO (http://www.ncbi.nlm.nih.gov/geo/) under accession number GSE69078. Reverse transcription was performed with the SuperScript VILO cDNA synthesis kit (Life Technologies, Catalog No. 11754-250). Real-time qRT-PCR was performed using the Power SYBR Green reagent (Life Technologies, Cat No. 4368708) on an ABI Prism 7000 Sequence Detection System (Life Technologies) as previously described [13]. Primers synthesized by Sigma-Aldrich included: CCND1, forward, GCTCACGCTTACCTCAACCA, reverse, GACAGACAAAGCGTCCCTCA; CYP1A1, forward, GCTGCCTTCTGGCCTTGTAA, reverse, TGCCTGGATATGTGCACTCC; GLIPR1, forward, GACTGCGTTCGAATCCATAACA, reverse, GCTGGGTCCCAAGTCATGTA; HGF, forward, GACGCAGCTACAAGGGAACA, reverse, GGCAAAAAGCTGTGTTCGTG; HOXB7, forward, TGCAGTTTTGTAAGCCCTCT, reverse, GCAACCACAGGGTTAGTCCA; ID1, forward, CCAGCACGTCATCGACTACA, reverse, GGGGGTTCCAACTTCGGATT; IFIT3, forward, CTGGGTGGAAACCTCTTCAGC, reverse, GACCTCACTCATGATGGCTGTTTC; IGF1, forward, TGCAGGAGGGACTCTGAAAC, reverse, GCTGCGTGATATTTGAAAGGT; KLF4, forward, TCCATTACCAAGAGCTCATGCC, reverse, CGCGTAATCACAAGTGTGGG; MAPT, forward, AGCTTGTAGCTGCCAACCTC, reverse, TTTCCAAGGGGGTGTGTTCC; NQO1, forward, TAGCATTGGGCACACTCCAG, reverse, CCAGGCGTTTCTTCCATCCT; P4HA2, forward, GACACTTCCCTCTGTGACCA, reverse, TCATGTGCCCAATAGAGGTG; PSMB5, forward, CAGTACAAAGGCATGGGGCT, reverse, TCAGACACAGGGCCTCTCTTA; SLAMF7, forward, AGTCTGGCACGTAAGATGAACA, reverse, TCAAAAGCAGCCATTCCCCT; SQSTM1, forward, CTCCGCGTTCGCTACAAAAG, reverse, CAGAAGGTAGGCCTTCACGG; and TLR4, forward, TGCCGTTTTATCACGGAGGT, reverse, GGGAGGTTGTCGGGGATTTT.

### Chromatin immunoprecipitation (ChIP)

ChIP was performed on the *SQSTM1* promoter regions with 20 μg total chromatin and 5 μg of anti-KLF4 (D1F2) rabbit mAb using the SimpleChIP Enzymatic Chromatin IP Kit (Magnetic Beads) (Cell Signaling, Cat. No. 9003), and 4% of the precipitated material was used per qPCR reaction. *GATA6* was included as a non-target promoter. Background ChIP levels were obtained using 5 μg of normal rabbit IgG (Cat. No. 2729). Primers used were: SQSTM1 promoter region 1, forward, AGCTTTGTGCCCTGTACTCA, reverse, TGCAGTGAGCCTGATACCTG; SQSTM1 promoter region 2, ACCTCTGTGACCTTGGGTCT, reverse, GCTGTCCCGACGCTGAG; GATA6 promoter region 1, forward, TGTTGAACTGTGCAGCTTTTCT, reverse, TAATGATGCACACACAACCTGA; GATA6 promoter region 2, forward, ATTCCCAGCAGGCTTATTGTAAA, reverse, CAGAAGCAGACAACCACGATAG.

### Confocal microscopy

Cells (2.5 × 10^5^) were centrifuged onto a microscope slide at 1,000 rpm for 5 minutes using a Shandon Cytospin 4 instrument. The cells were then immediately fixed in 3.7% formaldehyde for 5 minutes at room temperature and permeabilized with 0.5% Triton X-100 in phosphate-buffered saline (PBS) for 15 minutes at room temperature. Following permeabilization, the cells were rinsed with PBS and blocked in PBS containing 10% goat serum and 0.01% Triton X-100 for 1 hour at room temperature. The cells were then incubated with anti-KLF4 and anti-eIF4E antibodies diluted to final concentrations of 0.4 μg/ml, in PBS containing 1% goat and 0.01% Triton X-100 for 1 hour at room temperature. The cells were rinsed with PBS and then incubated with Alexa Fluor 568-conjugated goat anti-rabbit and Alexa Fluor 488-conjugated goat anti-mouse secondary antibodies (Life Technologies) diluted 1:500 in PBS containing 1% goat serum and 0.01% Triton X-100 for 1 hour at room temperature. The cells were rinsed with PBS and mounted with Fluoromount G (Electron Microscopy Sciences). Cells expressing the GFP-LC3 reporter were fixed in 1% methanol free paraformaldehyde (Electron Microscopy Sciences) in PBS overnight at 4°C then centrifuged onto a microscope slide and mounted with Fluoromount G. Imaging analysis was performed on a Cell Observer SD spinning disk confocal system equipped with Zen software (Carl Zeiss Microscopy).

### Cytotoxicity assay

Cells were treated with carfilzomib and chloroquine at the indicated concentrations and cell growth was measured using the alamarBlue cell viability and proliferation reagent (Life Technologies) as previously described [13].

### Autophagy detection

Autophagy was measured with the Cyto-ID autophagy detection kit (Enzo, Cat. No. ENZ-51031-K200) using a FACSAria instrument, and data were analyzed with FlowJo Mac v10.0.2 (Tree Star). Autophagy activity factor (AAF) values were calculated using the following equation: AAF = 100 × (MFI _carfilzomib-resistant_ − MFI _parental_) / MFI _carfilzomib resistant_. MFI is mean fluorescence intensity, and AAF expresses the level of autophagy in live cells as the difference between the amount of Cyto-ID Green autophagy dye within cells [46]. GFP-LC3 puncta were detected by confocal microscopy as described above.

## SUPPLEMENTARY MATERIAL FIGURES AND TABLES


