# Supplementary material for: KLF4-SQSTM1/p62-associated prosurvival autophagy contributes to carfilzomib resistance in multiple myeloma models
Source: Oncotarget. 2015 Jun 19;6(17):14814–31. doi: 10.18632/oncotarget.4530 (PMC4558117; doi:10.18632/oncotarget.4530)
Supplement: Supplementary file 1 [file oncotarget-06-14814-s001.pdf]

KLF4-SQSTM1/p62-associated prosurvival autophagy contributes to carfilzomib resistance in multiple myeloma models

Supplementary Material

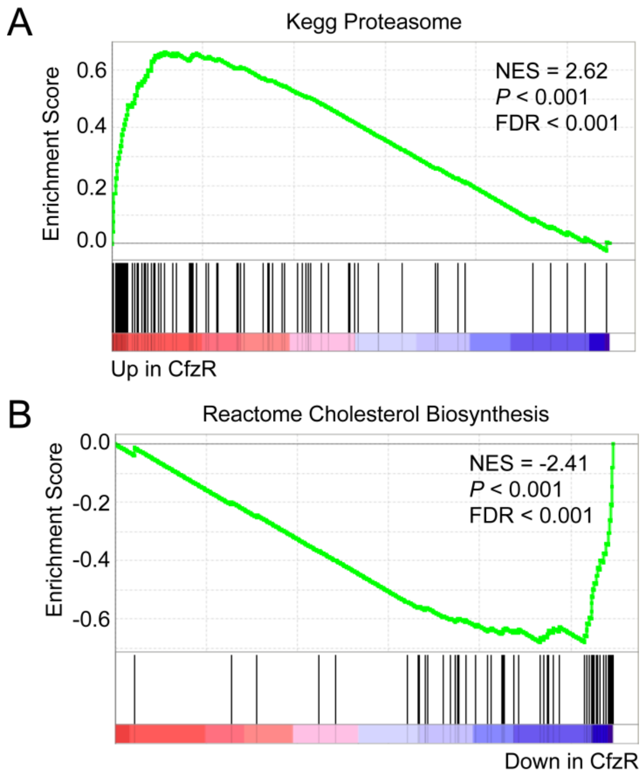

Supplementary Figure S1: GSEA enrichment plots associated with acquisition of carfilzomib resistance in KMS-11/Cfz and KMS-34/Cfz cells. (A) Gene set: KEGG_PROTEASOME. **(B)** Gene set: REACTOME_CHOLESTEROL_BIOSYNTHESIS. FDR, false discovery rate; NES, normalized enrichment score; CfzR, carfilzomib-resistant derivatives.

A

KLF4 motif (M01588)	(t/a)GGG(c/t/a)G(t/g)GG(c/t)					
chr5: 179233344-179233353	T	GGG	A	G G	GG	C
chr5: 179233725-179233734	C	GGG	C	G G	GG	A
chr5: 179233988-179233997	A	GGG	C	G G	GG	C

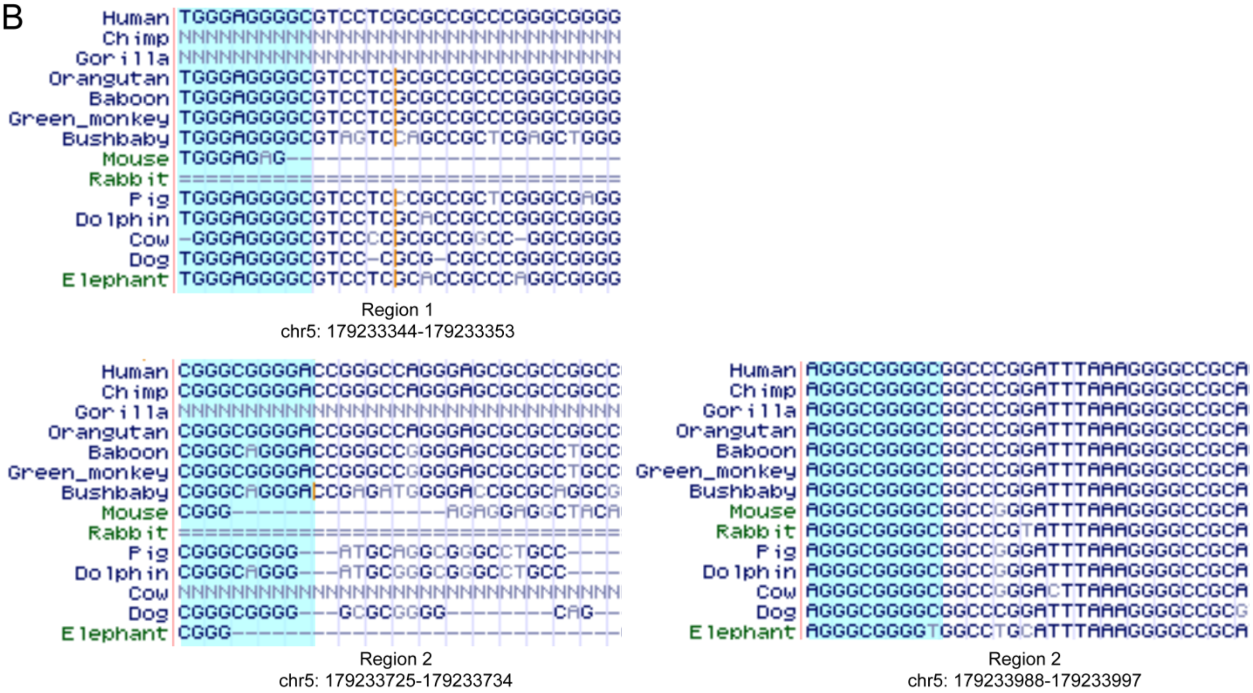

Supplemenatry Figure S2: Evolutionarily conserved KLF4-binding sites in the *SQSTM1* promoter regions. (A) Positions of KLF4 consensus motifs (TRANSFAC Motif ID, M01588) in the human *SQSTM1* gene. **(B)** Alignment of the sequences in (A) across species (blue highlighted regions). Note that the KLF4 motif (AGGGCGGGGC) in region 2, chr5: 179233988-179233997, of the *SQSTM1* promoter shows almost perfect conservation across all 14 species.

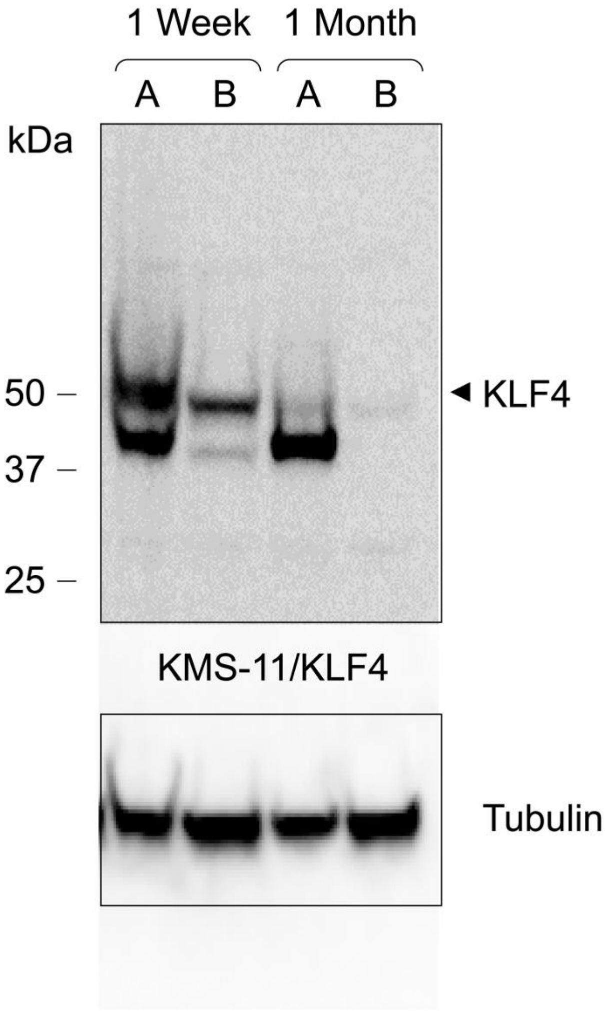

Supplementary Figure S3: Overexpression of exogenous KLF4 is selected against during growth of KMS-11/KLF4 cells in culture. KLF4 protein levels were detected by western blotting with rabbit anti-KLF4 monoclonal antibodies against the carboxyl terminus (D1F2; Cell Signaling). Cell populations that grew out after 1 month of culture had diminished levels of exogenous KLF4 expression. A and B denote two independent transfections.

Supplementary Table S1. Differentially expressed genes in KMS-11/Cfz versus KMS-11

Probe Set ID	Symbol	Name	Change	FC > 1.36
209459_s_at	ABAT	4-aminobutyrate aminotransferase	up	1.38
209460_at	ABAT	4-aminobutyrate aminotransferase	up	1.42
203192_at	ABCB6	ATP-binding cassette, sub-family B (MDR/TAP), member 6	up	1.49
205566_at	ABHD2	abhydrolase domain containing 2	down	1.55
228490_at	ABHD2	abhydrolase domain containing 2	down	1.45
200710_at	ACADVL	acyl-CoA dehydrogenase, very long chain	up	1.73
204565_at	ACOT13	acyl-CoA thioesterase 13	up	1.40
205942_s_at	ACSM3	acyl-CoA synthetase medium-chain family member 3	down	1.37
210377_at	ACSM3	acyl-CoA synthetase medium-chain family member 3	down	1.50
216705_s_at	ADA	adenosine deaminase	down	1.40
202603_at	ADAM10	ADAM metallopeptidase domain 10	down	1.39
202604_x_at	ADAM10	ADAM metallopeptidase domain 10	down	1.62
214895_s_at	ADAM10	ADAM metallopeptidase domain 10	down	1.49
217007_s_at	ADAM15	ADAM metallopeptidase domain 15	up	1.38
205997_at	ADAM28	ADAM metallopeptidase domain 28	up	1.55
208268_at	ADAM28	ADAM metallopeptidase domain 28	up	1.64
203741_s_at	ADCY7	adenylate cyclase 7	up	1.52
224480_s_at	AGPAT9	1-acylglycerol-3-phosphate O-acyltransferase 9	up	1.48
224461_s_at	AIFM2	apoptosis-inducing factor, mitochondrion-associated, 2	up	1.49
228445_at	AIFM2	apoptosis-inducing factor, mitochondrion-associated, 2	up	1.54
206513_at	AIM2	absent in melanoma 2	up	1.69
201951_at	ALCAM	activated leukocyte cell adhesion molecule	down	1.38
201952_at	ALCAM	activated leukocyte cell adhesion molecule	down	1.45
231202_at	ALDH1L2	aldehyde dehydrogenase 1 family, member L2	down	3.13
202022_at	ALDOC	aldolase C, fructose-bisphosphate	down	1.50
225969_at	ALKBH6	alkB, alkylation repair homolog 6 (E. coli)	up	1.38
204446_s_at	ALOX5	arachidonate 5-lipoxygenase	up	1.66
209425_at	AMACR	alpha-methylacyl-CoA racemase	down	1.42
229497_at	ANKDD1A	ankyrin repeat and death domain containing 1A	down	1.89
238332_at	ANKRD29	ankyrin repeat domain 29	up	1.42
1556183_at	ANKRD36BP2	ankyrin repeat domain 36B pseudogene 2	down	1.59
227337_at	ANKRD37	ankyrin repeat domain 37	down	1.58
219496_at	ANKRD57	ankyrin repeat domain 57	down	1.39
227034_at	ANKRD57	ankyrin repeat domain 57	down	1.55
201590_x_at	ANXA2	annexin A2	up	1.42
210427_x_at	ANXA2	annexin A2	up	1.46
213503_x_at	ANXA2	annexin A2	up	1.49
208816_x_at	ANXA2P2	annexin A2 pseudogene 2	up	1.45
204894_s_at	AOC3	amine oxidase, copper containing 3 (vascular adhesion protein 1)	down	1.58
237159_x_at	AP1S3	adaptor-related protein complex 1, sigma 3 subunit	down	1.37
1555731_a_at	AP1S3	adaptor-related protein complex 1, sigma 3 subunit	down	1.37
209546_s_at	APOL1	apolipoprotein L, 1	up	1.61
221087_s_at	APOL3	apolipoprotein L, 3	down	1.71
225173_at	ARHGAP18	Rho GTPase activating protein 18	down	1.39
58780_s_at	ARHGEF40	Rho guanine nucleotide exchange factor (GEF) 40	up	1.37
209135_at	ASPH	aspartate beta-hydroxylase	up	1.41
233536_at	ASXL3	additional sex combs like 3 (Drosophila)	up	1.65
204998_s_at	ATF5	activating transcription factor 5	down	1.54
204999_s_at	ATF5	activating transcription factor 5	down	1.60
212062_at	ATP9A	ATPase, class II, type 9A	down	1.86
212599_at	AUTS2	autism susceptibility candidate 2	up	1.43
206435_at	B4GALNT1	beta-1,4-N-acetyl-galactosaminyl transferase 1	up	2.19
1555385_at	B4GALNT1	beta-1,4-N-acetyl-galactosaminyl transferase 1	up	1.67

223632_s_at	BCAN	brevican	up	1.86
223633_s_at	BCAN	brevican	up	2.24
227341_at	BEND7	BEN domain containing 7	up	1.38
207399_at	BFSP2	beaded filament structural protein 2, phakinin	down	1.42
201169_s_at	BHLHE40	basic helix-loop-helix family, member e40	down	1.42
201170_s_at	BHLHE40	basic helix-loop-helix family, member e40	down	1.60
202201_at	BLVRB	biliverdin reductase B (flavin reductase (NADPH))	up	3.66
201848_s_at	BNIP3	BCL2/adenovirus E1B 19kDa interacting protein 3	down	1.41
201849_at	BNIP3	BCL2/adenovirus E1B 19kDa interacting protein 3	down	1.56
218954_s_at	BRF2	BRF2, subunit of RNA polymerase III transcription initiation factor, BRF1-like	up	1.66
218955_at	BRF2	BRF2, subunit of RNA polymerase III transcription initiation factor, BRF1-like	up	1.50
208906_at	BSCL2	Berardinelli-Seip congenital lipodystrophy 2 (seipin)	up	1.39
218792_s_at	BSPRY	B-box and SPRY domain containing	down	1.41
228434_at	BTNL9	butyrophilin-like 9	up	1.43
226313_at	C10orf35	chromosome 10 open reading frame 35	up	1.87
219806_s_at	C11orf75	chromosome 11 open reading frame 75	down	1.45
227099_s_at	C11orf96	chromosome 11 open reading frame 96	down	1.44
218723_s_at	C13orf15	chromosome 13 open reading frame 15	down	1.42
1556588_at	C15orf37	chromosome 15 open reading frame 37	down	1.46
214696_at	C17orf91	chromosome 17 open reading frame 91	up	1.92
229238_at	C17orf97	chromosome 17 open reading frame 97	up	1.42
220918_at	C21orf96	chromosome 21 open reading frame 96	up	1.51
212421_at	C22orf9	chromosome 22 open reading frame 9	down	1.41
217118_s_at	C22orf9	chromosome 22 open reading frame 9	down	1.51
227144_at	C22orf9	chromosome 22 open reading frame 9	down	1.49
1554176_a_at	C3orf33	chromosome 3 open reading frame 33	down	1.42
208451_s_at	C4A /// C4B ///	complement component 4A (Rodgers blood group) /// complement compo	down	1.44
236634_at	C8orf48	chromosome 8 open reading frame 48	up	1.61
59437_at	C9orf116	chromosome 9 open reading frame 116	up	1.43
239799_at	C9orf130	chromosome 9 open reading frame 130	up	1.37
227534_at	C9orf21	chromosome 9 open reading frame 21	up	1.36
225146_at	C9orf25	chromosome 9 open reading frame 25	up	1.45
209030_s_at	CADM1	cell adhesion molecule 1	down	1.44
209031_at	CADM1	cell adhesion molecule 1	down	1.39
209032_s_at	CADM1	cell adhesion molecule 1	down	1.47
229029_at	CAMK4	calcium/calmodulin-dependent protein kinase IV	up	1.44
208683_at	CAPN2	calpain 2, (m/II) large subunit	down	1.56
224414_s_at	CARD6	caspase recruitment domain family, member 6	up	1.63
1561405_s_at	CATSPER2	cation channel, sperm associated 2	up	1.36
203065_s_at	CAV1	caveolin 1, caveolae protein, 22kDa	down	1.52
212097_at	CAV1	caveolin 1, caveolae protein, 22kDa	down	1.48
209682_at	CBLB	Cas-Br-M (murine) ecotropic retroviral transforming sequence b	up	1.40
242301_at	CBLN2	cerebellin 2 precursor	up	1.49
205379_at	CBR3	carbonyl reductase 3	up	1.43
212816_s_at	CBS	cystathionine-beta-synthase	down	2.17
1553972_a_at	CBS	cystathionine-beta-synthase	down	1.88
1553645_at	CCDC141	coiled-coil domain containing 141	up	1.46
221912_s_at	CCDC28B	coiled-coil domain containing 28B	up	1.42
208712_at	CCND1	cyclin D1	down	1.90
213523_at	CCNE1	cyclin E1	up	1.42
219470_x_at	CCNJ	cyclin J	down	1.52
229091_s_at	CCNJ	cyclin J	down	1.47
220565_at	CCR10	chemokine (C-C motif) receptor 10	down	2.13
204306_s_at	CD151	CD151 molecule (Raph blood group)	up	1.59
214049_x_at	CD7	CD7 molecule	down	1.48
214551_s_at	CD7	CD7 molecule	down	1.61

213182_x_at	CDKN1C	cyclin-dependent kinase inhibitor 1C (p57, Kip2)	down	1.62
213183_s_at	CDKN1C	Cyclin-dependent kinase inhibitor 1C (p57, Kip2)	down	1.61
213348_at	CDKN1C	cyclin-dependent kinase inhibitor 1C (p57, Kip2)	down	1.64
216894_x_at	CDKN1C	cyclin-dependent kinase inhibitor 1C (p57, Kip2)	down	1.53
219534_x_at	CDKN1C	cyclin-dependent kinase inhibitor 1C (p57, Kip2)	down	1.46
226185_at	CDS1	CDP-diacylglycerol synthase (phosphatidate cytidylyltransferase) 1	down	1.43
213554_s_at	CDV3	CDV3 homolog (mouse)	down	1.43
223232_s_at	CGN	cingulin	down	1.36
242157_at	CHD9	Chromodomain helicase DNA binding protein 9	up	1.49
229954_at	CHDH	choline dehydrogenase	down	1.61
231994_at	CHDH	choline dehydrogenase	down	1.59
1559590_at	CHDH	choline dehydrogenase	down	1.81
200998_s_at	CKAP4	cytoskeleton-associated protein 4	down	1.53
200999_s_at	CKAP4	cytoskeleton-associated protein 4	down	1.45
204810_s_at	CKM	creatine kinase, muscle	up	1.54
1554406_a_at	CLEC7A	C-type lectin domain family 7, member A	down	1.38
1555756_a_at	CLEC7A	C-type lectin domain family 7, member A	down	1.42
208791_at	CLU	clusterin	up	2.88
208792_s_at	CLU	clusterin	up	2.83
222043_at	CLU	clusterin	up	1.56
235099_at	CMTM8	CKLF-like MARVEL transmembrane domain containing 8	up	1.54
203642_s_at	COBLL1	COBL-like 1	down	1.40
225288_at	COL27A1	collagen, type XXVII, alpha 1	up	1.37
204724_s_at	COL9A3	collagen, type IX, alpha 3	down	1.40
201116_s_at	CPE	carboxypeptidase E	down	1.65
201117_s_at	CPE	carboxypeptidase E	down	1.61
228759_at	CREB3L2	cAMP responsive element binding protein 3-like 2	down	1.36
226455_at	CREB3L4	cAMP responsive element binding protein 3-like 4	down	1.46
218358_at	CRELD2	cysteine-rich with EGF-like domains 2	down	1.41
209967_s_at	CREM	cAMP responsive element modulator	up	1.49
228092_at	CREM	cAMP responsive element modulator	up	1.45
230511_at	CREM	cAMP responsive element modulator	up	1.38
205081_at	CRIP1	cysteine-rich protein 1 (intestinal)	up	2.32
206843_at	CRYBA4	crystallin, beta A4	up	1.61
205159_at	CSF2RB	colony stimulating factor 2 receptor, beta, low-affinity (granulocyte-macrop	down	1.57
225681_at	CTHRC1	collagen triple helix repeat containing 1	down	1.66
200838_at	CTSB	cathepsin B	up	1.42
213274_s_at	CTSB	cathepsin B	up	1.39
227961_at	CTSB	cathepsin B	up	1.40
200766_at	CTSD	cathepsin D	up	1.48
232136_s_at	CTTNBP2	cortactin binding protein 2	down	1.37
227757_at	CUL4A	cullin 4A	down	1.36
203532_x_at	CUL5	cullin 5	down	1.38
205898_at	CX3CR1	chemokine (C-X3-C motif) receptor 1	down	2.30
204533_at	CXCL10	chemokine (C-X-C motif) ligand 10	up	1.52
202263_at	CYB5R1	cytochrome b5 reductase 1	up	1.51
227109_at	CYP2R1	cytochrome P450, family 2, subfamily R, polypeptide 1	down	1.65
220432_s_at	CYP39A1	cytochrome P450, family 39, subfamily A, polypeptide 1	up	2.04
244407_at	CYP39A1	cytochrome P450, family 39, subfamily A, polypeptide 1	up	2.88
1553977_a_at	CYP39A1	cytochrome P450, family 39, subfamily A, polypeptide 1	up	1.60
230866_at	CYSLTR1	cysteinyl leukotriene receptor 1	down	1.82
231747_at	CYSLTR1	cysteinyl leukotriene receptor 1	down	1.72
205471_s_at	DACH1	dachshund homolog 1 (Drosophila)	down	1.36
228915_at	DACH1	dachshund homolog 1 (Drosophila)	down	1.90
202806_at	DBN1	drebrin 1	down	1.80
204850_s_at	DCX	doublecortin	up	1.61

204851_s_at	DCX	doublecortin	up	1.51
202887_s_at	DDIT4	DNA-damage-inducible transcript 4	down	1.66
218986_s_at	DDX60	DEAD (Asp-Glu-Ala-Asp) box polypeptide 60	up	1.67
228152_s_at	DDX60L	DEAD (Asp-Glu-Ala-Asp) box polypeptide 60-like	up	1.44
212561_at	DENND5A	DENN/MADD domain containing 5A	up	1.72
203385_at	DGKA	diacylglycerol kinase, alpha 80kDa	down	1.49
211272_s_at	DGKA	diacylglycerol kinase, alpha 80kDa	down	1.44
206395_at	DGKG	diacylglycerol kinase, gamma 90kDa	up	1.69
200862_at	DHCR24	24-dehydrocholesterol reductase	down	1.49
218976_at	DNAJC12	DnaJ (Hsp40) homolog, subfamily C, member 12	down	1.62
223371_s_at	DNAJC4	DnaJ (Hsp40) homolog, subfamily C, member 4	up	1.63
204008_at	DNAL4	dynein, axonemal, light chain 4	up	1.43
215116_s_at	DNM1	dynamin 1	up	1.40
244428_at	DNMT3A	DNA (cytosine-5-)-methyltransferase 3 alpha	down	1.43
225502_at	DOCK8	dedicator of cytokinesis 8	down	2.20
232843_s_at	DOCK8	dedicator of cytokinesis 8	down	1.60
214844_s_at	DOK5	docking protein 5	up	1.38
230158_at	DPY19L2	dpy-19-like 2 (C. elegans)	down	1.43
1554534_at	DPYD	dihydropyrimidine dehydrogenase	up	1.45
208891_at	DUSP6	dual specificity phosphatase 6	down	1.54
208892_s_at	DUSP6	dual specificity phosphatase 6	down	1.70
211928_at	DYNC1H1	dynein, cytoplasmic 1, heavy chain 1	up	1.53
205348_s_at	DYNC1I1	dynein, cytoplasmic 1, intermediate chain 1	up	2.20
1569843_at	DYNC1I1	dynein, cytoplasmic 1, intermediate chain 1	up	1.41
204271_s_at	EDNRB	endothelin receptor type B	down	2.78
204273_at	EDNRB	endothelin receptor type B	down	3.81
206701_x_at	EDNRB	endothelin receptor type B	down	3.10
223608_at	EFCAB2	EF-hand calcium binding domain 2	up	1.45
206580_s_at	EFEMP2	EGF-containing fibulin-like extracellular matrix protein 2	up	1.56
209356_x_at	EFEMP2	EGF-containing fibulin-like extracellular matrix protein 2	up	1.99
1558411_at	EGFEM1P	EGF-like and EMI domain containing 1, pseudogene	up	1.51
221497_x_at	EGLN1	egl nine homolog 1 (C. elegans)	down	1.45
223046_at	EGLN1	egl nine homolog 1 (C. elegans)	down	1.38
224314_s_at	EGLN1	egl nine homolog 1 (C. elegans)	down	1.51
231292_at	EID3	EP300 interacting inhibitor of differentiation 3	up	1.45
218696_at	EIF2AK3	eukaryotic translation initiation factor 2-alpha kinase 3	down	1.39
237145_at	EIF2AK4	eukaryotic translation initiation factor 2 alpha kinase 4	down	1.52
203490_at	ELF4	E74-like factor 4 (ets domain transcription factor)	up	1.36
1559072_a_at	ELFN2	extracellular leucine-rich repeat and fibronectin type III domain containing	down	1.50
226982_at	ELL2	elongation factor, RNA polymerase II, 2	down	1.37
203729_at	EMP3	epithelial membrane protein 3	up	2.06
1561396_at	EPHA6	EPH receptor A6	down	1.41
227609_at	EPSTI1	epithelial stromal interaction 1 (breast)	down	1.36
235745_at	ERN1	endoplasmic reticulum to nucleus signaling 1	down	1.41
218498_s_at	ERO1L	ERO1-like (S. cerevisiae)	down	1.41
222646_s_at	ERO1L	ERO1-like (S. cerevisiae)	down	1.48
225846_at	ESRP1	epithelial splicing regulatory protein 1	down	2.06
203349_s_at	ETV5	ets variant 5	up	1.37
221664_s_at	F11R	F11 receptor	down	1.42
223000_s_at	F11R	F11 receptor	down	1.42
224097_s_at	F11R	F11 receptor	down	1.43
208962_s_at	FADS1	fatty acid desaturase 1	down	2.07
208964_s_at	FADS1	fatty acid desaturase 1	down	1.89
216080_s_at	FADS3	fatty acid desaturase 3	up	1.37
221602_s_at	FAIM3	Fas apoptotic inhibitory molecule 3	down	1.62
217967_s_at	FAM129A	family with sequence similarity 129, member A	up	1.46

218532_s_at	FAM134B	family with sequence similarity 134, member B	down	1.40
214889_at	FAM149A	family with sequence similarity 149, member A	down	1.43
222291_at	FAM149A	family with sequence similarity 149, member A	down	1.38
235850_at	FAM162A	family with sequence similarity 162, member A	down	1.59
229655_at	FAM19A5	family with sequence similarity 19 (chemokine (C-C motif)-like), member A5	down	1.42
1557014_a_at	FAM201A	family with sequence similarity 201, member A	down	1.48
227410_at	FAM43A	family with sequence similarity 43, member A	up	1.43
226811_at	FAM46C	family with sequence similarity 46, member C	down	1.40
221774_x_at	FAM48A	family with sequence similarity 48, member A	down	1.47
226330_s_at	FAM48A	family with sequence similarity 48, member A	down	1.42
217540_at	FAM55C	family with sequence similarity 55, member C	up	1.39
219895_at	FAM70A	family with sequence similarity 70, member A	up	1.62
203184_at	FBN2	fibrillin 2	down	1.71
209696_at	FBP1	fructose-1,6-bisphosphatase 1	up	1.49
44040_at	FBXO41	F-box protein 41	down	1.44
203620_s_at	FCHSD2	FCH and double SH3 domains 2	down	1.42
204422_s_at	FGF2	fibroblast growth factor 2 (basic)	up	1.49
206742_at	FIGF	c-fos induced growth factor (vascular endothelial growth factor D)	up	1.49
219117_s_at	FKBP11	FK506 binding protein 11, 19 kDa	down	1.66
219118_at	FKBP11	FK506 binding protein 11, 19 kDa	down	1.79
228308_at	FKBP11	FK506 binding protein 11, 19 kDa	down	1.70
224002_s_at	FKBP7	FK506 binding protein 7	down	1.39
231130_at	FKBP7	FK506 binding protein 7	down	1.38
208614_s_at	FLNB	filamin B, beta	up	1.40
223950_s_at	FLYWCH1	FLYWCH-type zinc finger 1	up	1.39
234106_s_at	FLYWCH1	FLYWCH-type zinc finger 1	up	1.40
229893_at	FRMD3	FERM domain containing 3	up	1.73
230645_at	FRMD3	FERM domain containing 3	up	1.66
242600_at	FRMD3	FERM domain containing 3	up	1.68
230831_at	FRMD5	FERM domain containing 5	down	1.40
206109_at	FUT1	fucosyltransferase 1 (galactoside 2-alpha-L-fucosyltransferase, H blood grou	down	1.44
202812_at	GAA	glucosidase, alpha; acid	up	1.42
229114_at	GAB1	GRB2-associated binding protein 1	down	1.37
208868_s_at	GABARAPL1	GABA(A) receptor-associated protein like 1	up	1.86
208869_s_at	GABARAPL1	GABA(A) receptor-associated protein like 1	up	2.30
211458_s_at	GABARAPL1 ///	GABA(A) receptor-associated protein like 1 /// GABA(A) receptors associate	up	2.34
205890_s_at	GABBR1 /// UB	gamma-aminobutyric acid (GABA) B receptor, 1 /// ubiquitin D	up	1.56
202192_s_at	GAS7	growth arrest-specific 7	down	1.39
207704_s_at	GAS7	growth arrest-specific 7	down	1.62
211067_s_at	GAS7	growth arrest-specific 7	down	1.57
208503_s_at	GATAD1	GATA zinc finger domain containing 1	down	1.39
203178_at	GATM	glycine amidinotransferase (L-arginine:glycine amidinotransferase)	down	1.55
216733_s_at	GATM	glycine amidinotransferase (L-arginine:glycine amidinotransferase)	down	1.51
210589_s_at	GBAP1	glucosidase, beta, acid pseudogene 1	up	1.36
203282_at	GBE1	glucan (1,4-alpha-), branching enzyme 1	down	1.48
202269_x_at	GBP1	guanylate binding protein 1, interferon-inducible, 67kDa	up	1.55
202270_at	GBP1	guanylate binding protein 1, interferon-inducible, 67kDa	up	1.53
231577_s_at	GBP1	guanylate binding protein 1, interferon-inducible, 67kDa	up	1.50
223434_at	GBP3	guanylate binding protein 3	up	2.02
234986_at	GCLM	glutamate-cysteine ligase, modifier subunit	up	1.58
236140_at	GCLM	glutamate-cysteine ligase, modifier subunit	up	1.41
228776_at	GJC1	gap junction protein, gamma 1, 45kDa	up	1.51
214430_at	GLA	galactosidase, alpha	up	1.54
204836_at	GLDC	glycine dehydrogenase (decarboxylating)	down	1.66
204222_s_at	GLIPR1	GLI pathogenesis-related 1	up	1.44
216341_s_at	GNRHR	gonadotropin-releasing hormone receptor	up	1.37

201141_at	GPNMB	glycoprotein (transmembrane) nmb	up	1.82
221297_at	GPRC5D	G protein-coupled receptor, family C, group 5, member D	down	1.62
206204_at	GRB14	growth factor receptor-bound protein 14	down	1.82
235405_at	GSTA4	glutathione S-transferase alpha 4	up	1.51
203817_at	GUCY1B3	guanylate cyclase 1, soluble, beta 3	up	1.41
211555_s_at	GUCY1B3	guanylate cyclase 1, soluble, beta 3	up	1.42
204235_s_at	GULP1	GULP, engulfment adaptor PTB domain containing 1	up	1.66
204237_at	GULP1	GULP, engulfment adaptor PTB domain containing 1	up	1.64
215913_s_at	GULP1	GULP, engulfment adaptor PTB domain containing 1	up	1.68
224301_x_at	H2AFJ	H2A histone family, member J	down	1.54
225245_x_at	H2AFJ	H2A histone family, member J	down	1.56
208579_x_at	H2BFS	H2B histone family, member S	down	1.90
229208_at	HAUS2	HAUS augmin-like complex, subunit 2	down	1.46
206834_at	HBD	hemoglobin, delta	down	1.47
205659_at	HDAC9	histone deacetylase 9	down	1.41
1552760_at	HDAC9	histone deacetylase 9	down	1.37
1554478_a_at	HEATR3	HEAT repeat containing 3	down	1.38
223669_at	HEMGN	hemogen	down	1.39
218839_at	HEY1	hairy/enhancer-of-split related with YRPW motif 1	up	1.42
44783_s_at	HEY1	hairy/enhancer-of-split related with YRPW motif 1	up	1.43
210997_at	HGF	hepatocyte growth factor (hepapoietin A; scatter factor)	down	1.37
204689_at	HHEX	hematopoietically expressed homeobox	up	1.37
208461_at	HIC1	hypermethylated in cancer 1	up	1.42
230218_at	HIC1	hypermethylated in cancer 1	up	1.72
209398_at	HIST1H1C	histone cluster 1, H1c	down	1.85
215071_s_at	HIST1H2AC	histone cluster 1, H2ac	down	2.20
214472_at	HIST1H2AD ///	histone cluster 1, H2ad /// histone cluster 1, H3d	down	1.38
214522_x_at	HIST1H2AD ///	histone cluster 1, H2ad /// histone cluster 1, H3d	down	1.46
214469_at	HIST1H2AE	histone cluster 1, H2ae	down	1.63
214554_at	HIST1H2AL	histone cluster 1, H2al	down	1.95
214481_at	HIST1H2AM	histone cluster 1, H2am	down	1.40
214455_at	HIST1H2BC	histone cluster 1, H2bc	down	2.05
209911_x_at	HIST1H2BD	histone cluster 1, H2bd	down	1.76
222067_x_at	HIST1H2BD	histone cluster 1, H2bd	down	1.66
208527_x_at	HIST1H2BE	histone cluster 1, H2be	down	1.72
208490_x_at	HIST1H2BF	histone cluster 1, H2bf	down	1.68
208546_x_at	HIST1H2BH	histone cluster 1, H2bh	down	1.91
208523_x_at	HIST1H2BI	histone cluster 1, H2bi	down	1.51
214502_at	HIST1H2BJ	histone cluster 1, H2bj	down	1.44
209806_at	HIST1H2BK	histone cluster 1, H2bk	down	1.50
206110_at	HIST1H3H	histone cluster 1, H3h	down	1.47
208180_s_at	HIST1H4H	histone cluster 1, H4h	down	1.37
214463_x_at	HIST1H4J	histone cluster 1, H4j	down	1.45
214290_s_at	HIST2H2AA3 //,	histone cluster 2, H2aa3 /// histone cluster 2, H2aa4	down	1.82
218280_x_at	HIST2H2AA3 //,	histone cluster 2, H2aa3 /// histone cluster 2, H2aa4	down	2.00
202708_s_at	HIST2H2BE	histone cluster 2, H2be	down	1.91
227614_at	HKDC1	hexokinase domain containing 1	down	1.60
201137_s_at	HLA-DPB1	major histocompatibility complex, class II, DP beta 1	down	1.97
202983_at	HLTF	helicase-like transcription factor	down	4.09
224734_at	HMGB1	high-mobility group box 1	down	1.46
205822_s_at	HMGCS1	3-hydroxy-3-methylglutaryl-CoA synthase 1 (soluble)	down	2.45
221750_at	HMGCS1	3-hydroxy-3-methylglutaryl-CoA synthase 1 (soluble)	down	1.74
211597_s_at	HOPX	HOP homeobox	up	1.76
214639_s_at	HOXA1	homeobox A1	up	1.38
204778_x_at	HOXB7	homeobox B7	up	1.42
204779_s_at	HOXB7	homeobox B7	up	1.62

216973_s_at	HOXB7	homeobox B7	up	1.56
203913_s_at	HPGD	hydroxyprostaglandin dehydrogenase 15-(NAD)	down	1.61
211548_s_at	HPGD	hydroxyprostaglandin dehydrogenase 15-(NAD)	down	1.43
211549_s_at	HPGD	hydroxyprostaglandin dehydrogenase 15-(NAD)	down	1.45
1554122_a_at	HSD17B12	hydroxysteroid (17-beta) dehydrogenase 12	up	1.48
200598_s_at	HSP90B1	heat shock protein 90kDa beta (Grp94), member 1	down	1.37
209448_at	HTATIP2	HIV-1 Tat interactive protein 2, 30kDa	up	1.37
204569_at	ICK	intestinal cell (MAK-like) kinase	up	1.53
208937_s_at	ID1	inhibitor of DNA binding 1, dominant negative helix-loop-helix protein	up	1.58
1555037_a_at	IDH1	isocitrate dehydrogenase 1 (NADP+), soluble	down	1.37
214453_s_at	IFI44	interferon-induced protein 44	up	2.14
204747_at	IFIT3	interferon-induced protein with tetratricopeptide repeats 3	up	1.51
229450_at	IFIT3	interferon-induced protein with tetratricopeptide repeats 3	up	1.56
209540_at	IGF1	insulin-like growth factor 1 (somatomedin C)	down	1.65
209541_at	IGF1	insulin-like growth factor 1 (somatomedin C)	down	1.58
209542_x_at	IGF1	insulin-like growth factor 1 (somatomedin C)	down	1.45
211577_s_at	IGF1	insulin-like growth factor 1 (somatomedin C)	down	1.42
202718_at	IGFBP2	insulin-like growth factor binding protein 2, 36kDa	down	1.46
201508_at	IGFBP4	insulin-like growth factor binding protein 4	up	1.57
1558438_a_at	IGHE	immunoglobulin heavy constant epsilon	up	1.91
211430_s_at	IGHG1 /// IGHG	immunoglobulin heavy constant gamma 1 (G1m marker) /// immunoglobul	down	1.64
209827_s_at	IL16	interleukin 16 (lymphocyte chemoattractant factor)	down	1.62
219255_x_at	IL17RB	interleukin 17 receptor B	down	2.19
224156_x_at	IL17RB	interleukin 17 receptor B	down	2.01
224361_s_at	IL17RB	interleukin 17 receptor B	down	1.66
203828_s_at	IL32	interleukin 32	down	1.71
210587_at	INHBE	inhibin, beta E	down	1.40
223878_at	INPP4B	inositol polyphosphate-4-phosphatase, type II, 105kDa	down	1.39
201625_s_at	INSIG1	insulin induced gene 1	down	1.39
204202_at	IQCE	IQ motif containing E	up	1.40
204030_s_at	IQCJ-SCHIP1 ///	IQ motif containing J-schwannomin interacting protein 1 read-through tran	down	1.44
222240_s_at	ISYNA1	inositol-3-phosphate synthase 1	up	1.36
205884_at	ITGA4	integrin, alpha 4 (antigen CD49D, alpha 4 subunit of VLA-4 receptor)	down	1.46
205885_s_at	ITGA4	integrin, alpha 4 (antigen CD49D, alpha 4 subunit of VLA-4 receptor)	down	1.45
214265_at	ITGA8	integrin, alpha 8	down	1.48
235666_at	ITGA8	integrin, alpha 8	down	1.61
213478_at	KAZ	kazrin	down	2.72
229144_at	KAZ	kazrin	down	1.52
223412_at	KBTBD7	kelch repeat and BTB (POZ) domain containing 7	down	1.44
239118_at	KCNA2	potassium voltage-gated channel, shaker-related subfamily, member 2	down	1.39
207237_at	KCNA3	potassium voltage-gated channel, shaker-related subfamily, member 3	down	1.53
204487_s_at	KCNQ1	potassium voltage-gated channel, KQT-like subfamily, member 1	down	1.38
205968_at	KCNS3	potassium voltage-gated channel, delayed-rectifier, subfamily S, member 3	up	1.71
200922_at	KDELR1	KDEL (Lys-Asp-Glu-Leu) endoplasmic reticulum protein retention receptor 1	down	1.37
204017_at	KDELR3	KDEL (Lys-Asp-Glu-Leu) endoplasmic reticulum protein retention receptor 3	down	1.76
206478_at	KIAA0125	KIAA0125	down	1.43
232366_at	KIAA0232	KIAA0232	up	1.52
212427_at	KIAA0368	KIAA0368	up	1.40
1554438_at	KIAA1217	KIAA1217	down	1.53
231856_at	KIAA1244	KIAA1244	down	1.48
232166_at	KIAA1377	KIAA1377	up	1.87
235956_at	KIAA1377	KIAA1377	up	1.64
236325_at	KIAA1377	KIAA1377	up	1.48
243539_at	KIAA1841	KIAA1841	up	1.83
226003_at	KIF21A	kinesin family member 21A	up	1.57
231875_at	KIF21A	kinesin family member 21A	up	1.74

242517_at	KISS1R	KISS1 receptor	down	1.77
221841_s_at	KLF4	Kruppel-like factor 4 (gut)	up	1.66
240432_x_at	KLF7	Kruppel-like factor 7 (ubiquitous)	up	1.48
225961_at	KLHDC5	kelch domain containing 5	down	1.44
221837_at	KLHL22	kelch-like 22 (Drosophila)	up	1.51
221838_at	KLHL22	kelch-like 22 (Drosophila)	up	1.65
228167_at	KLHL6	kelch-like 6 (Drosophila)	down	3.32
1555275_a_at	KLHL6	kelch-like 6 (Drosophila)	down	1.55
1560396_at	KLHL6	kelch-like 6 (Drosophila)	down	1.82
201596_x_at	KRT18	keratin 18	down	1.96
215189_at	KRT86 /// LOC1	keratin 86 /// hypothetical LOC100509764	down	1.46
234679_at	KRTAP9-3	keratin associated protein 9-3	up	1.47
233640_x_at	KRTAP9-4	keratin associated protein 9-4	up	1.72
234639_x_at	KRTAP9-8	keratin associated protein 9-8	up	1.94
220972_s_at	KRTAP9-9	keratin associated protein 9-9	up	1.90
235252_at	KSR1	kinase suppressor of ras 1	up	1.36
208071_s_at	LAIR1	leukocyte-associated immunoglobulin-like receptor 1	down	1.64
210644_s_at	LAIR1	leukocyte-associated immunoglobulin-like receptor 1	down	2.54
1554038_at	LARP1B	La ribonucleoprotein domain family, member 1B	down	1.40
207734_at	LAX1	lymphocyte transmembrane adaptor 1	down	1.39
204890_s_at	LCK	lymphocyte-specific protein tyrosine kinase	down	1.44
204891_s_at	LCK	lymphocyte-specific protein tyrosine kinase	down	1.60
223228_at	LDOC1L	leucine zipper, down-regulated in cancer 1-like	up	1.41
209894_at	LEPR	leptin receptor	down	1.36
211354_s_at	LEPR	leptin receptor	down	1.38
220158_at	LGALS14	lectin, galactoside-binding, soluble, 14	down	1.43
208933_s_at	LGALS8	lectin, galactoside-binding, soluble, 8	up	1.54
208934_s_at	LGALS8	lectin, galactoside-binding, soluble, 8	up	1.54
208935_s_at	LGALS8	lectin, galactoside-binding, soluble, 8	up	1.64
208936_x_at	LGALS8	lectin, galactoside-binding, soluble, 8	up	1.41
210732_s_at	LGALS8	lectin, galactoside-binding, soluble, 8	up	1.57
218326_s_at	LGR4	leucine-rich repeat-containing G protein-coupled receptor 4	down	1.41
236048_at	LHFPL1	lipoma HMGIC fusion partner-like 1	up	1.57
206140_at	LHX2	LIM homeobox 2	up	2.51
211219_s_at	LHX2	LIM homeobox 2	up	1.49
210152_at	LILRB4	leukocyte immunoglobulin-like receptor, subfamily B (with TM and ITIM do	up	1.85
217892_s_at	LIMA1	LIM domain and actin binding 1	down	1.61
218600_at	LIMD2	LIM domain containing 2	down	1.43
203411_s_at	LMNA	lamin A/C	up	1.49
1554600_s_at	LMNA	lamin A/C	up	1.50
236008_at	LOC100128909	hypothetical LOC100128909	down	1.40
1559871_s_at	LOC100129129	hypothetical LOC100129129	up	1.47
235990_at	LOC100130987	similar to hCG1815675	up	1.37
227234_at	LOC100132815	hypothetical LOC100132815	up	1.47
236260_at	LOC100287598	hypothetical LOC100287598	down	1.41
228381_at	LOC100287628	Hypothetical protein LOC100287628	down	1.44
1557638_at	LOC100287676	Hypothetical protein LOC100287676	down	1.57
219785_s_at	LOC100288525	hypothetical protein LOC100288525	down	1.45
220354_at	LOC100289410	hypothetical LOC100289410	down	1.51
227655_at	LOC100505806	hypothetical LOC100505806 /// small nucleolar RNA, C/D box 123	down	1.70
221973_at	LOC100506076	hypothetical LOC100506076 /// hypothetical LOC100506123	up	1.37
229296_at	LOC100506119	hypothetical LOC100506119 /// hypothetical LOC100508342	down	1.49
236193_at	LOC100506979	hypothetical LOC100506979	down	1.88
244764_at	LOC100507198	hypothetical LOC100507198	up	1.60
214945_at	LOC100507397	hypothetical LOC100507397	down	1.85
1569122_at	LOC100509533	hypothetical LOC100509533	up	1.37

1561343_a_at	LOC150005	hypothetical protein LOC150005	up	1.40
236124_at	LOC153546	hypothetical protein LOC153546	down	1.46
237271_at	LOC154872	hypothetical protein LOC154872	up	1.39
232034_at	LOC203274	Hypothetical protein LOC203274	up	1.36
214162_at	LOC284244	hypothetical protein LOC284244	up	1.56
1555847_a_at	LOC284454	hypothetical LOC284454	up	1.39
236351_at	LOC389023	hypothetical LOC389023	up	1.39
229094_at	LOC401431	hypothetical LOC401431	up	1.37
1569110_x_at	LOC728613	programmed cell death 6 pseudogene	down	1.61
221833_at	LONP2	Lon peptidase 2, peroxisomal	up	1.39
227145_at	LOXL4	lysyl oxidase-like 4	down	1.52
227889_at	LPCAT2	lysophosphatidylcholine acyltransferase 2	up	1.37
219631_at	LRP12	low density lipoprotein receptor-related protein 12	up	1.41
220253_s_at	LRP12	low density lipoprotein receptor-related protein 12	up	1.46
220254_at	LRP12	low density lipoprotein receptor-related protein 12	up	1.39
209468_at	LRP5	low density lipoprotein receptor-related protein 5	down	1.48
219441_s_at	LRRK1	leucine-rich repeat kinase 1	down	1.42
226884_at	LRRN1	leucine rich repeat neuronal 1	up	2.11
202145_at	LY6E	lymphocyte antigen 6 complex, locus E	down	1.49
206584_at	LY96	lymphocyte antigen 96	up	1.47
209942_x_at	MAGEA3	melanoma antigen family A, 3	down	1.76
214612_x_at	MAGEA6	melanoma antigen family A, 6	down	1.62
224650_at	MAL2	mal, T-cell differentiation protein 2	down	1.56
224558_s_at	MALAT1	metastasis associated lung adenocarcinoma transcript 1 (non-protein codin	up	1.36
224567_x_at	MALAT1	metastasis associated lung adenocarcinoma transcript 1 (non-protein codin	up	1.39
231735_s_at	MALAT1	metastasis associated lung adenocarcinoma transcript 1 (non-protein codin	up	1.36
205088_at	MAMLD1	mastermind-like domain containing 1	up	1.73
209166_s_at	MAN2B1	mannosidase, alpha, class 2B, member 1	up	1.44
203841_x_at	MAPRE3	microtubule-associated protein, RP/EB family, member 3	up	1.38
203929_s_at	MAPT	microtubule-associated protein tau	up	1.69
203930_s_at	MAPT	microtubule-associated protein tau	up	1.80
221047_s_at	MARK1	MAP/microtubule affinity-regulating kinase 1	up	1.45
226653_at	MARK1	MAP/microtubule affinity-regulating kinase 1	up	1.65
226726_at	MBOAT2	membrane bound O-acyltransferase domain containing 2	up	1.39
226225_at	MCC	mutated in colorectal cancers	down	1.78
212935_at	MCF2L	MCF.2 cell line derived transforming sequence-like	down	1.55
35147_at	MCF2L	MCF.2 cell line derived transforming sequence-like	down	1.51
229797_at	MCOLN3	mucolipin 3	down	1.38
220122_at	MCTP1	multiple C2 domains, transmembrane 1	up	1.44
235740_at	MCTP1	multiple C2 domains, transmembrane 1	up	1.72
220603_s_at	MCTP2	multiple C2 domains, transmembrane 2	down	1.42
1558077_s_at	MDH1B	malate dehydrogenase 1B, NAD (soluble)	up	1.37
225160_x_at	MDM2	Mdm2 p53 binding protein homolog (mouse)	down	1.56
209200_at	MEF2C	myocyte enhancer factor 2C	down	1.42
230011_at	MEI1	meiosis inhibitor 1	down	1.49
1554208_at	MEI1	meiosis inhibitor 1	down	1.43
219858_s_at	MFSD6	major facilitator superfamily domain containing 6	down	1.37
1553708_at	MGC16075	hypothetical protein MGC16075	down	1.85
221286_s_at	MGC29506	plasma cell-induced ER protein 1	down	1.41
223565_at	MGC29506	plasma cell-induced ER protein 1	down	1.42
242136_x_at	MGC70870	C-terminal binding protein 2 pseudogene	down	1.36
219332_at	MICALL2	MICAL-like 2	up	2.99
204918_s_at	MLLT3	myeloid/lymphoid or mixed-lineage leukemia (trithorax homolog, Drosophi	down	1.38
220850_at	MORC1	MORC family CW-type zinc finger 1	down	1.37
233917_s_at	MOV10	Mov10, Moloney leukemia virus 10, homolog (mouse)	up	1.36
235352_at	MR1	major histocompatibility complex, class I-related	up	1.56

212277_at	MTMR4	myotubularin related protein 4	down	1.38
212096_s_at	MTUS1	microtubule associated tumor suppressor 1	down	1.39
213693_s_at	MUC1	mucin 1, cell surface associated	down	1.36
225673_at	MYADM	myeloid-associated differentiation marker	down	1.48
206717_at	MYH8	myosin, heavy chain 8, skeletal muscle, perinatal	up	1.46
224823_at	MYLK	myosin light chain kinase	down	1.54
212338_at	MYO1D	myosin ID	down	1.38
244364_at	MYO3A	myosin IIIA	up	1.37
218966_at	MYO5C	myosin VC	down	1.40
213375_s_at	N4BP2L1	NEDD4 binding protein 2-like 1	down	1.37
214753_at	N4BP2L2	NEDD4 binding protein 2-like 2	down	1.44
212993_at	NACC2	NACC family member 2, BEN and BTB (POZ) domain containing	down	1.38
219368_at	NAP1L2	nucleosome assembly protein 1-like 2	up	1.52
228062_at	NAP1L5	nucleosome assembly protein 1-like 5	down	1.72
228063_s_at	NAP1L5	nucleosome assembly protein 1-like 5	down	1.77
224772_at	NAV1	neuron navigator 1	down	1.65
224773_at	NAV1	neuron navigator 1	down	1.61
224774_s_at	NAV1	neuron navigator 1	down	1.58
1556017_at	NBEAL2	neurobeachin-like 2	up	1.41
211685_s_at	NCALD	neurocalcin delta	down	1.43
224799_at	NDFIP2	Nedd4 family interacting protein 2	down	1.44
224802_at	NDFIP2	Nedd4 family interacting protein 2	down	1.47
1554010_at	NDST1	N-deacetylase/N-sulfotransferase (heparan glucosaminyl) 1	down	1.38
202149_at	NEDD9	neural precursor cell expressed, developmentally down-regulated 9	down	1.80
224976_at	NFIA	nuclear factor I/A	down	1.36
226806_s_at	NFIA	nuclear factor I/A	down	1.54
209289_at	NFIB	nuclear factor I/B	up	1.54
209290_s_at	NFIB	nuclear factor I/B	up	1.73
213032_at	NFIB	nuclear factor I/B	up	1.40
203574_at	NFIL3	nuclear factor, interleukin 3 regulated	down	1.39
218557_at	NIT2	nitrilase family, member 2	up	1.48
207031_at	NKX3-2	NK3 homeobox 2	up	1.77
203964_at	NMI	N-myc (and STAT) interactor	up	1.64
235410_at	NPHP3	nephronophthisis 3 (adolescent)	down	1.37
221210_s_at	NPL	N-acetylneuraminate pyruvate lyase (dihydrodipicolinate synthase)	up	1.60
223405_at	NPL	N-acetylneuraminate pyruvate lyase (dihydrodipicolinate synthase)	up	1.50
240440_at	NPL	N-acetylneuraminate pyruvate lyase (dihydrodipicolinate synthase)	up	1.46
201467_s_at	NQO1	NAD(P)H dehydrogenase, quinone 1	up	1.45
210519_s_at	NQO1	NAD(P)H dehydrogenase, quinone 1	up	1.40
203814_s_at	NQO2	NAD(P)H dehydrogenase, quinone 2	up	1.52
203939_at	NT5E	5'-nucleotidase, ecto (CD73)	down	1.49
1553994_at	NT5E	5'-nucleotidase, ecto (CD73)	down	1.45
1553995_a_at	NT5E	5'-nucleotidase, ecto (CD73)	down	1.61
236930_at	NUMB	Numb homolog (Drosophila)	down	1.37
219489_s_at	NXN	nucleoredoxin	up	2.17
204972_at	OAS2	2'-5'-oligoadenylate synthetase 2, 69/71kDa	down	1.42
217551_at	OR7E14P	olfactory receptor, family 7, subfamily E, member 14 pseudogene	down	1.74
223464_at	OSBPL5	oxysterol binding protein-like 5	up	2.17
219475_at	OSGIN1	oxidative stress induced growth inhibitor 1	up	1.49
207543_s_at	P4HA1	prolyl 4-hydroxylase, alpha polypeptide I	down	1.63
202733_at	P4HA2	prolyl 4-hydroxylase, alpha polypeptide II	down	1.43
227053_at	PACSIN1	protein kinase C and casein kinase substrate in neurons 1	down	1.98
239067_s_at	PANX2	pannexin 2	up	1.40
203060_s_at	PAPSS2	3'-phosphoadenosine 5'-phosphosulfate synthase 2	down	1.66
204629_at	PARVB	parvin, beta	down	1.46
37965_at	PARVB	parvin, beta	down	1.40

37966_at	PARVB	parvin, beta	down	1.41
228635_at	PCDH10	protocadherin 10	up	1.81
231726_at	PCDHB14	protocadherin beta 14	down	1.51
231789_at	PCDHB15	protocadherin beta 15	up	1.57
228905_at	PCM1	pericentriolar material 1	up	1.43
205549_at	PCP4	Purkinje cell protein 4	down	2.51
205463_s_at	PDGFA	platelet-derived growth factor alpha polypeptide	down	2.12
229830_at	PDGFA	Platelet-derived growth factor alpha polypeptide	down	2.87
206691_s_at	PDIA2	protein disulfide isomerase family A, member 2	down	2.15
208612_at	PDIA3	protein disulfide isomerase family A, member 3	down	1.58
208658_at	PDIA4	protein disulfide isomerase family A, member 4	down	1.36
206686_at	PDK1	pyruvate dehydrogenase kinase, isozyme 1	down	1.43
226452_at	PDK1	pyruvate dehydrogenase kinase, isozyme 1	down	1.51
208982_at	PECAM1	platelet/endothelial cell adhesion molecule	down	1.39
208983_s_at	PECAM1	platelet/endothelial cell adhesion molecule	down	1.38
228499_at	PFKFB4	6-phosphofructo-2-kinase/fructose-2,6-biphosphatase 4	down	2.02
220944_at	PGLYRP4	peptidoglycan recognition protein 4	up	1.39
239229_at	PHEX	phosphate regulating endopeptidase homolog, X-linked	up	1.50
221816_s_at	PHF11	PHD finger protein 11	up	1.36
231967_at	PHF20L1	PHD finger protein 20-like 1	down	1.43
201397_at	PHGDH	phosphoglycerate dehydrogenase	down	1.62
217997_at	PHLDA1	pleckstrin homology-like domain, family A, member 1	down	1.36
217999_s_at	PHLDA1	pleckstrin homology-like domain, family A, member 1	down	1.36
225842_at	PHLDA1	pleckstrin homology-like domain, family A, member 1	down	1.76
209803_s_at	PHLDA2	pleckstrin homology-like domain, family A, member 2	down	1.48
210191_s_at	PHTF1	putative homeodomain transcription factor 1	up	1.44
215285_s_at	PHTF1	putative homeodomain transcription factor 1	up	1.41
203879_at	PIK3CD	phosphoinositide-3-kinase, catalytic, delta polypeptide	up	1.44
221605_s_at	PIPOX	pipecolic acid oxidase	down	1.38
207469_s_at	PIR	pirin (iron-binding nuclear protein)	up	2.62
219014_at	PLAC8	placenta-specific 8	down	1.51
213222_at	PLCB1	phospholipase C, beta 1 (phosphoinositide-specific)	down	1.59
205934_at	PLCL1	phospholipase C-like 1	down	1.62
228450_at	PLEKHA7	pleckstrin homology domain containing, family A member 7	down	1.40
226122_at	PLEKHG1	pleckstrin homology domain containing, family G (with RhoGef domain) me	up	1.58
201215_at	PLS3	plastin 3	up	1.40
213241_at	PLXNC1	plexin C1	down	1.37
217875_s_at	PMEPA1	prostate transmembrane protein, androgen induced 1	down	1.44
222449_at	PMEPA1	prostate transmembrane protein, androgen induced 1	down	1.52
222450_at	PMEPA1	prostate transmembrane protein, androgen induced 1	down	1.56
1554743_x_at	PMS1	PMS1 postmeiotic segregation increased 1 (S. cerevisiae)	down	1.43
209598_at	PNMA2	paraneoplastic antigen MA2	down	2.18
242455_at	POU3F2	POU class 3 homeobox 2	down	1.44
211341_at	POU4F1	POU class 4 homeobox 1	down	1.36
209529_at	PPAP2C	phosphatidic acid phosphatase type 2C	up	1.96
219195_at	PPARGC1A	peroxisome proliferator-activated receptor gamma, coactivator 1 alpha	down	1.36
212750_at	PPP1R16B	protein phosphatase 1, regulatory (inhibitor) subunit 16B	down	1.62
228494_at	PPP1R9A	protein phosphatase 1, regulatory (inhibitor) subunit 9A	down	1.37
204507_s_at	PPP3R1	protein phosphatase 3, regulatory subunit B, alpha	down	1.36
209685_s_at	PRKCB	protein kinase C, beta	down	1.42
209282_at	PRKD2	protein kinase D2	down	1.41
38269_at	PRKD2	protein kinase D2	down	1.41
202098_s_at	PRMT2	protein arginine methyltransferase 2	up	1.39
226961_at	PRR15	proline rich 15	down	1.37
219168_s_at	PRR5	proline rich 5 (renal)	down	2.25
47069_at	PRR5	proline rich 5 (renal)	down	2.23

216470_x_at	PRSS1 /// PRSS2	protease, serine, 1 (trypsin 1) /// protease, serine, 2 (trypsin 2) /// protease	down	2.14
215395_x_at	PRSS1 /// TRY6	protease, serine, 1 (trypsin 1) /// trypsinogen C	down	1.60
205402_x_at	PRSS2	protease, serine, 2 (trypsin 2)	down	2.25
207341_at	PRTN3	proteinase 3	up	1.78
200866_s_at	PSAP	prosaposin	up	1.44
200871_s_at	PSAP	prosaposin	up	1.37
240091_at	PSMA8	proteasome (prosome, macropain) subunit, alpha type, 8	up	1.70
201067_at	PSMC2	proteasome (prosome, macropain) 26S subunit, ATPase, 2	up	1.40
238020_at	PSMC2	proteasome (prosome, macropain) 26S subunit, ATPase, 2	up	1.65
233314_at	PTEN	phosphatase and tensin homolog	up	1.86
204897_at	PTGER4	prostaglandin E receptor 4 (subtype EP4)	up	1.43
206060_s_at	PTPN22	protein tyrosine phosphatase, non-receptor type 22 (lymphoid)	down	1.48
236539_at	PTPN22	protein tyrosine phosphatase, non-receptor type 22 (lymphoid)	down	1.58
206687_s_at	PTPN6	protein tyrosine phosphatase, non-receptor type 6	down	1.45
204960_at	PTPRCAP	protein tyrosine phosphatase, receptor type, C-associated protein	down	1.47
221840_at	PTPRE	protein tyrosine phosphatase, receptor type, E	up	1.48
204944_at	PTPRG	protein tyrosine phosphatase, receptor type, G	down	1.46
227396_at	PTPRJ	protein tyrosine phosphatase, receptor type, J	up	1.44
203329_at	PTPRM	protein tyrosine phosphatase, receptor type, M	up	1.43
210675_s_at	PTPRR	protein tyrosine phosphatase, receptor type, R	up	1.42
226762_at	PURB	purine-rich element binding protein B	down	1.39
212012_at	PXDN	peroxidasin homolog (Drosophila)	down	1.65
212013_at	PXDN	peroxidasin homolog (Drosophila)	down	1.78
220438_at	QPCTL	glutaminyl-peptide cyclotransferase-like	down	1.41
242414_at	QPRT	quinolinate phosphoribosyltransferase	up	1.40
219622_at	RAB20	RAB20, member RAS oncogene family	down	1.79
209514_s_at	RAB27A	RAB27A, member RAS oncogene family	down	1.37
210951_x_at	RAB27A	RAB27A, member RAS oncogene family	down	1.38
215342_s_at	RABGAP1L	RAB GTPase activating protein 1-like	up	1.48
219151_s_at	RABL2A /// RAE	RAB, member of RAS oncogene family-like 2A /// RAB, member of RAS onco	up	1.36
222742_s_at	RABL5	RAB, member RAS oncogene family-like 5	up	2.03
219494_at	RAD54B	RAD54 homolog B (S. cerevisiae)	down	1.54
213280_at	RAP1GAP2	RAP1 GTPase activating protein 2	up	1.59
49306_at	RASSF4	Ras association (RalGDS/AF-6) domain family member 4	up	1.37
229147_at	RASSF6	Ras association (RalGDS/AF-6) domain family member 6	up	1.70
232549_at	RBM11	RNA binding motif protein 11	up	2.14
205923_at	RELN	reelin	down	1.91
214409_at	RFPL3S	RFPL3 antisense RNA (non-protein coding)	up	1.41
210138_at	RGS20	regulator of G-protein signaling 20	down	1.60
212119_at	RHOQ	ras homolog gene family, member Q	down	1.37
221287_at	RNASEL	ribonuclease L (2',5'-oligoisoadenylate synthetase-dependent)	up	1.36
229285_at	RNASEL	ribonuclease L (2',5'-oligoisoadenylate synthetase-dependent)	up	1.77
213467_at	RND2	Rho family GTPase 2	down	1.38
202636_at	RNF103	ring finger protein 103	down	1.48
242985_x_at	RNF180	ring finger protein 180	down	1.37
235153_at	RNF183	ring finger protein 183	down	1.63
226682_at	RORA	RAR-related orphan receptor A	down	1.92
235567_at	RORA	RAR-related orphan receptor A	down	1.38
225150_s_at	RTKN	rhotekin	up	1.38
213555_at	RWDD2A	RWD domain containing 2A	down	1.44
217728_at	S100A6	S100 calcium binding protein A6	up	2.15
226603_at	SAMD9L	sterile alpha motif domain containing 9-like	up	1.37
214997_at	SCAI	suppressor of cancer cell invasion	down	1.46
228930_at	SCARNA15	Small Cajal body-specific RNA 15	down	1.39
211162_x_at	SCD	stearoyl-CoA desaturase (delta-9-desaturase)	down	1.46
211708_s_at	SCD	stearoyl-CoA desaturase (delta-9-desaturase)	down	1.46

224901_at	SCD5	stearoyl-CoA desaturase 5	down	1.69
218217_at	SCPEP1	serine carboxypeptidase 1	up	1.40
212158_at	SDC2	syndecan 2	up	1.41
202071_at	SDC4	syndecan 4	up	1.55
212451_at	SECISBP2L	SECIS binding protein 2-like	down	1.57
202061_s_at	SEL1L	sel-1 suppressor of lin-12-like (C. elegans)	down	1.37
202062_s_at	SEL1L	sel-1 suppressor of lin-12-like (C. elegans)	down	1.36
202064_s_at	SEL1L	sel-1 suppressor of lin-12-like (C. elegans)	down	1.44
212314_at	SEL1L3	sel-1 suppressor of lin-12-like 3 (C. elegans)	up	1.61
219259_at	SEMA4A	sema domain, immunoglobulin domain (Ig), transmembrane domain (TM) a	down	2.41
234072_at	SEMA4A	sema domain, immunoglobulin domain (Ig), transmembrane domain (TM) a	down	1.95
213169_at	SEMA5A	sema domain, seven thrombospondin repeats (type 1 and type 1-like), tran:	down	1.40
209723_at	SERPINB9	serpin peptidase inhibitor, clade B (ovalbumin), member 9	down	3.41
36545_s_at	SFI1	Sfi1 homolog, spindle assembly associated (yeast)	up	1.37
222258_s_at	SH3BP4	SH3-domain binding protein 4	up	1.42
1557458_s_at	SHB	Src homology 2 domain containing adaptor protein B	up	1.44
233587_s_at	SIPA1L2	signal-induced proliferation-associated 1 like 2	down	1.43
210567_s_at	SKP2	S-phase kinase-associated protein 2 (p45)	down	1.43
219159_s_at	SLAMF7	SLAM family member 7	down	1.99
222838_at	SLAMF7	SLAM family member 7	down	1.99
234306_s_at	SLAMF7	SLAM family member 7	down	1.91
219874_at	SLC12A8	solute carrier family 12 (potassium/chloride transporters), member 8	down	1.40
205856_at	SLC14A1	solute carrier family 14 (urea transporter), member 1 (Kidd blood group)	up	1.67
238029_s_at	SLC16A14	solute carrier family 16, member 14 (monocarboxylic acid transporter 14)	down	2.01
202856_s_at	SLC16A3	solute carrier family 16, member 3 (monocarboxylic acid transporter 4)	down	1.45
209610_s_at	SLC1A4	solute carrier family 1 (glutamate/neutral amino acid transporter), member	down	1.71
209611_s_at	SLC1A4	solute carrier family 1 (glutamate/neutral amino acid transporter), member	down	1.48
212810_s_at	SLC1A4	solute carrier family 1 (glutamate/neutral amino acid transporter), member	down	1.59
212811_x_at	SLC1A4	solute carrier family 1 (glutamate/neutral amino acid transporter), member	down	1.69
222705_s_at	SLC25A15	solute carrier family 25 (mitochondrial carrier; ornithine transporter) meml	down	1.41
230624_at	SLC25A27	solute carrier family 25, member 27	up	1.79
1552774_a_at	SLC25A27	solute carrier family 25, member 27	up	1.42
1554161_at	SLC25A27	solute carrier family 25, member 27	up	1.40
205768_s_at	SLC27A2	solute carrier family 27 (fatty acid transporter), member 2	down	1.95
205769_at	SLC27A2	solute carrier family 27 (fatty acid transporter), member 2	down	1.99
235050_at	SLC2A12	solute carrier family 2 (facilitated glucose transporter), member 12	down	1.36
240799_at	SLC35F4	solute carrier family 35, member F4	up	1.38
230448_at	SLC38A10	solute carrier family 38, member 10	down	1.61
214830_at	SLC38A6	solute carrier family 38, member 6	up	1.39
228486_at	SLC44A1	solute carrier family 44, member 1	down	1.53
232263_at	SLC6A15	solute carrier family 6 (neutral amino acid transporter), member 15	down	1.36
219911_s_at	SLCO4A1	solute carrier organic anion transporter family, member 4A1	down	1.52
222071_s_at	SLCO4C1	solute carrier organic anion transporter family, member 4C1	down	1.36
226743_at	SLFN11	schlafen family member 11	down	1.55
218788_s_at	SMYD3	SET and MYND domain containing 3	up	1.36
213139_at	SNAI2	snail homolog 2 (Drosophila)	up	1.72
218032_at	SNN	stannin	up	1.38
228869_at	SNX20	sorting nexin 20	down	1.49
225728_at	SORBS2	sorbin and SH3 domain containing 2	down	1.84
202935_s_at	SOX9	SRY (sex determining region Y)-box 9	up	1.87
202936_s_at	SOX9	SRY (sex determining region Y)-box 9	up	1.64
207777_s_at	SP140	SP140 nuclear body protein	down	1.44
234995_at	SPICE1	spindle and centriole associated protein 1	down	1.40
210715_s_at	SPINT2	serine peptidase inhibitor, Kunitz type, 2	down	1.50
204011_at	SPRY2	sprouty homolog 2 (Drosophila)	down	1.38
213562_s_at	SQLE	squalene epoxidase	down	1.36

213577_at	SQLE	squalene epoxidase	down	1.36
201471_s_at	SQSTM1	sequestosome 1	up	1.62
213112_s_at	SQSTM1	sequestosome 1	up	1.64
244804_at	SQSTM1	sequestosome 1	up	1.47
225252_at	SRXN1	sulfiredoxin 1	up	1.40
217790_s_at	SSR3	signal sequence receptor, gamma (translocon-associated protein gamma)	down	1.44
210942_s_at	ST3GAL6	ST3 beta-galactoside alpha-2,3-sialyltransferase 6	down	1.38
213355_at	ST3GAL6	ST3 beta-galactoside alpha-2,3-sialyltransferase 6	down	1.58
201998_at	ST6GAL1	ST6 beta-galactosamide alpha-2,6-sialyltranferase 1	down	1.40
214971_s_at	ST6GAL1	ST6 beta-galactosamide alpha-2,6-sialyltranferase 1	down	1.42
228821_at	ST6GAL2	ST6 beta-galactosamide alpha-2,6-sialyltranferase 2	down	2.53
1555123_at	ST6GAL2	ST6 beta-galactosamide alpha-2,6-sialyltranferase 2	down	1.63
226390_at	STARD4	StAR-related lipid transfer (START) domain containing 4	down	1.38
217503_at	STK17B	serine/threonine kinase 17b	down	1.45
203767_s_at	STS	steroid sulfatase (microsomal), isozyme S	down	1.51
224724_at	SULF2	sulfatase 2	down	1.46
233555_s_at	SULF2	sulfatase 2	down	1.47
206546_at	SYCP2	synaptonemal complex protein 2	up	1.82
201463_s_at	TALDO1	transaldolase 1	up	1.58
227611_at	TARSL2	threonyl-tRNA synthetase-like 2	down	1.42
213912_at	TBC1D30	TBC1 domain family, member 30	down	1.50
213913_s_at	TBC1D30	TBC1 domain family, member 30	down	1.42
227279_at	TCEAL3	transcription elongation factor A (SII)-like 3	down	1.77
228837_at	TCF4	transcription factor 4	up	1.43
205943_at	TDO2	tryptophan 2,3-dioxygenase	up	2.89
232692_at	TDRD6	tudor domain containing 6	up	1.52
228906_at	TET1	tet oncogene 1	down	1.58
221035_s_at	TEX14	testis expressed 14	down	1.81
226157_at	TFDP2	transcription factor Dp-2 (E2F dimerization partner 2)	up	1.50
237346_at	TGDS	TDP-glucose 4,6-dehydratase	down	1.39
205015_s_at	TGFA	transforming growth factor, alpha	down	1.43
205016_at	TGFA	transforming growth factor, alpha	down	2.24
1566901_at	TGIF1	TGFB-induced factor homeobox 1	up	1.38
203167_at	TIMP2	TIMP metallopeptidase inhibitor 2	down	1.37
1552360_a_at	TIRAP	toll-interleukin 1 receptor (TIR) domain containing adaptor protein	up	1.36
202011_at	TJP1	tight junction protein 1 (zona occludens 1)	down	1.65
214168_s_at	TJP1	tight junction protein 1 (zona occludens 1)	down	1.41
212769_at	TLE3	transducin-like enhancer of split 3 (E(sp1) homolog, Drosophila)	down	1.37
220532_s_at	TMEM176B	transmembrane protein 176B	down	1.40
230467_at	TMEM52	transmembrane protein 52	down	1.38
241364_at	TMEM57	transmembrane protein 57	up	1.52
207638_at	TMPRSS15	transmembrane protease, serine 15	down	1.49
226322_at	TMTC1	transmembrane and tetratricopeptide repeat containing 1	up	1.58
226931_at	TMTC1	transmembrane and tetratricopeptide repeat containing 1	up	1.63
1552822_at	TMX3	thioredoxin-related transmembrane protein 3	down	1.38
210260_s_at	TNFAIP8	tumor necrosis factor, alpha-induced protein 8	down	1.40
203671_at	TPMT	thiopurine S-methyltransferase	up	1.48
217147_s_at	TRAT1	T cell receptor associated transmembrane adaptor 1	down	1.94
210705_s_at	TRIM5	tripartite motif-containing 5	up	1.65
210055_at	TSHR	thyroid stimulating hormone receptor	down	1.39
215442_s_at	TSHR	thyroid stimulating hormone receptor	down	1.40
215443_at	TSHR	thyroid stimulating hormone receptor	down	1.37
217979_at	TSPAN13	tetraspanin 13	down	1.64
227307_at	TSPAN18	tetraspanin 18	up	2.12
225775_at	TSPAN33	tetraspanin 33	down	1.39
213122_at	TSPYL5	TSPY-like 5	down	1.41

229170_s_at	TTC18	tetratricopeptide repeat domain 18	up	1.43
210652_s_at	TTC39A	tetratricopeptide repeat domain 39A	up	1.51
228724_at	TTLL7	tubulin tyrosine ligase-like family, member 7	up	1.40
235561_at	TXNL1	thioredoxin-like 1	up	1.83
214755_at	UAP1L1	UDP-N-acteylglucosamine pyrophosphorylase 1-like 1	down	1.43
1555834_at	UCHL1	Ubiquitin carboxyl-terminal esterase L1 (ubiquitin thiolesterase)	up	1.37
235749_at	UGGT2	UDP-glucose glycoprotein glucosyltransferase 2	down	1.36
219740_at	VASH2	vasohibin 2	up	2.27
235343_at	VASH2	vasohibin 2	up	2.80
218807_at	VAV3	vav 3 guanine nucleotide exchange factor	down	1.44
205506_at	VIL1	villin 1	down	2.12
228912_at	VIL1	villin 1	down	1.54
209822_s_at	VLDLR	very low density lipoprotein receptor	down	1.43
220917_s_at	WDR19	WD repeat domain 19	up	1.40
231251_at	WIPF2	WAS/WASL interacting protein family, member 2	down	1.41
229849_at	WIPF3	WAS/WASL interacting protein family, member 3	up	1.57
203827_at	WIPI1	WD repeat domain, phosphoinositide interacting 1	down	1.59
213836_s_at	WIPI1	WD repeat domain, phosphoinositide interacting 1	down	1.55
221958_s_at	WLS	wntless homolog (Drosophila)	down	1.52
228950_s_at	WLS	wntless homolog (Drosophila)	down	1.50
228617_at	XAF1	XIAP associated factor 1	down	1.52
213725_x_at	XYLT1	xylosyltransferase I	down	2.24
232574_at	XYLT1	xylosyltransferase I	down	1.37
227020_at	YPEL2	yippee-like 2 (Drosophila)	down	1.44
224503_s_at	ZCCHC2	zinc finger, CCHC domain containing 2	up	1.37
228637_at	ZDHHC1	zinc finger, DHHC-type containing 1	up	1.39
203603_s_at	ZEB2	zinc finger E-box binding homeobox 2	up	1.37
235366_at	ZNF10	zinc finger protein 10	up	1.39
243312_at	ZNF107	Zinc finger protein 107	up	1.38
206683_at	ZNF165	zinc finger protein 165	down	1.70
206175_x_at	ZNF222	zinc finger protein 222	up	1.43
1558888_x_at	ZNF321	zinc finger protein 321	up	1.36
244007_at	ZNF462	zinc finger protein 462	up	1.40
1552794_a_at	ZNF547	zinc finger protein 547	up	1.40
243790_at	ZNF585A	zinc finger protein 585A	up	1.37
207781_s_at	ZNF711	zinc finger protein 711	down	1.49
228988_at	ZNF711	zinc finger protein 711	down	1.54
229732_at	ZNF823	zinc finger protein 823	up	1.39
244640_at	ZNF850	zinc finger protein 850	up	1.51
230421_at	ZNF879	zinc finger protein 879	up	1.41
200808_s_at	ZYX	zyxin	up	1.38
215059_at			up	1.37
215386_at			up	1.48
220467_at			down	1.37
222111_at			down	1.41
226348_at			down	1.39
226560_at			down	1.46
227591_at			up	1.42
227755_at			down	1.42
228390_at			down	1.53
228643_at			down	1.37
229072_at			down	1.70
229298_at			down	1.39
229423_at			up	1.46
230319_at			up	1.42
230499_at			up	1.46

230795_at	down	1.51
230913_at	up	1.48
231040_at	up	1.39
231644_at	down	1.43
233401_at	up	1.50
234562_x_at	up	1.40
235251_at	up	1.47
235456_at	down	1.49
235738_at	down	1.36
235875_at	down	1.80
236198_at	up	1.67
236451_at	down	2.33
236513_at	down	1.42
236520_at	up	1.46
236598_at	down	1.52
238091_at	down	1.52
238668_at	up	1.40
238735_at	down	1.49
238946_at	down	1.46
238953_at	up	1.53
239784_at	up	1.43
240121_x_at	up	1.41
240574_at	down	1.49
241356_at	up	1.37
241394_at	up	1.48
242384_at	down	1.45
242723_at	down	1.42
242798_at	down	1.41
243134_at	down	1.41
243154_at	down	1.85
243366_s_at	down	1.36
243489_at	up	1.40
243543_at	down	1.42
243810_at	down	1.48
244139_s_at	down	1.40
244700_at	down	1.44
1556111_s_at	down	1.37
1556261_a_at	up	1.50
1557139_at	up	1.43
1557149_at	up	1.54
1557383_a_at	down	1.41
1558401_at	down	1.50
1558710_at	down	1.56
1558795_at	down	1.58
1558937_s_at	down	1.39
1559007_s_at	up	1.37
1560738_at	down	1.42
1562529_s_at	down	1.50
1564424_at	down	1.46

Supplementary Table S1. Differentially expressed genes in KMS-34/Cfz versus KMS-34

Probe Set ID	Symbol	Name	Change	FC > 1.5
210852_s_at	AASS	aminoadipate-semialdehyde synthase	up	1.82
209993_at	ABCB1	ATP-binding cassette, sub-family B (MDR/TAP), member 1	up	3.99
209994_s_at	ABCB1 /// ABCE	ATP-binding cassette, sub-family B (MDR/TAP), member 1 /// ATP-binding c	up	3.25
207623_at	ABCF2	ATP-binding cassette, sub-family F (GCN20), member 2	down	1.71
210006_at	ABHD14A	abhydrolase domain containing 14A	down	1.53
226893_at	ABL2	v-abl Abelson murine leukemia viral oncogene homolog 2	down	1.50
228132_at	ABLIM2	actin binding LIM protein family, member 2	up	1.72
236514_at	ACOT8	acyl-CoA thioesterase 8	up	1.90
228603_at	ACTR3	ARP3 actin-related protein 3 homolog (yeast)	up	1.69
223874_at	ACTR3C	ARP3 actin-related protein 3 homolog C (yeast)	up	1.69
203935_at	ACVR1	activin A receptor, type I	up	1.55
213808_at	ADAM23	ADAM metallopeptidase domain 23	up	1.64
201752_s_at	ADD3	adducin 3 (gamma)	up	1.54
201753_s_at	ADD3	adducin 3 (gamma)	up	1.55
205882_x_at	ADD3	adducin 3 (gamma)	up	1.51
217729_s_at	AES	amino-terminal enhancer of split	down	1.58
223779_at	AFAP1-AS	AFAP1 antisense RNA (non-protein coding)	up	2.05
231299_at	AGAP3	ArfGAP with GTPase domain, ankyrin repeat and PH domain 3	up	1.71
239026_x_at	AGAP3	ArfGAP with GTPase domain, ankyrin repeat and PH domain 3	up	1.55
232395_x_at	AGBL3	ATP/GTP binding protein-like 3	up	1.86
226665_at	AHSA2	AHA1, activator of heat shock 90kDa protein ATPase homolog 2 (yeast)	up	1.54
215789_s_at	AJAP1	adherens junctions associated protein 1	down	1.64
204348_s_at	AK4	adenylate kinase 4	down	1.72
225342_at	AK4	adenylate kinase 4	down	1.87
227530_at	AKAP12	A kinase (PRKA) anchor protein 12	up	1.54
223143_s_at	AKIRIN2	akirin 2	up	1.66
223144_s_at	AKIRIN2	akirin 2	up	1.61
223145_s_at	AKIRIN2	akirin 2	up	1.51
202022_at	ALDOC	aldolase C, fructose-bisphosphate	down	1.53
242900_at	ALG10B	asparagine-linked glycosylation 10, alpha-1,2-glucosyltransferase homolog I	up	1.55
204174_at	ALOX5AP	arachidonate 5-lipoxygenase-activating protein	up	2.31
229596_at	AMDHD1	amidohydrolase domain containing 1	down	1.59
206385_s_at	ANK3	ankyrin 3, node of Ranvier (ankyrin G)	up	1.93
1559640_at	ANKFN1	Ankyrin-repeat and fibronectin type III domain containing 1	up	1.61
226663_at	ANKRD10	ankyrin repeat domain 10	up	1.55
204671_s_at	ANKRD6	ankyrin repeat domain 6	up	1.83
206112_at	ANKRD7	ankyrin repeat domain 7	up	1.81
201012_at	ANXA1	annexin A1	up	1.66
201301_s_at	ANXA4	annexin A4	up	1.96
201302_at	ANXA4	annexin A4	up	1.82
1557236_at	APOL6	apolipoprotein L, 6	up	1.64
225166_at	ARHGAP18	Rho GTPase activating protein 18	up	1.72
225173_at	ARHGAP18	Rho GTPase activating protein 18	up	1.56
233849_s_at	ARHGAP5	Rho GTPase activating protein 5	up	1.54
201335_s_at	ARHGEF12	Rho guanine nucleotide exchange factor (GEF) 12	up	1.74
227197_at	ARHGEF26	Rho guanine nucleotide exchange factor (GEF) 26	up	1.94
58780_s_at	ARHGEF40	Rho guanine nucleotide exchange factor (GEF) 40	up	2.10
213138_at	ARID5A	AT rich interactive domain 5A (MRF1-like)	up	1.67
220658_s_at	ARNTL2	aryl hydrocarbon receptor nuclear translocator-like 2	up	2.41
224204_x_at	ARNTL2	aryl hydrocarbon receptor nuclear translocator-like 2	up	1.70
206414_s_at	ASAP2	ArfGAP with SH3 domain, ankyrin repeat and PH domain 2	up	2.03
232838_at	ASXL3	additional sex combs like 3 (Drosophila)	up	1.94
233536_at	ASXL3	additional sex combs like 3 (Drosophila)	up	2.38

201242_s_at	ATP1B1	ATPase, Na+/K+ transporting, beta 1 polypeptide	up	1.87
201243_s_at	ATP1B1	ATPase, Na+/K+ transporting, beta 1 polypeptide	up	1.70
217867_x_at	BACE2	beta-site APP-cleaving enzyme 2	down	1.65
222446_s_at	BACE2	beta-site APP-cleaving enzyme 2	down	1.72
221234_s_at	BACH2	BTB and CNC homology 1, basic leucine zipper transcription factor 2	up	1.70
227173_s_at	BACH2	BTB and CNC homology 1, basic leucine zipper transcription factor 2	up	1.59
236796_at	BACH2	BTB and CNC homology 1, basic leucine zipper transcription factor 2	up	1.74
219667_s_at	BANK1	B-cell scaffold protein with ankyrin repeats 1	down	2.49
222915_s_at	BANK1	B-cell scaffold protein with ankyrin repeats 1	down	1.87
1558662_s_at	BANK1	B-cell scaffold protein with ankyrin repeats 1	down	1.82
203080_s_at	BAZ2B	bromodomain adjacent to zinc finger domain, 2B	up	1.56
223227_at	BBS2	Bardet-Biedl syndrome 2	up	1.55
214452_at	BCAT1	branched chain amino-acid transaminase 1, cytosolic	up	2.44
225285_at	BCAT1	branched chain amino-acid transaminase 1, cytosolic	up	3.13
226517_at	BCAT1	branched chain amino-acid transaminase 1, cytosolic	up	4.41
227896_at	BCCIP	BRCA2 and CDKN1A interacting protein	down	1.54
203685_at	BCL2	B-cell CLL/lymphoma 2	up	1.65
211715_s_at	BDH1	3-hydroxybutyrate dehydrogenase, type 1	down	1.82
206956_at	BGLAP /// PMF	bone gamma-carboxyglutamate (gla) protein /// polyamine-modulated fact	down	1.63
228636_at	BHLHE22	basic helix-loop-helix family, member e22	up	1.57
223185_s_at	BHLHE41	basic helix-loop-helix family, member e41	down	1.50
1554020_at	BICD1	bicaudal D homolog 1 (Drosophila)	up	1.54
219546_at	BMP2K	BMP2 inducible kinase	up	1.57
59644_at	BMP2K	BMP2 inducible kinase	up	1.55
243829_at	BRAF	v-raf murine sarcoma viral oncogene homolog B1	up	1.51
202946_s_at	BTBD3	BTB (POZ) domain containing 3	up	2.40
214117_s_at	BTD	biotinidase	down	1.59
228434_at	BTNL9	butyrophilin-like 9	down	1.54
205839_s_at	BZRAP1	benzodiazapine receptor (peripheral) associated protein 1	up	2.48
220560_at	C11orf21	chromosome 11 open reading frame 21	up	1.50
236646_at	C12orf59	chromosome 12 open reading frame 59	down	2.43
209574_s_at	C18orf1	chromosome 18 open reading frame 1	up	1.53
230033_at	C19orf51	chromosome 19 open reading frame 51	down	2.98
230256_at	C1orf104	Chromosome 1 open reading frame 104	down	1.52
219010_at	C1orf106	chromosome 1 open reading frame 106	down	10.85
1558508_a_at	C1orf53	chromosome 1 open reading frame 53	down	1.59
225401_at	C1orf85	chromosome 1 open reading frame 85	down	1.83
1558693_s_at	C1orf85	chromosome 1 open reading frame 85	down	1.53
212067_s_at	C1R	complement component 1, r subcomponent	down	1.51
219463_at	C20orf103	chromosome 20 open reading frame 103	down	1.74
224690_at	C20orf108	chromosome 20 open reading frame 108	up	1.56
225224_at	C20orf112	chromosome 20 open reading frame 112	up	1.60
233829_at	C20orf118	chromosome 20 open reading frame 118	up	1.55
223157_at	C4orf14	chromosome 4 open reading frame 14	down	1.54
219450_at	C4orf19	chromosome 4 open reading frame 19	up	1.93
235350_at	C4orf19	chromosome 4 open reading frame 19	up	1.75
219747_at	C4orf31	chromosome 4 open reading frame 31	down	1.63
220770_s_at	C5orf54	chromosome 5 open reading frame 54	up	1.61
207963_at	C6orf54	chromosome 6 open reading frame 54	up	1.52
237592_at	C6orf94	chromosome 6 open reading frame 94	down	1.80
229146_at	C7orf31	chromosome 7 open reading frame 31	up	1.58
220032_at	C7orf58	chromosome 7 open reading frame 58	up	2.17
228728_at	C7orf58	chromosome 7 open reading frame 58	up	2.06
209726_at	CA11	carbonic anhydrase XI	down	1.83
220234_at	CA8	carbonic anhydrase VIII	up	1.59
206331_at	CALCRL	calcitonin receptor-like	up	1.77

210815_s_at	CALCRL	calcitonin receptor-like	up	2.07
212765_at	CAMSAP1L1	calmodulin regulated spectrin-associated protein 1-like 1	down	1.79
217196_s_at	CAMSAP1L1	calmodulin regulated spectrin-associated protein 1-like 1	down	1.61
208683_at	CAPN2	calpain 2, (m/II) large subunit	down	6.64
1552703_s_at	CARD16 /// CAS	caspase recruitment domain family, member 16 /// caspase 1, apoptosis-re	up	1.78
206011_at	CASP1	caspase 1, apoptosis-related cysteine peptidase (interleukin 1, beta, conver	up	1.58
209970_x_at	CASP1	caspase 1, apoptosis-related cysteine peptidase (interleukin 1, beta, conver	up	1.58
211366_x_at	CASP1	caspase 1, apoptosis-related cysteine peptidase (interleukin 1, beta, conver	up	1.66
211367_s_at	CASP1	caspase 1, apoptosis-related cysteine peptidase (interleukin 1, beta, conver	up	1.72
211368_s_at	CASP1	caspase 1, apoptosis-related cysteine peptidase (interleukin 1, beta, conver	up	1.79
203323_at	CAV2	caveolin 2	down	1.51
209145_s_at	CBFA2T2	core-binding factor, runt domain, alpha subunit 2; translocated to, 2	up	1.58
238549_at	CBFA2T2	core-binding factor, runt domain, alpha subunit 2; translocated to, 2	up	1.69
209682_at	CBLB	Cas-Br-M (murine) ecotropic retroviral transforming sequence b	up	1.53
205379_at	CBR3	carbonyl reductase 3	up	1.54
230900_at	CCDC110	coiled-coil domain containing 110	down	1.55
1554216_at	CCDC132	coiled-coil domain containing 132	up	1.54
1554217_a_at	CCDC132	coiled-coil domain containing 132	up	1.58
226972_s_at	CCDC136	coiled-coil domain containing 136	up	1.84
236745_at	CCDC78	coiled-coil domain containing 78	up	1.60
216598_s_at	CCL2	chemokine (C-C motif) ligand 2	up	6.55
1405_i_at	CCL5	chemokine (C-C motif) ligand 5	up	1.58
208711_s_at	CCND1	cyclin D1	down	2.75
208712_at	CCND1	cyclin D1	down	6.15
222156_x_at	CCPG1	cell cycle progression 1	up	1.58
206587_at	CCT6B	chaperonin containing TCP1, subunit 6B (zeta 2)	up	1.53
201925_s_at	CD55	CD55 molecule, decay accelerating factor for complement (Cromer blood gı	down	1.60
201926_s_at	CD55	CD55 molecule, decay accelerating factor for complement (Cromer blood gı	down	1.57
1555950_a_at	CD55	CD55 molecule, decay accelerating factor for complement (Cromer blood gı	down	1.57
202910_s_at	CD97	CD97 molecule (CD55 ligand)	down	1.66
208022_s_at	CDC14B	CDC14 cell division cycle 14 homolog B (S. cerevisiae)	up	2.18
221555_x_at	CDC14B	CDC14 cell division cycle 14 homolog B (S. cerevisiae)	up	1.77
221556_at	CDC14B	CDC14 cell division cycle 14 homolog B (S. cerevisiae)	up	4.17
204693_at	CDC42EP1	CDC42 effector protein (Rho GTPase binding) 1	down	3.54
214721_x_at	CDC42EP4	CDC42 effector protein (Rho GTPase binding) 4	down	1.50
218157_x_at	CDC42SE1	CDC42 small effector 1	down	1.60
229120_s_at	CDC42SE1	CDC42 small effector 1	down	1.62
204995_at	CDK5R1	cyclin-dependent kinase 5, regulatory subunit 1 (p35)	up	1.51
203973_s_at	CEBPD	CCAAT/enhancer binding protein (C/EBP), delta	down	1.50
218421_at	CERK	ceramide kinase	down	1.51
203166_at	CFDP1	craniofacial development protein 1	up	1.52
215388_s_at	CFH /// CFHR1	complement factor H /// complement factor H-related 1	up	1.69
207486_x_at	CHN2	chimerin (chimaerin) 2	up	1.98
211419_s_at	CHN2	chimerin (chimaerin) 2	up	2.74
213385_at	CHN2	chimerin (chimaerin) 2	up	3.12
242488_at	CHRM3	cholinergic receptor, muscarinic 3	down	1.92
1553705_a_at	CHRM3	cholinergic receptor, muscarinic 3	down	1.51
1559633_a_at	CHRM3	cholinergic receptor, muscarinic 3	down	2.35
239146_at	CLDND1	claudin domain containing 1	down	1.60
233500_x_at	CLEC2D	C-type lectin domain family 2, member D	up	1.69
213317_at	CLIC5	chloride intracellular channel 5	up	3.26
217628_at	CLIC5	chloride intracellular channel 5	up	1.58
219944_at	CLIP4	CAP-GLY domain containing linker protein family, member 4	up	2.26
226425_at	CLIP4	CAP-GLY domain containing linker protein family, member 4	up	2.42
1554677_s_at	CMTM4	CKLF-like MARVEL transmembrane domain containing 4	up	1.53
201445_at	CNN3	calponin 3, acidic	up	1.65

227209_at	CNTN1	Contactin 1	down	1.73
244632_at	CNTN5	Contactin 5	up	1.60
213110_s_at	COL4A5	collagen, type IV, alpha 5	down	1.82
205081_at	CRIP1	cysteine-rich protein 1 (intestinal)	down	1.70
219049_at	CSGALNACT1	chondroitin sulfate N-acetylgalactosaminyltransferase 1	up	2.05
201218_at	CTBP2	C-terminal binding protein 2	down	1.98
210554_s_at	CTBP2	C-terminal binding protein 2	down	1.55
210835_s_at	CTBP2	C-terminal binding protein 2	down	1.53
225681_at	CTHRC1	collagen triple helix repeat containing 1	up	2.03
213274_s_at	CTSB	cathepsin B	up	1.52
202295_s_at	CTSH	cathepsin H	down	1.76
202902_s_at	CTSS	cathepsin S	down	1.52
218097_s_at	CUEDC2	CUE domain containing 2	down	1.59
205898_at	CX3CR1	chemokine (C-X3-C motif) receptor 1	up	2.35
209687_at	CXCL12	chemokine (C-X-C motif) ligand 12	up	1.60
209201_x_at	CXCR4	chemokine (C-X-C motif) receptor 4	up	1.52
211919_s_at	CXCR4	chemokine (C-X-C motif) receptor 4	up	1.51
217028_at	CXCR4	chemokine (C-X-C motif) receptor 4	up	1.58
232087_at	CXorf23	chromosome X open reading frame 23	up	1.50
227382_at	CYB5B	cytochrome b5 type B (outer mitochondrial membrane)	up	1.76
238554_at	CYB5B	cytochrome b5 type B (outer mitochondrial membrane)	up	1.72
226833_at	CYB5D1	cytochrome b5 domain containing 1	down	1.50
206424_at	CYP26A1	cytochrome P450, family 26, subfamily A, polypeptide 1	down	2.16
231747_at	CYSLTR1	cysteinyl leukotriene receptor 1	down	1.90
216060_s_at	DAAM1	dishevelled associated activator of morphogenesis 1	up	1.54
205337_at	DCT	dopachrome tautomerase (dopachrome delta-isomerase, tyrosine-related	up	2.02
205338_s_at	DCT	dopachrome tautomerase (dopachrome delta-isomerase, tyrosine-related	up	1.58
209094_at	DDAH1	dimethylarginine dimethylaminohydrolase 1	up	1.93
220004_at	DDX43	DEAD (Asp-Glu-Ala-Asp) box polypeptide 43	up	2.05
1568815_a_at	DDX50	DEAD (Asp-Glu-Ala-Asp) box polypeptide 50	down	1.50
219696_at	DENND1B	DENN/MADD domain containing 1B	down	1.55
228032_s_at	DENND1B	DENN/MADD domain containing 1B	down	1.67
238787_at	DENND1B	DENN/MADD domain containing 1B	down	1.54
47553_at	DFNB31	deafness, autosomal recessive 31	up	1.52
226064_s_at	DGAT2	diacylglycerol O-acyltransferase 2	down	1.81
229579_s_at	DISP2	dispatched homolog 2 (Drosophila)	up	1.62
230426_at	DLD	dihydrolipoamide dehydrogenase	up	1.57
226994_at	DNAJA2	DnaJ (Hsp40) homolog, subfamily A, member 2	up	1.50
215116_s_at	DNM1	dynamin 1	up	1.71
220668_s_at	DNMT3B	DNA (cytosine-5-)-methyltransferase 3 beta	up	1.51
205003_at	DOCK4	dedicator of cytokinesis 4	up	1.70
236442_at	DPF3	D4, zinc and double PHD fingers, family 3	down	2.04
238532_at	DPF3	D4, zinc and double PHD fingers, family 3	down	1.82
215143_at	DPY19L2P2	dpy-19-like 2 pseudogene 2 (C. elegans)	up	1.51
1554534_at	DPYD	dihydropyrimidine dehydrogenase	up	2.09
1554536_at	DPYD	dihydropyrimidine dehydrogenase	up	2.30
218627_at	DRAM1	DNA-damage regulated autophagy modulator 1	up	1.53
210091_s_at	DTNA	dystrobrevin, alpha	up	1.51
225415_at	DTX3L	deltex 3-like (Drosophila)	up	2.11
208891_at	DUSP6	dual specificity phosphatase 6	up	1.97
208892_s_at	DUSP6	dual specificity phosphatase 6	up	1.87
208893_s_at	DUSP6	dual specificity phosphatase 6	up	1.68
202971_s_at	DYRK2	dual-specificity tyrosine-(Y)-phosphorylation regulated kinase 2	up	1.50
219551_at	EAF2	ELL associated factor 2	down	1.89
1568672_at	EAF2	ELL associated factor 2	down	2.01
1568673_s_at	EAF2	ELL associated factor 2	down	2.10

227103_s_at	ECE2	endothelin converting enzyme 2	down	1.72
220342_x_at	EDEM3	ER degradation enhancer, mannosidase alpha-like 3	down	1.73
220926_s_at	EDEM3	ER degradation enhancer, mannosidase alpha-like 3	down	1.55
223243_s_at	EDEM3	ER degradation enhancer, mannosidase alpha-like 3	down	1.66
225885_at	EEA1	early endosome antigen 1	up	1.59
201694_s_at	EGR1	early growth response 1	up	1.63
227404_s_at	EGR1	Early growth response 1	up	2.34
227930_at	EIF2C4	Eukaryotic translation initiation factor 2C, 4	up	1.81
1554309_at	EIF4G3	eukaryotic translation initiation factor 4 gamma, 3	up	1.63
31845_at	ELF4	E74-like factor 4 (ets domain transcription factor)	up	2.10
225159_s_at	ELK4	ELK4, ETS-domain protein (SRF accessory protein 1)	down	1.51
231930_at	ELMOD1	ELMO/CED-12 domain containing 1	down	1.50
213779_at	EMID1	EMI domain containing 1	up	1.66
204975_at	EMP2	epithelial membrane protein 2	down	1.75
225078_at	EMP2	epithelial membrane protein 2	down	1.81
225079_at	EMP2	epithelial membrane protein 2	down	2.21
1553672_at	ENAH	enabled homolog (Drosophila)	down	1.59
212573_at	ENDOD1	endonuclease domain containing 1	up	1.68
212336_at	EPB41L1	erythrocyte membrane protein band 4.1-like 1	up	1.54
202017_at	EPHX1	epoxide hydrolase 1, microsomal (xenobiotic)	down	1.75
227609_at	EPSTI1	epithelial stromal interaction 1 (breast)	up	2.00
235276_at	EPSTI1	epithelial stromal interaction 1 (breast)	up	2.03
230183_at	EXT1	exostosin 1	up	1.62
228298_at	FAM113B	family with sequence similarity 113, member B	up	1.73
212979_s_at	FAM115A	family with sequence similarity 115, member A	up	1.57
212981_s_at	FAM115A	family with sequence similarity 115, member A	up	1.59
229512_at	FAM120C	family with sequence similarity 120C	up	1.73
217966_s_at	FAM129A	family with sequence similarity 129, member A	up	2.23
217967_s_at	FAM129A	family with sequence similarity 129, member A	up	2.53
203550_s_at	FAM189B	family with sequence similarity 189, member B	down	1.57
229762_at	FAM200A	Family with sequence similarity 200, member A	up	1.50
231146_at	FAM24B	family with sequence similarity 24, member B	down	1.61
236316_at	FAM3C	family with sequence similarity 3, member C	up	1.69
221766_s_at	FAM46A	family with sequence similarity 46, member A	up	1.95
220306_at	FAM46C	family with sequence similarity 46, member C	down	1.64
226811_at	FAM46C	family with sequence similarity 46, member C	down	1.51
203986_at	FAM47E /// STE	family with sequence similarity 47, member E /// starch binding domain 1	down	1.53
208092_s_at	FAM49A	family with sequence similarity 49, member A	up	1.69
219895_at	FAM70A	family with sequence similarity 70, member A	up	2.90
1568609_s_at	FAM91A2 /// FI	family with sequence similarity 91, member A2 /// hypothetical FLJ39739 /,	up	1.60
228427_at	FBXO16	F-box protein 16	up	1.53
209209_s_at	FERMT2	fermitin family member 2	up	1.65
227948_at	FGD4	FYVE, RhoGEF and PH domain containing 4	up	1.64
219522_at	FJX1	four jointed box 1 (Drosophila)	down	1.83
1562904_s_at	FLJ10661	family with sequence similarity 86, member A pseudogene	up	1.71
235291_s_at	FLJ32255	hypothetical LOC643977	up	1.68
235292_at	FLJ32255	hypothetical LOC643977	up	1.82
227883_at	FLJ36031	hypothetical protein FLJ36031	up	1.55
239657_x_at	FOXO6	forkhead box O6	up	1.71
243278_at	FOXP2	forkhead box P2	up	1.52
1555352_at	FOXP2	forkhead box P2	up	1.57
1555516_at	FOXP2	forkhead box P2	up	1.51
204072_s_at	FRY	furry homolog (Drosophila)	down	1.79
238551_at	FUT11	fucosyltransferase 11 (alpha (1,3) fucosyltransferase)	down	1.58
212486_s_at	FYN	FYN oncogene related to SRC, FGR, YES	up	1.61
210220_at	FZD2	frizzled homolog 2 (Drosophila)	down	1.63

208868_s_at	GABARAPL1	GABA(A) receptor-associated protein like 1	down	1.56
208869_s_at	GABARAPL1	GABA(A) receptor-associated protein like 1	down	1.58
211458_s_at	GABARAPL1 ///	GABA(A) receptor-associated protein like 1 /// GABA(A) receptors associate	down	1.63
203725_at	GADD45A	growth arrest and DNA-damage-inducible, alpha	up	1.52
219271_at	GALNT14	UDP-N-acetyl-alpha-D-galactosamine:polypeptide N-acetylgalactosaminyltr	down	1.51
224741_x_at	GAS5	growth arrest-specific 5 (non-protein coding)	down	1.51
227517_s_at	GAS5	growth arrest-specific 5 (non-protein coding)	down	1.53
228238_at	GAS5	growth arrest-specific 5 (non-protein coding)	down	1.59
203178_at	GATM	glycine amidinotransferase (L-arginine:glycine amidinotransferase)	down	1.83
216733_s_at	GATM	glycine amidinotransferase (L-arginine:glycine amidinotransferase)	down	1.83
202269_x_at	GBP1	guanylate binding protein 1, interferon-inducible, 67kDa	up	1.58
202270_at	GBP1	guanylate binding protein 1, interferon-inducible, 67kDa	up	1.56
231577_s_at	GBP1	guanylate binding protein 1, interferon-inducible, 67kDa	up	1.94
239761_at	GCNT1	glucosaminyl (N-acetyl) transferase 1, core 2	up	1.53
221279_at	GDAP1	ganglioside-induced differentiation-associated protein 1	up	1.56
226269_at	GDAP1	ganglioside-induced differentiation-associated protein 1	up	1.86
226271_at	GDAP1	ganglioside-induced differentiation-associated protein 1	up	1.62
226136_at	GLIPR1	GLI pathogenesis-related 1	up	1.55
200648_s_at	GLUL	glutamate-ammonia ligase	up	1.61
215001_s_at	GLUL	glutamate-ammonia ligase	up	1.54
217202_s_at	GLUL	glutamate-ammonia ligase	up	1.55
204993_at	GNAZ	guanine nucleotide binding protein (G protein), alpha z polypeptide	up	1.51
206896_s_at	GNG7	guanine nucleotide binding protein (G protein), gamma 7	down	1.53
212959_s_at	GNPTAB	N-acetylglucosamine-1-phosphate transferase, alpha and beta subunits	up	1.66
219078_at	GPATCH2	G patch domain containing 2	down	1.91
1556126_s_at	GPATCH2	G patch domain containing 2	down	1.60
204983_s_at	GPC4	glypican 4	down	1.82
204984_at	GPC4	glypican 4	down	1.81
225447_at	GPD2	glycerol-3-phosphate dehydrogenase 2 (mitochondrial)	up	1.84
210473_s_at	GPR125	G protein-coupled receptor 125	down	1.52
232267_at	GPR133	G protein-coupled receptor 133	down	1.89
203817_at	GUCY1B3	guanylate cyclase 1, soluble, beta 3	down	1.52
227471_at	HACE1	HECT domain and ankyrin repeat containing, E3 ubiquitin protein ligase 1	up	1.53
206834_at	HBD	hemoglobin, delta	down	2.37
206106_at	HDAC10 /// LO(	histone deacetylase 10 /// mitogen-activated protein kinase 12-like /// mit(	up	1.63
209524_at	HDGFRP3	hepatoma-derived growth factor, related protein 3	up	1.53
209526_s_at	HDGFRP3	hepatoma-derived growth factor, related protein 3	up	1.56
1552628_a_at	HERPUD2	HERPUD family member 2	up	1.57
218839_at	HEY1	hairy/enhancer-of-split related with YRPW motif 1	up	1.52
44783_s_at	HEY1	hairy/enhancer-of-split related with YRPW motif 1	up	1.69
209960_at	HGF	hepatocyte growth factor (hepapoietin A; scatter factor)	down	2.58
209961_s_at	HGF	hepatocyte growth factor (hepapoietin A; scatter factor)	down	1.79
210755_at	HGF	hepatocyte growth factor (hepapoietin A; scatter factor)	down	2.00
210997_at	HGF	hepatocyte growth factor (hepapoietin A; scatter factor)	down	2.32
210998_s_at	HGF	hepatocyte growth factor (hepapoietin A; scatter factor)	down	2.01
205425_at	HIP1	huntingtin interacting protein 1	up	1.65
226364_at	HIP1	huntingtin interacting protein 1	up	1.89
209398_at	HIST1H1C	histone cluster 1, H1c	down	2.36
208546_x_at	HIST1H2BH	histone cluster 1, H2bh	down	1.53
214290_s_at	HIST2H2AA3 //,	histone cluster 2, H2aa3 /// histone cluster 2, H2aa4	down	2.32
218280_x_at	HIST2H2AA3 //,	histone cluster 2, H2aa3 /// histone cluster 2, H2aa4	down	2.09
221582_at	HIST3H2A	histone cluster 3, H2a	down	1.83
208894_at	HLA-DRA	major histocompatibility complex, class II, DR alpha	up	1.60
205822_s_at	HMGCS1	3-hydroxy-3-methylglutaryl-CoA synthase 1 (soluble)	down	1.57
207456_at	HNF4G	hepatocyte nuclear factor 4, gamma	up	1.72
232271_at	HNF4G	hepatocyte nuclear factor 4, gamma	up	2.53

232004_at	HNRNPR	heterogeneous nuclear ribonucleoprotein R	up	1.57
222222_s_at	HOMER3	homer homolog 3 (Drosophila)	down	1.53
227361_at	HS3ST3B1	heparan sulfate (glucosamine) 3-O-sulfotransferase 3B1	down	2.02
208937_s_at	ID1	inhibitor of DNA binding 1, dominant negative helix-loop-helix protein	up	1.66
210045_at	IDH2	isocitrate dehydrogenase 2 (NADP+), mitochondrial	up	2.17
210046_s_at	IDH2	isocitrate dehydrogenase 2 (NADP+), mitochondrial	up	2.16
229450_at	IFIT3	interferon-induced protein with tetratricopeptide repeats 3	up	1.56
214022_s_at	IFITM1	interferon induced transmembrane protein 1 (9-27)	up	1.59
201162_at	IGFBP7	insulin-like growth factor binding protein 7	down	1.87
201163_s_at	IGFBP7	insulin-like growth factor binding protein 7	down	2.44
215118_s_at	IGHA1	Immunoglobulin heavy constant alpha 1	up	1.64
1558438_a_at	IGHE	immunoglobulin heavy constant epsilon	up	1.64
228518_at	IGHG1 /// IGHN	immunoglobulin heavy constant gamma 1 (G1m marker) /// immunoglobul	up	1.97
212592_at	IGJ	immunoglobulin J polypeptide, linker protein for immunoglobulin alpha anc	up	1.64
223807_at	IGSF1	immunoglobulin superfamily, member 1	down	1.85
228375_at	IGSF11	immunoglobulin superfamily, member 11	down	1.84
207160_at	IL12A	interleukin 12A (natural killer cell stimulatory factor 1, cytotoxic lymphocyt	down	1.58
201887_at	IL13RA1	interleukin 13 receptor, alpha 1	up	1.52
222062_at	IL27RA	interleukin 27 receptor, alpha	up	1.76
205945_at	IL6R	interleukin 6 receptor	down	3.29
217489_s_at	IL6R	interleukin 6 receptor	down	2.46
226333_at	IL6R	interleukin 6 receptor	down	2.55
1568695_s_at	INTS6	integrator complex subunit 6	up	1.52
213785_at	IPO9	importin 9	down	1.52
216986_s_at	IRF4	interferon regulatory factor 4	down	1.50
215734_at	IZUMO4	IZUMO family member 4	down	1.58
226267_at	JDP2	Jun dimerization protein 2	up	1.58
213005_s_at	KANK1	KN motif and ankyrin repeat domains 1	down	1.57
213478_at	KAZ	kazrin	down	1.62
229144_at	KAZ	kazrin	down	1.55
239835_at	KBTBD8	kelch repeat and BTB (POZ) domain containing 8	down	1.72
210036_s_at	KCNH2	potassium voltage-gated channel, subfamily H (eag-related), member 2	up	1.97
229850_at	KDSR	3-ketodihydrosphingosine reductase	up	1.98
1558279_a_at	KDSR	3-ketodihydrosphingosine reductase	up	1.56
214185_at	KHDRBS1	KH domain containing, RNA binding, signal transduction associated 1	up	1.72
209781_s_at	KHDRBS3	KH domain containing, RNA binding, signal transduction associated 3	up	1.93
213358_at	KIAA0802	KIAA0802	up	1.55
231807_at	KIAA1217	KIAA1217	up	1.53
212453_at	KIAA1279	KIAA1279	down	1.95
222139_at	KIAA1466	KIAA1466 gene	up	1.62
228565_at	KIAA1804	mixed lineage kinase 4	down	1.77
227435_at	KIAA2018	KIAA2018	down	1.51
242517_at	KISS1R	KISS1 receptor	up	1.76
219371_s_at	KLF2	Kruppel-like factor 2 (lung)	down	1.88
229310_at	KLHL29	kelch-like 29 (Drosophila)	down	1.62
227565_at	KLHL5	kelch-like 5 (Drosophila)	down	1.59
232297_at	KLHL5	kelch-like 5 (Drosophila)	down	1.51
220238_s_at	KLHL7	kelch-like 7 (Drosophila)	up	1.54
231849_at	KRT80	keratin 80	up	2.03
221581_s_at	LAT2	linker for activation of T cells family, member 2	up	1.53
1569469_a_at	LHX8	LIM homeobox 8	down	2.91
211336_x_at	LILRB1	leukocyte immunoglobulin-like receptor, subfamily B (with TM and ITIM do	down	1.66
229937_x_at	LILRB1	Leukocyte immunoglobulin-like receptor, subfamily B (with TM and ITIM do	down	1.96
1554600_s_at	LMNA	lamin A/C	down	1.57
227181_at	LNP1	leukemia NUP98 fusion partner 1	down	1.63
229872_s_at	LOC100132999	hypothetical LOC100132999	up	1.68

1556301_at	LOC100287015	Hypothetical protein LOC100287015	up	1.50
235147_at	LOC100288092	Hypothetical protein LOC100288092	up	2.04
230277_at	LOC100289187	transmembrane protein 225-like /// hypothetical protein LOC100506199	up	1.65
52940_at	LOC100294402	single Ig IL-1-related receptor-like /// single immunoglobulin and toll-interle	down	1.52
235171_at	LOC100505501	hypothetical LOC100505501	down	1.61
231443_at	LOC100505644	hypothetical LOC100505644	down	1.63
241262_at	LOC100505728	hypothetical LOC100505728	down	2.19
238898_at	LOC100505730	hypothetical LOC100505730 /// hypothetical LOC100509599	up	1.55
229815_at	LOC100505894	hypothetical LOC100505894	down	1.76
230710_at	LOC100506211	hypothetical LOC100506211	down	1.57
228493_at	LOC100506992	hypothetical LOC100506992	up	1.53
230249_at	LOC100507185	hypothetical LOC100507185	up	1.83
1558256_at	LOC148189	hypothetical LOC148189	up	1.70
232645_at	LOC153684	hypothetical LOC153684	up	1.62
214162_at	LOC284244	hypothetical protein LOC284244	up	1.95
1557359_at	LOC285758	hypothetical LOC285758	up	3.15
1560762_at	LOC285972	hypothetical LOC285972	up	1.61
232298_at	LOC401093	hypothetical LOC401093	down	1.79
235173_at	LOC401093	hypothetical LOC401093	down	1.89
225028_at	LOC550643	hypothetical LOC550643	down	1.60
242770_at	LOC642236	Similar to FRG1 protein (FSHD region gene 1 protein)	up	1.75
1561759_at	LOC645513	Hypothetical LOC645513	down	1.53
1561760_s_at	LOC645513	Hypothetical LOC645513	down	1.53
203488_at	LPHN1	latrophilin 1	down	3.35
219145_at	LPHN1	latrophilin 1	down	1.90
47560_at	LPHN1	latrophilin 1	down	4.65
206953_s_at	LPHN2	latrophilin 2	up	1.54
202459_s_at	LPIN2	lipin 2	up	2.01
202460_s_at	LPIN2	lipin 2	up	1.61
216250_s_at	LPXN	leupaxin	down	1.84
204674_at	LRMP	lymphoid-restricted membrane protein	up	1.74
35974_at	LRMP	lymphoid-restricted membrane protein	up	1.94
225060_at	LRP11	low density lipoprotein receptor-related protein 11	up	2.80
233499_at	LRRC7	leucine rich repeat containing 7	down	2.40
1552666_a_at	LRRC7	leucine rich repeat containing 7	down	1.58
205036_at	LSM6	LSM6 homolog, U6 small nuclear RNA associated (S. cerevisiae)	down	1.52
1557066_at	LUC7L	LUC7-like (S. cerevisiae)	up	1.55
206276_at	LY6D	lymphocyte antigen 6 complex, locus D	down	1.56
206584_at	LY96	lymphocyte antigen 96	up	1.95
235278_at	MACROD2	MACRO domain containing 2	down	2.65
1563209_a_at	MACROD2	MACRO domain containing 2	down	2.48
1552913_at	MAGEB18	melanoma antigen family B, 18	up	1.78
232859_s_at	MAGI1	membrane associated guanylate kinase, WW and PDZ domain containing 1	down	1.55
235457_at	MAML2	mastermind-like 2 (Drosophila)	up	2.61
208786_s_at	MAP1LC3B	microtubule-associated protein 1 light chain 3 beta	up	1.55
210015_s_at	MAP2	microtubule-associated protein 2	up	1.62
203836_s_at	MAP3K5	mitogen-activated protein kinase kinase kinase 5	up	1.64
203837_at	MAP3K5	mitogen-activated protein kinase kinase kinase 5	up	1.69
202501_at	MAPRE2	microtubule-associated protein, RP/EB family, member 2	up	1.75
213489_at	MAPRE2	microtubule-associated protein, RP/EB family, member 2	up	1.67
201668_x_at	MARCKS	myristoylated alanine-rich protein kinase C substrate	up	2.13
201669_s_at	MARCKS	myristoylated alanine-rich protein kinase C substrate	up	2.15
201670_s_at	MARCKS	myristoylated alanine-rich protein kinase C substrate	up	2.46
213002_at	MARCKS	myristoylated alanine-rich protein kinase C substrate	up	1.79
225897_at	MARCKS	myristoylated alanine-rich protein kinase C substrate	up	2.57
200644_at	MARCKSL1	MARCKS-like 1	down	1.51

241813_at	MBD1	methyl-CpG binding domain protein 1	up	1.58
227839_at	MBD5	methyl-CpG binding domain protein 5	down	1.74
215663_at	MBNL1	muscleblind-like (Drosophila)	down	2.47
206132_at	MCC	mutated in colorectal cancers	down	1.71
226225_at	MCC	mutated in colorectal cancers	down	1.70
220484_at	MCOLN3	mucolipin 3	up	1.87
229797_at	MCOLN3	mucolipin 3	up	2.04
242176_at	MEF2A	Myocyte enhancer factor 2A	up	2.18
230011_at	MEI1	meiosis inhibitor 1	down	1.71
1554208_at	MEI1	meiosis inhibitor 1	down	1.60
201403_s_at	MGST3	microsomal glutathione S-transferase 3	down	1.72
212472_at	MICAL2	microtubule associated monoxygenase, calponin and LIM domain containin	up	1.98
212473_s_at	MICAL2	microtubule associated monoxygenase, calponin and LIM domain containin	up	2.83
243611_at	MICALCL	MICAL C-terminal like	up	1.84
224917_at	MIR21	microRNA 21	up	1.66
207233_s_at	MITF	microphthalmia-associated transcription factor	up	1.64
226066_at	MITF	microphthalmia-associated transcription factor	up	2.22
239468_at	MKX	mohawk homeobox	down	5.43
241902_at	MKX	mohawk homeobox	down	4.91
211071_s_at	MLLT11	myeloid/lymphoid or mixed-lineage leukemia (trithorax homolog, Drosophi	down	2.30
204917_s_at	MLLT3	myeloid/lymphoid or mixed-lineage leukemia (trithorax homolog, Drosophi	down	1.59
236208_at	MOCS2	molybdenum cofactor synthesis 2	up	1.52
218865_at	MOSC1	MOCO sulphurase C-terminal domain containing 1	down	1.67
235352_at	MR1	major histocompatibility complex, class I-related	down	1.61
223154_at	MRPL1	mitochondrial ribosomal protein L1	down	1.57
222997_s_at	MRPS21	mitochondrial ribosomal protein S21	down	1.71
225782_at	MSRB3	methionine sulfoxide reductase B3	down	2.14
225790_at	MSRB3	methionine sulfoxide reductase B3	down	2.18
238583_at	MSRB3	methionine sulfoxide reductase B3	down	2.01
1554127_s_at	MSRB3	methionine sulfoxide reductase B3	down	2.37
205106_at	MTCP1 /// MTC	mature T-cell proliferation 1 /// mature T-cell proliferation 1 neighbor	up	1.50
212248_at	MTDH	metadherin	down	1.60
227277_at	MTDH	metadherin	down	1.95
1559822_s_at	MTDH	metadherin	down	1.72
212093_s_at	MTUS1	microtubule associated tumor suppressor 1	up	1.57
212095_s_at	MTUS1	microtubule associated tumor suppressor 1	up	1.70
218687_s_at	MUC13	mucin 13, cell surface associated	up	1.57
222712_s_at	MUC13	mucin 13, cell surface associated	up	1.64
209757_s_at	MYCN	v-myc myelocytomatosis viral related oncogene, neuroblastoma derived (a	up	1.77
201976_s_at	MYO10	myosin X	up	2.76
1554026_a_at	MYO10	myosin X	up	1.79
227761_at	MYO5A	myosin VA (heavy chain 12, myoxin)	up	1.59
203215_s_at	MYO6	myosin VI	up	2.39
203216_s_at	MYO6	myosin VI	up	2.78
210480_s_at	MYO6	myosin VI	up	2.22
228523_at	NANOS1	nanos homolog 1 (Drosophila)	down	1.70
212843_at	NCAM1	neural cell adhesion molecule 1	down	3.09
227394_at	NCAM1	neural cell adhesion molecule 1	down	2.06
225786_at	NCRNA00201	non-protein coding RNA 201	down	1.61
209159_s_at	NDRG4	NDRG family member 4	up	1.94
224984_at	NFAT5	nuclear factor of activated T-cells 5, tonicity-responsive	up	1.50
217963_s_at	NGFRAP1	nerve growth factor receptor (TNFRSF16) associated protein 1	down	1.90
229491_at	NHEDC2	Na+/H+ exchanger domain containing 2	down	1.58
1564746_at	NHEDC2	Na+/H+ exchanger domain containing 2	down	1.52
225930_at	NKIRAS1	NFKB inhibitor interacting Ras-like 1	down	2.70
212739_s_at	NME4	non-metastatic cells 4, protein expressed in	down	1.60

227556_at	NME7	non-metastatic cells 7, protein expressed in (nucleoside-diphosphate kinase	up	1.58
203964_at	NMI	N-myc (and STAT) interactor	up	1.61
206023_at	NMU	neuromedin U	down	2.52
244531_at	NNT	nicotinamide nucleotide transhydrogenase	up	1.57
221348_at	NPPC	natriuretic peptide C	down	1.58
225768_at	NR1D2	nuclear receptor subfamily 1, group D, member 2	down	1.51
209505_at	NR2F1	nuclear receptor subfamily 2, group F, member 1	up	3.47
209506_s_at	NR2F1	nuclear receptor subfamily 2, group F, member 1	up	1.79
204105_s_at	NRCAM	neuronal cell adhesion molecule	up	3.28
202599_s_at	NRIP1	nuclear receptor interacting protein 1	up	1.55
202600_s_at	NRIP1	nuclear receptor interacting protein 1	up	1.68
221796_at	NTRK2	neurotrophic tyrosine kinase, receptor, type 2	down	1.70
203675_at	NUCB2	nucleobindin 2	up	1.51
220183_s_at	NUDT6	nudix (nucleoside diphosphate linked moiety X)-type motif 6	down	1.54
230329_s_at	NUDT6	nudix (nucleoside diphosphate linked moiety X)-type motif 6	down	1.82
205552_s_at	OAS1	2',5'-oligoadenylate synthetase 1, 40/46kDa	up	2.00
227492_at	OCLN	occludin	down	1.86
213125_at	OLFML2B	olfactomedin-like 2B	up	1.58
213568_at	OSR2	odd-skipped related 2 (Drosophila)	up	1.73
214615_at	P2RY10	purinergic receptor P2Y, G-protein coupled, 10	down	1.88
1553856_s_at	P2RY10	purinergic receptor P2Y, G-protein coupled, 10	down	2.41
202733_at	P4HA2	prolyl 4-hydroxylase, alpha polypeptide II	down	1.60
221868_at	PAIP2B	poly(A) binding protein interacting protein 2B	up	1.67
202336_s_at	PAM	peptidylglycine alpha-amidating monooxygenase	down	1.64
212958_x_at	PAM	peptidylglycine alpha-amidating monooxygenase	down	1.53
226649_at	PANK1	pantothenate kinase 1	down	1.55
218543_s_at	PARP12	poly (ADP-ribose) polymerase family, member 12	up	1.72
224701_at	PARP14	poly (ADP-ribose) polymerase family, member 14	up	2.08
223220_s_at	PARP9	poly (ADP-ribose) polymerase family, member 9	up	2.20
227807_at	PARP9	poly (ADP-ribose) polymerase family, member 9	up	1.73
212148_at	PBX1	pre-B-cell leukemia homeobox 1	down	2.31
212151_at	PBX1	pre-B-cell leukemia homeobox 1	down	1.83
213263_s_at	PCBP2	poly(rC) binding protein 2	up	1.73
213264_at	PCBP2	poly(rC) binding protein 2	up	1.66
205689_at	PCNXL2	pecanex-like 2 (Drosophila)	down	1.50
219295_s_at	PCOLCE2	procollagen C-endopeptidase enhancer 2	down	2.21
205559_s_at	PCSK5	proprotein convertase subtilisin/kexin type 5	down	1.73
223358_s_at	PDE7A	phosphodiesterase 7A	up	1.52
203857_s_at	PDIA5	protein disulfide isomerase family A, member 5	down	1.50
208981_at	PECAM1	platelet/endothelial cell adhesion molecule	down	2.06
208982_at	PECAM1	platelet/endothelial cell adhesion molecule	down	2.32
208983_s_at	PECAM1	platelet/endothelial cell adhesion molecule	down	1.98
217744_s_at	PERP	PERP, TP53 apoptosis effector	up	1.62
236009_at	PERP	PERP, TP53 apoptosis effector	up	1.74
228499_at	PFKFB4	6-phosphofructo-2-kinase/fructose-2,6-biphosphatase 4	down	1.84
217996_at	PHLDA1	pleckstrin homology-like domain, family A, member 1	up	1.63
217997_at	PHLDA1	pleckstrin homology-like domain, family A, member 1	up	1.91
217999_s_at	PHLDA1	pleckstrin homology-like domain, family A, member 1	up	1.68
225842_at	PHLDA1	pleckstrin homology-like domain, family A, member 1	up	1.84
212719_at	PHLPP1	PH domain and leucine rich repeat protein phosphatase 1	up	1.69
203335_at	PHYH	phytanoyl-CoA 2-hydroxylase	up	1.54
205632_s_at	PIP5K1B	phosphatidylinositol-4-phosphate 5-kinase, type I, beta	down	1.72
214717_at	PK155	hypothetical protein DKFZp434H1419	up	1.73
225380_at	PKDCC	protein kinase domain containing, cytoplasmic homolog (mouse)	down	1.77
202732_at	PKIG	protein kinase (cAMP-dependent, catalytic) inhibitor gamma	up	2.45
224758_at	PL-5283	PL-5283 protein	up	1.50

204458_at	PLA2G15	phospholipase A2, group XV	up	1.76
219014_at	PLAC8	placenta-specific 8	up	4.24
207002_s_at	PLAGL1	pleiomorphic adenoma gene-like 1	up	1.57
204613_at	PLCG2	phospholipase C, gamma 2 (phosphatidylinositol-specific)	up	1.65
205934_at	PLCL1	phospholipase C-like 1	up	2.17
1560556_a_at	PLEKHA8	Pleckstrin homology domain containing, family A (phosphoinositide binding	up	1.55
218640_s_at	PLEKHF2	pleckstrin homology domain containing, family F (with FYVE domain) meml	up	1.81
222699_s_at	PLEKHF2	pleckstrin homology domain containing, family F (with FYVE domain) meml	up	1.68
202446_s_at	PLSCR1	phospholipid scramblase 1	up	1.54
206470_at	PLXNC1	plexin C1	up	1.52
204285_s_at	PMAIP1	phorbol-12-myristate-13-acetate-induced protein 1	up	1.66
204286_s_at	PMAIP1	phorbol-12-myristate-13-acetate-induced protein 1	up	1.84
217875_s_at	PMEPA1	prostate transmembrane protein, androgen induced 1	down	2.52
222449_at	PMEPA1	prostate transmembrane protein, androgen induced 1	down	2.24
222450_at	PMEPA1	prostate transmembrane protein, androgen induced 1	down	2.23
202337_at	PMF1	polyamine-modulated factor 1	down	1.65
218224_at	PNMA1	paraneoplastic antigen MA1	up	2.06
242455_at	POU3F2	POU class 3 homeobox 2	up	1.95
220741_s_at	PPA2	pyrophosphatase (inorganic) 2	down	1.56
1556285_s_at	PPA2	pyrophosphatase (inorganic) 2	down	1.52
209433_s_at	PPAT	phosphoribosyl pyrophosphate amidotransferase	down	1.53
236302_at	PPM1E	protein phosphatase, Mg2+/Mn2+ dependent, 1E	up	2.14
225066_at	PPP2R2D	protein phosphatase 2, regulatory subunit B, delta	down	1.54
202741_at	PRKACB	protein kinase, cAMP-dependent, catalytic, beta	up	2.17
202742_s_at	PRKACB	protein kinase, cAMP-dependent, catalytic, beta	up	2.62
235780_at	PRKACB	protein kinase, cAMP-dependent, catalytic, beta	up	2.12
207957_s_at	PRKCB	protein kinase C, beta	up	2.21
209685_s_at	PRKCB	protein kinase C, beta	up	1.79
227817_at	PRKCB	protein kinase C, beta	up	2.08
227824_at	PRKCB	protein kinase C, beta	up	1.54
219168_s_at	PRR5	proline rich 5 (renal)	up	1.84
47069_at	PRR5	proline rich 5 (renal)	up	1.66
227126_at	PTPRG	protein tyrosine phosphatase, receptor type, G	up	1.68
205336_at	PVALB	parvalbumin	up	1.64
212636_at	QKI	quaking homolog, KH domain RNA binding (mouse)	up	1.62
219681_s_at	RAB11FIP1	RAB11 family interacting protein 1 (class I)	up	1.96
219562_at	RAB26	RAB26, member RAS oncogene family	down	1.59
222846_at	RAB8B	RAB8B, member RAS oncogene family	up	1.54
219125_s_at	RAG1AP1	recombination activating gene 1 activating protein 1	down	1.61
209444_at	RAP1GDS1	RAP1, GTP-GDP dissociation stimulator 1	down	1.93
217457_s_at	RAP1GDS1	RAP1, GTP-GDP dissociation stimulator 1	down	1.78
229905_at	RAP1GDS1	RAP1, GTP-GDP dissociation stimulator 1	down	1.62
204070_at	RARRES3	retinoic acid receptor responder (tazarotene induced) 3	up	1.76
208534_s_at	RASA4 /// RASA	RAS p21 protein activator 4 /// RAS p21 protein activator 4 pseudogene	up	1.50
205801_s_at	RASGRP3	RAS guanyl releasing protein 3 (calcium and DAG-regulated)	up	1.83
220680_at	RAVER2	ribonucleoprotein, PTB-binding 2	up	1.74
225396_at	RBBP4	retinoblastoma binding protein 4	up	1.56
207713_s_at	RBCK1	RanBP-type and C3HC4-type zinc finger containing 1	up	1.56
218035_s_at	RBM47	RNA binding motif protein 47	down	1.84
222496_s_at	RBM47	RNA binding motif protein 47	down	1.52
203498_at	RCAN2	regulator of calcineurin 2	up	1.95
226272_at	RCAN3	RCAN family member 3	up	1.51
226021_at	RDH10	retinol dehydrogenase 10 (all-trans)	down	1.65
1552378_s_at	RDH10	retinol dehydrogenase 10 (all-trans)	down	1.54
228980_at	RFFL	ring finger and FYVE-like domain containing 1	up	1.71
1552651_a_at	RFFL	ring finger and FYVE-like domain containing 1	up	1.61

229431_at	RFXAP	regulatory factor X-associated protein	up	1.60
209324_s_at	RGS16	regulator of G-protein signaling 16	down	1.56
209325_s_at	RGS16	regulator of G-protein signaling 16	down	1.51
227633_at	RHEB	Ras homolog enriched in brain	up	1.50
202975_s_at	RHOBTB3	Rho-related BTB domain containing 3	up	1.58
202976_s_at	RHOBTB3	Rho-related BTB domain containing 3	up	1.58
216048_s_at	RHOBTB3	Rho-related BTB domain containing 3	up	1.72
216049_at	RHOBTB3	Rho-related BTB domain containing 3	up	1.56
225202_at	RHOBTB3	Rho-related BTB domain containing 3	up	1.63
236293_at	RHOH	ras homolog gene family, member H	down	1.57
241771_at	RIMBP2	RIMS binding protein 2	down	1.54
212724_at	RND3	Rho family GTPase 3	down	1.59
217865_at	RNF130	ring finger protein 130	down	1.61
204040_at	RNF144A	ring finger protein 144A	up	1.66
227726_at	RNF166	ring finger protein 166	up	1.54
238026_at	RPL35A	ribosomal protein L35a	down	1.79
228566_at	RPRD1A	Regulation of nuclear pre-mRNA domain containing 1A	up	1.51
218909_at	RPS6KC1	ribosomal protein S6 kinase, 52kDa, polypeptide 1	down	1.60
221523_s_at	RRAGD	Ras-related GTP binding D	up	1.50
230093_at	RSPH1	radial spoke head 1 homolog (Chlamydomonas)	down	1.53
204198_s_at	RUNX3	runt-related transcription factor 3	down	1.50
214044_at	RYR2	ryanodine receptor 2 (cardiac)	down	1.52
200872_at	S100A10	S100 calcium binding protein A10	down	1.53
229402_at	SAMD13	sterile alpha motif domain containing 13	up	1.83
1559883_s_at	SAMHD1	SAM domain and HD domain 1	up	1.51
211162_x_at	SCD	stearoyl-CoA desaturase (delta-9-desaturase)	down	1.58
211708_s_at	SCD	stearoyl-CoA desaturase (delta-9-desaturase)	down	1.57
226923_at	SCFD2	sec1 family domain containing 2	down	1.55
236487_at	SCLT1	sodium channel and clathrin linker 1	down	1.51
205464_at	SCNN1B	sodium channel, nonvoltage-gated 1, beta	up	2.00
202004_x_at	SDHC	succinate dehydrogenase complex, subunit C, integral membrane protein, 1	down	2.52
210131_x_at	SDHC	succinate dehydrogenase complex, subunit C, integral membrane protein, 1	down	2.77
215088_s_at	SDHC	succinate dehydrogenase complex, subunit C, integral membrane protein, 1	down	2.50
216591_s_at	SDHC	succinate dehydrogenase complex, subunit C, integral membrane protein, 1	down	3.38
228274_at	SDSL	serine dehydratase-like	up	1.64
209879_at	SELPLG	selectin P ligand	down	1.61
206805_at	SEMA3A	sema domain, immunoglobulin domain (Ig), short basic domain, secreted, (:	up	1.83
201427_s_at	SEPP1	selenoprotein P, plasma, 1	up	6.14
231669_at	SEPP1	Selenoprotein P, plasma, 1	up	2.59
232183_at	SERAC1	serine active site containing 1	up	1.60
223196_s_at	SESN2	sestrin 2	up	1.56
235683_at	SESN3	sestrin 3	up	1.81
205120_s_at	SGCB	sarcoglycan, beta (43kDa dystrophin-associated glycoprotein)	down	1.88
226112_at	SGCB	sarcoglycan, beta (43kDa dystrophin-associated glycoprotein)	down	1.54
228584_at	SGCB	sarcoglycan, beta (43kDa dystrophin-associated glycoprotein)	down	1.67
221268_s_at	SGPP1	sphingosine-1-phosphate phosphatase 1	up	1.63
225354_s_at	SH3BGRL2	SH3 domain binding glutamic acid-rich protein like 2	up	1.66
201810_s_at	SH3BP5	SH3-domain binding protein 5 (BTK-associated)	up	1.62
201811_x_at	SH3BP5	SH3-domain binding protein 5 (BTK-associated)	up	2.22
225548_at	SHROOM3	shroom family member 3	down	1.53
219159_s_at	SLAMF7	SLAM family member 7	down	1.91
222838_at	SLAMF7	SLAM family member 7	down	2.19
234306_s_at	SLAMF7	SLAM family member 7	down	1.90
205316_at	SLC15A2	solute carrier family 15 (H+/peptide transporter), member 2	down	2.10
205317_s_at	SLC15A2	solute carrier family 15 (H+/peptide transporter), member 2	down	1.61
240159_at	SLC15A2	solute carrier family 15 (H+/peptide transporter), member 2	down	1.69

227506_at	SLC16A9	solute carrier family 16, member 9 (monocarboxylic acid transporter 9)	down	2.76
220123_at	SLC35F5	solute carrier family 35, member F5	up	1.67
225872_at	SLC35F5	solute carrier family 35, member F5	up	1.52
226629_at	SLC43A2	solute carrier family 43, member 2	up	1.62
228486_at	SLC44A1	solute carrier family 44, member 1	down	1.72
219525_at	SLC47A1	solute carrier family 47, member 1	up	1.74
204588_s_at	SLC7A7	solute carrier family 7 (cationic amino acid transporter, y+ system), membe	up	3.97
215043_s_at	SMA4 /// SMA5	glucuronidase, beta pseudogene /// glucuronidase, beta pseudogene	down	1.52
212579_at	SMCHD1	structural maintenance of chromosomes flexible hinge domain containing 1	up	1.52
219695_at	SMPD3	sphingomyelin phosphodiesterase 3, neutral membrane (neutral sphingomy	up	2.10
218404_at	SNX10	sorting nexin 10	up	1.58
212560_at	SORL1	sortilin-related receptor, L(DLR class) A repeats-containing	up	1.77
214791_at	SP140L	SP140 nuclear body protein-like	up	1.54
219888_at	SPAG4	sperm associated antigen 4	up	1.70
212458_at	SPRED2	sprouty-related, EVH1 domain containing 2	up	1.51
219205_at	SRR	serine racemase	up	1.52
222844_s_at	SRR	serine racemase	up	1.78
230836_at	ST8SIA4	ST8 alpha-N-acetyl-neuraminide alpha-2,8-sialyltransferase 4	up	1.53
205542_at	STEAP1	six transmembrane epithelial antigen of the prostate 1	up	2.09
222557_at	STMN3	stathmin-like 3	up	3.02
212353_at	SULF1	sulfatase 1	down	1.54
212354_at	SULF1	sulfatase 1	down	1.59
226850_at	SUMF1	sulfatase modifying factor 1	down	1.67
243139_at	SV2C	synaptic vesicle glycoprotein 2C	up	1.79
218692_at	SYBU	syntabulin (syntaxin-interacting)	down	1.65
202761_s_at	SYNE2	spectrin repeat containing, nuclear envelope 2	up	1.71
210053_at	TAF5	TAF5 RNA polymerase II, TATA box binding protein (TBP)-associated factor,	down	1.51
225308_s_at	TANC1	tetratricopeptide repeat, ankyrin repeat and coiled-coil containing 1	up	1.55
222116_s_at	TBC1D16	TBC1 domain family, member 16	down	1.60
228488_at	TBC1D16	TBC1 domain family, member 16	down	1.51
209152_s_at	TCF3	transcription factor 3 (E2A immunoglobulin enhancer binding factors E12/E	down	1.58
212387_at	TCF4	transcription factor 4	up	1.51
222146_s_at	TCF4	transcription factor 4	up	1.58
228837_at	TCF4	transcription factor 4	up	2.11
204043_at	TCN2	transcobalamin II	up	2.04
221035_s_at	TEX14	testis expressed 14	down	1.55
241367_at	TEX19	testis expressed 19	up	1.93
204731_at	TGFBR3	transforming growth factor, beta receptor III	up	1.72
203313_s_at	TGIF1	TGFB-induced factor homeobox 1	up	1.56
229253_at	THEM4	thioesterase superfamily member 4	down	1.62
228619_x_at	TIPRL	TIP41, TOR signaling pathway regulator-like (S. cerevisiae)	down	1.61
204227_s_at	TK2	thymidine kinase 2, mitochondrial	up	1.69
204276_at	TK2	thymidine kinase 2, mitochondrial	up	1.73
204872_at	TLE4	transducin-like enhancer of split 4 (E(sp1) homolog, Drosophila)	up	1.65
216997_x_at	TLE4	transducin-like enhancer of split 4 (E(sp1) homolog, Drosophila)	up	1.80
233575_s_at	TLE4	transducin-like enhancer of split 4 (E(sp1) homolog, Drosophila)	up	1.90
221060_s_at	TLR4	toll-like receptor 4	up	1.86
224341_x_at	TLR4	toll-like receptor 4	up	1.55
1552798_a_at	TLR4	toll-like receptor 4	up	1.68
219892_at	TM6SF1	transmembrane 6 superfamily member 1	up	1.54
226478_at	TM7SF3	transmembrane 7 superfamily member 3	up	1.58
1552302_at	TMEM106A	transmembrane protein 106A	up	1.99
1552303_a_at	TMEM106A	transmembrane protein 106A	up	1.66
227172_at	TMEM116	transmembrane protein 116	up	1.59
243708_at	TMEM132E	transmembrane protein 132E	up	1.51
218999_at	TMEM140	transmembrane protein 140	up	2.05

227890_at	TMEM198	transmembrane protein 198	up	1.62
222752_s_at	TMEM206	transmembrane protein 206	down	1.70
222896_at	TMEM38A	transmembrane protein 38A	down	2.45
219410_at	TMEM45A	transmembrane protein 45A	down	1.53
209656_s_at	TMEM47	transmembrane protein 47	up	1.58
226338_at	TMEM55A	transmembrane protein 55A	up	1.52
203661_s_at	TMOD1	tropomodulin 1	up	1.63
202644_s_at	TNFAIP3	tumor necrosis factor, alpha-induced protein 3	up	1.57
223851_s_at	TNFRSF18	tumor necrosis factor receptor superfamily, member 18	up	1.91
224553_s_at	TNFRSF18	tumor necrosis factor receptor superfamily, member 18	up	1.90
202687_s_at	TNFSF10	tumor necrosis factor (ligand) superfamily, member 10	up	1.54
213201_s_at	TNNT1	troponin T type 1 (skeletal, slow)	down	1.61
204529_s_at	TOX	thymocyte selection-associated high mobility group box	down	1.64
213888_s_at	TRAF3IP3	TRAF3 interacting protein 3	down	1.66
1554287_at	TRIM4	tripartite motif-containing 4	up	1.55
215047_at	TRIM58	tripartite motif-containing 58	down	1.63
214908_s_at	TRRAP	transformation/transcription domain-associated protein	up	1.73
215111_s_at	TSC22D1	TSC22 domain family, member 1	up	1.86
219274_at	TSPAN12	tetraspanin 12	up	1.52
230626_at	TSPAN12	tetraspanin 12	up	1.69
212928_at	TSPYL4	TSPY-like 4	up	1.58
1569472_s_at	TTC3	tetratricopeptide repeat domain 3	up	1.52
210652_s_at	TTC39A	tetratricopeptide repeat domain 39A	up	1.68
226152_at	TTC7B	tetratricopeptide repeat domain 7B	up	1.62
219882_at	TTLL7	tubulin tyrosine ligase-like family, member 7	down	1.95
228724_at	TTLL7	tubulin tyrosine ligase-like family, member 7	down	1.58
244839_at	TTN	titin	up	1.84
223741_s_at	TTYH2	tweety homolog 2 (Drosophila)	down	1.62
204141_at	TUBB2A	tubulin, beta 2A	up	1.55
209372_x_at	TUBB2A /// TUI	tubulin, beta 2A /// tubulin, beta 2B	up	1.84
214023_x_at	TUBB2B	tubulin, beta 2B	up	2.55
213943_at	TWIST1	twist homolog 1 (Drosophila)	up	1.68
235749_at	UGGT2	UDP-glucose glycoprotein glucosyltransferase 2	up	1.58
1555561_a_at	UGGT2	UDP-glucose glycoprotein glucosyltransferase 2	up	1.53
235003_at	UHMK1	U2AF homology motif (UHM) kinase 1	down	1.57
1556095_at	UNC13C	unc-13 homolog C (C. elegans)	down	1.74
1556096_s_at	UNC13C	unc-13 homolog C (C. elegans)	down	2.45
1569969_a_at	UNC13C	unc-13 homolog C (C. elegans)	down	1.93
203031_s_at	UROS	uroporphyrinogen III synthase	down	1.59
223167_s_at	USP25	ubiquitin specific peptidase 25	up	1.55
213022_s_at	UTRN	utrophin	up	2.40
225093_at	UTRN	utrophin	up	2.62
228912_at	VIL1	villin 1	up	1.67
209822_s_at	VLDLR	very low density lipoprotein receptor	up	1.69
204165_at	WASF1	WAS protein family, member 1	down	1.56
230152_at	WDR52	WD repeat domain 52	down	1.53
1556429_a_at	WDR67	WD repeat domain 67	up	1.70
228953_at	WHAMM	WAS protein homolog associated with actin, golgi membranes and microtul	up	1.50
228949_at	WLS	wntless homolog (Drosophila)	down	1.67
215150_at	YOD1	YOD1 OTU deubiquinating enzyme 1 homolog (S. cerevisiae)	down	1.91
227309_at	YOD1	YOD1 OTU deubiquinating enzyme 1 homolog (S. cerevisiae)	down	8.67
219312_s_at	ZBTB10	zinc finger and BTB domain containing 10	up	1.68
222863_at	ZBTB10	zinc finger and BTB domain containing 10	up	1.77
228562_at	ZBTB10	zinc finger and BTB domain containing 10	up	1.92
233899_x_at	ZBTB10	zinc finger and BTB domain containing 10	up	1.99
236105_at	ZBTB10	zinc finger and BTB domain containing 10	up	1.86

231899_at	ZC3H12C	zinc finger CCCH-type containing 12C	up	1.77
219062_s_at	ZCCHC2	zinc finger, CCHC domain containing 2	up	1.74
222816_s_at	ZCCHC2	zinc finger, CCHC domain containing 2	up	1.59
1552557_a_at	ZDHHC15	zinc finger, DHHC-type containing 15	up	1.91
226124_at	ZFP90	zinc finger protein 90 homolog (mouse)	up	1.76
235698_at	ZFP90	zinc finger protein 90 homolog (mouse)	up	1.89
219778_at	ZFPM2	zinc finger protein, multitype 2	down	1.54
203651_at	ZFYVE16	zinc finger, FYVE domain containing 16	up	1.53
218645_at	ZNF277	zinc finger protein 277	up	1.53
1555193_a_at	ZNF277	zinc finger protein 277	up	1.62
218401_s_at	ZNF281	zinc finger protein 281	down	1.67
233082_at	ZNF630	zinc finger protein 630	up	1.53
222624_s_at	ZNF639	zinc finger protein 639	down	1.52
223302_s_at	ZNF655	zinc finger protein 655	up	1.70
225945_at	ZNF655	zinc finger protein 655	up	1.94
1554726_at	ZNF655	zinc finger protein 655	up	1.89
1553247_a_at	ZNF709	zinc finger protein 709	up	1.55
238149_at	ZNF818P	zinc finger protein 818, pseudogene	up	1.60
1569241_a_at	ZNF93	zinc finger protein 93	up	1.54
237335_at	ZP1	zona pellucida glycoprotein 1 (sperm receptor)	up	1.98
213448_at			down	1.55
213657_s_at			up	1.77
213658_at			up	1.68
216247_at			up	1.53
37590_g_at			up	1.90
225123_at			up	2.62
225567_at			up	1.53
225722_at			down	1.66
226756_at			up	1.84
226993_at			up	1.59
227290_at			up	1.53
227368_at			up	1.63
227531_at			down	1.52
227547_at			up	1.73
227682_at			up	1.52
228297_at			up	1.60
228478_at			up	1.53
228812_at			up	1.65
228987_at			up	1.57
229202_at			down	2.29
229575_at			up	1.52
229580_at			down	1.90
229620_at			up	2.08
230319_at			down	1.79
230416_at			down	1.65
230655_at			up	1.72
230778_at			up	1.93
230795_at			down	1.52
230917_at			up	2.31
231644_at			up	1.51
233016_at			down	1.69
233401_at			up	1.62
235008_at			up	1.55
235242_at			up	1.55
235286_at			up	1.87
235785_at			up	1.68

235947_at	down	1.59
236198_at	up	1.70
236277_at	up	1.84
236280_at	down	2.54
236307_at	up	1.98
236310_at	down	1.51
236330_at	down	1.67
236660_at	up	1.58
237035_at	up	1.75
237203_at	down	1.58
237563_s_at	down	1.73
238191_at	down	1.76
239253_at	up	1.53
239264_at	up	1.54
239423_at	up	1.64
239729_at	up	1.91
239767_at	down	1.80
239945_at	up	1.67
239999_at	up	1.74
240064_at	down	1.66
240118_at	down	1.93
240143_at	up	1.51
241416_at	up	1.50
241845_at	down	1.52
242358_at	up	1.60
242494_at	up	1.55
242598_at	up	1.51
242801_at	up	1.88
243049_at	up	1.56
243465_at	up	1.87
243489_at	down	2.03
243543_at	down	1.66
243718_at	down	1.59
243918_at	up	1.50
243931_at	down	1.50
244290_at	up	1.53
244414_at	up	1.59
1556211_a_at	up	1.54
1556602_at	down	1.61
1556911_at	up	2.14
1558237_x_at	up	1.62
1558409_at	up	1.61
1558410_s_at	up	1.60
1558522_at	up	1.50
1558801_at	up	1.53
1561195_at	up	1.53
1561511_at	down	2.21
1566491_at	up	2.10
1566698_at	up	1.56
1568781_at	down	1.51
1570561_at	up	126.45

Supplementary Table S2. Top KLF4 profile neighbors across 304 MM patient samples in the Multiple Myeloma Research Consortium reference collection dataset (GEO accession number GSE26760) with the annotation term "autophagy".

Probe Set ID	r ≥ 0.2 wrt 221841_s_at (KLF4)	Symbol	Gene ID
200620_at	0.27	TMEM59	9528
201848_s_at	0.21	BNIP3	664
201849_at	0.24	BNIP3	664
202877_s_at	0.30	CD93	22918
202878_s_at	0.26	CD93	22918
204006_s_at	0.30	FCGR3A///FCGR3B	2214///2215
204007_at	0.31	FCGR3B	2215
200645_at	0.24	GABARAP	11337
208868_s_at	0.25	GABARAPL1	23710
208869_s_at	0.29	GABARAPL1	23710
211458_s_at	0.30	GABARAPL1///GABARAPL3	23710///23766
208786_s_at	0.21	MAP1LC3B	81631
209733_at	0.22	MID2	11043
1559822_s_at	0.20	MTDH	92140
207075_at	0.22	NLRP3	114548
216015_s_at	0.24	NLRP3	114548
209018_s_at	0.27	PINK1	65018
209019_s_at	0.27	PINK1	65018
227892_at	0.24	PRKAA2	5563
242531_at	0.21	RRAGC	64121
202917_s_at	0.30	S100A8	6279
214370_at	0.27	S100A8	6279
203535_at	0.31	S100A9	6280
222258_s_at	0.23	SH3BP4	23677
1558331_at	0.25	SIRT2	22933
244804_at	0.21	SQSTM1	8878
241018_at	0.22	TMEM59	9528
212602_at	0.33	WDFY3	23001
212606_at	0.30	WDFY3	23001
238660_at	0.23	WDFY3	23001
218810_at	0.27	ZC3H12A	80149

Complete list @ http://amigo.geneontology.org/amigo/search/bioentity?q=*:*&fq=regulates_closure:%22GO:0006914%22&sfq=document_category:%22bioentity%22
